# Nature and strength of group-14 A–A′ bonds†

**DOI:** 10.1039/d3sc06215e

**Published:** 2024-01-16

**Authors:** Daniela Rodrigues Silva, Eva Blokker, J. Martijn van der Schuur, Trevor A. Hamlin, F. Matthias Bickelhaupt

**Affiliations:** a Department of Chemistry and Pharmaceutical Sciences, AIMMS, Vrije Universiteit Amsterdam De Boelelaan 1108 Amsterdam 1081 HZ The Netherlands f.m.bickelhaupt@vu.nl https://www.theochem.nl; b Polymer Specialties, Nouryon Zutphenseweg 10 Deventer 7418 AJ The Netherlands; c Institute of Molecules and Materials, Radboud University Heyendaalseweg 135 Nijmegen 6525 AJ The Netherlands; d Department of Chemical Sciences, University of Johannesburg Auckland Park Johannesburg 2006 South Africa

## Abstract

We have quantum chemically investigated the nature and stability of C–C and Si–Si bonds in R_3_A–AR_3_ (A = C, Si; R_3_ = H_3_, Me_3_, Me_2_Ph, MePh_2_, Ph_3_, *t*-Bu_3_) using density functional theory (DFT). Systematic increase of steric bulk of the substituents R has opposite effects on C–C and Si–Si bonds: the former becomes weaker whereas the latter becomes stronger. Only upon going further, from R = Ph to the bulkiest R = *t*-Bu, the R_3_Si–SiR_3_ bond begins to weaken. Our bonding analyses show how different behavior upon increasing the steric bulk of the substituents stems from the interplay of (Pauli) repulsive and (dispersion) attractive steric mechanisms. Extension of our analyses to other model systems shows that C–Si bonds display behavior that is in between that of C–C and Si–Si bonds. Further increasing the size of the group-14 atoms from C–C and Si–Si to Ge–Ge, Sn–Sn and Pb–Pb leads to a further decrease in the sensitivity of the bond strength with respect to the substituents' bulkiness. Our findings can be used as design principles for tuning A–A and A–A′ bond strengths.

## Abstract 2

Quantum-chemical analyses reveal: Si–Si bonds are longer, weaker, and react oppositely to introducing bulkier substituents than C–C bonds.

## Graphical abstract

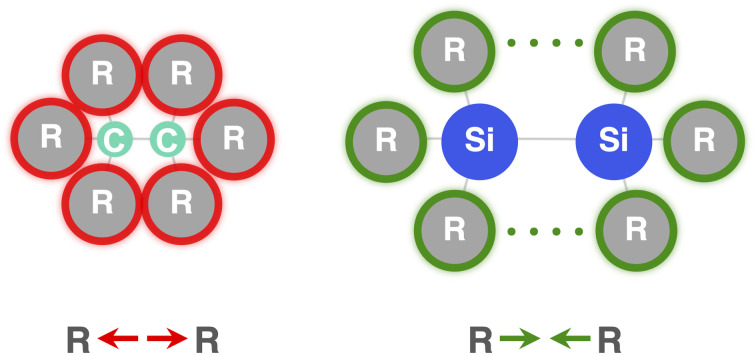

## Introduction

The carbon atom is not the most abundant element on earth. Yet, it plays a fundamental role in human life as it is the universal connector of organic compounds.^1^ The R_3_C–CR_3_ bond is remarkably strong and stable compared to other homodiatomic bonds.^2^ Furthermore, its strength can be easily tuned by modulation of the steric properties of the R groups. Thus, the R_3_C–CR_3_ bond is gradually weakened as the number and size of the R groups increase (*e.g.*, from hydrogen to methyl to ethyl to isopropyl).^3^ However, if R becomes too bulky, the R_3_C˙ radicals do not dimerize because their mutual steric repulsion becomes too large in which case alternative bonding patterns occur. A remarkable example is the so-called hexaphenylethane (Ph_3_C–CPh_3_) riddle,^4^ where two Ph_3_C˙ radicals do not dimerize to form hexaphenylethane, although believed so for many decades.^5^ Instead, they form an unsymmetrical quinoid structure (*i.e.*, {[4-(diphenylmethylene)cyclohexa-2,5-dien-1-yl]methane-triyl}tribenzene).^6^ Hexaphenylethane became over the years a theoretical construct used as a reference to explore the limits of the C–C bond length and strength.^7^ In a series of seminal works,^8^ Schreiner and coworkers showed that the C–C bond could be stabilized by increasing steric bulk in the all-*meta-tert*-butyl derivative of hexaphenylethane because of stabilizing dispersion interactions, also referred to as steric attraction.^9^

Silicon is the third-period congener of, and therefore resembles in certain aspects, carbon. At the same time, silicon also exhibits vastly different bonding capacities.^10^ For example, silicon can readily form stable hypervalent compounds while carbon does not.^11^ Interestingly, contrary to the C–C bond, the Si–Si bond is *strengthened* in hexaphenyldisilane (Ph_3_Si–SiPh_3_) compared to sterically less congested analogs (*e.g.*, H_3_Si–SiH_3_).^12^ The origin of this dichotomy has been attributed to common steric and electronic effects.^13^ Nevertheless, despite these efforts, the opposite behavior of the C–C *versus* the Si–Si bonds is still incompletely understood and lacks an overarching model that is soundly rooted in quantum mechanics.

**Fig. 1:**
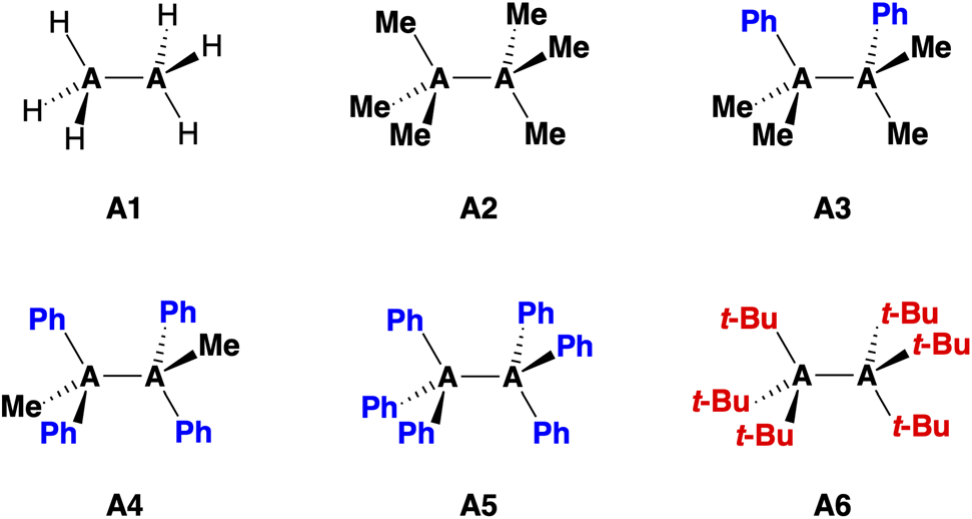
Model systems R_3_A–AR_3_ for A = C (C1–6) and A = Si (Si1–6).

Herein, we investigate the bonding mechanism in the R_3_A–AR_3_ model systems (A = C, Si; R_3_ = H_3_, Me_3_, Me_2_Ph, MePh_2_, Ph_3_, *t*-Bu_3_; see eqn (1) and Fig. 1) as a function of the A–A bond distance using the activation strain model (ASM)^14^ in conjunction with Kohn–Sham molecular orbital theory (KS-MO)^15^ and a matching energy decomposition analysis (EDA).^16^

1 R_3_A˙ + ˙AR_3_ → R_3_A–AR_3_ Δ*H* = −Δ*H*_BDE_

We wish to understand why the C–C and Si–Si bonds behave differently upon increasing the steric bulk of the substituents. The crux turns out to be the fact that steric (Pauli) repulsion between substituents is a short-range interaction^16*a*^ that is more important in the case of short bonds (*i.e.*, C–C and C–R) whereas steric (dispersion) attraction is a long-range interaction^9^ that dominates in the case of longer bonds (Si–Si and Si–R). Indeed, C–Si bonds show behavior with respect to variation in the bulkiness of substituents R which is in between that of C–C and Si–Si bonds. Also, further increasing the size of the group-14 atoms along Ge–Ge, Sn–Sn and Pb–Pb shows an additional attenuation of the sensitivity of the bond strength with respect to the substituents' steric demand. The findings that emerge from our bonding analyses on a systematic set of R_3_A–AR_3_ model systems can be used as design principles for tuning the strength of A–A and A–A′ bonds.

## Computational methods

### Computational details

All calculations were performed using the Amsterdam Density Functional (ADF) program (ADF2019.305 for the A–A and constrained R_3_A–AR_3_ bonding analyses as well as mixed R_3_C–SiR_3_ and heavier R_3_A–AR_3_ (A = Ge, Sn, Pb) systems, whereas ADF2017.111 was used for all other computations).^17^ Geometries and energies were calculated at the BLYP level of the generalized gradient approximation (GGA).^18^ The DFT-D3(BJ) method developed by Grimme and coworkers,^19^ which contains the damping function introduced by Becke and Johnson,^20^ was used to correct for dispersion interactions. Molecular orbitals (MOs) were expanded using a large, uncontracted set of slater-type orbitals (STO): TZ2P.^21^ The TZ2P basis set is of triple-*ζ* quality, augmented by two sets of polarization functions. All electrons were treated variationally. The trends and conclusions emerging from our BLYP-D3(BJ)/TZ2P computations are nicely reproduced at the M06-2X^22^/TZ2P level (see Table S1 and Fig. S1†). The radical fragments were treated with a spin-unrestricted formalism. The accuracies of the fit scheme (Zlm fit)^23^ and the integration grid (Becke grid)^24^ were set to EXCELLENT. All geometry optimizations were performed in *C*_1_ symmetry without any symmetry constraint. All optimized structures were confirmed to be true minima (no imaginary frequencies) through vibrational analyses.^25^

### Thermochemistry

Bond enthalpies at 298.15 K and 1 atm (Δ*H*) were calculated from electronic bond energies (Δ*E*) and vibrational frequencies using standard thermochemistry relations for an ideal gas, according to eqn (2):^26^

2 Δ*H* = Δ*E* + Δ*E*_trans,298_ + Δ*E*_rot,298_ + Δ*E*_vib,0_ + Δ(Δ*E*_vib,0_)_298_ + Δ(*pV*)

Here, Δ*E*_trans,298_, Δ*E*_rot,298_, and Δ*E*_vib,0_ are the differences between the reactants (*i.e.*, R_3_A˙, the radical fragments) and product (*i.e.*, R_3_A–AR_3_, the complex) in translational, rotational, and zero-point vibrational energy, respectively. Δ(Δ*E*_vib,0_)_298_ is the change in the vibrational energy difference as one goes from 0 to 298.15 K. The vibrational energy corrections are based on our frequency calculations. The molar work term Δ(*pV*) is (Δ*n*)*RT*; Δ*n* = −1 for two R_3_A˙ radicals combining to one R_3_A–AR_3_ molecule. Thermal corrections for the electronic energy are neglected.

### Activation strain model and energy decomposition analysis

In the activation strain model (ASM),^14^ the overall A–A bond energy Δ*E* [which also features in eqn (2)] between two radicals R_3_A˙ in R_3_A–AR_3_ is decomposed into two major components:

3 Δ*E* = Δ*E*_strain_ + Δ*E*_int_

Here, the strain energy Δ*E*_strain_ is the amount of energy required to deform the radical fragments from their equilibrium structure to the geometry that they acquire in the final molecule. The interaction energy Δ*E*_int_ corresponds to the actual energy change when the geometrically deformed R_3_A˙ fragments are combined to form R_3_A–AR_3_.

We further analyze the interaction energy Δ*E*_int_ within the framework of the Kohn–Sham molecular orbital (KS-MO)^15^ model by dissecting it using our canonical energy decomposition analysis (EDA)^16^ scheme into electrostatic interactions, Pauli repulsion, (attractive) orbital interactions, dispersion corrections, and spin polarization:

4 Δ*E*_int_ = Δ*V*_elstat_ + Δ*E*_Pauli_ + Δ*E*_oi_ + Δ*E*_disp_ + Δ*E*_spinpol_

The electrostatic energy Δ*V*_elstat_ corresponds to the electrostatic interactions between the unperturbed charge distribution of the radical fragments R_3_A˙, which is usually attractive. The Pauli repulsion Δ*E*_Pauli_ comprises the destabilizing interactions between occupied orbitals and is responsible for any steric repulsion. The orbital interactions Δ*E*_oi_ term accounts for electron-pair bonding (the SOMO–SOMO interaction), charge transfer (donor–acceptor interaction between an occupied orbital of one fragment with an empty orbital of the other fragment), and polarization (empty/occupied orbital mixing on one fragment due to the presence of another fragment). The dispersion energy Δ*E*_disp_ is added as a correction.^19^ Finally, the Δ*E*_spinpol_ term refers to the spin polarization of the spin-α and spin-β electrons of the deformed unrestricted fragments and is with respect to Δ*E*_int_ destabilizing (*i.e.*, the deformed unrestricted fragments without spin polarization lie consistently 2–4 kcal mol^−1^ higher in energy and therefore have a too stabilizing Δ*E*_int_; see Table S2†).^27^ The open-shell PyFrag2019 program was used to analyze the bond dissociation as a function of the R_3_A–AR_3_ distance.^28^

## Results and discussion

### General trends in bond strength

The bond enthalpies Δ*H* [eqn (1)] under standard conditions (298.15 K and 1 atm) of the R_3_A–AR_3_ model systems (A = C, Si; R_3_ = H_3_, Me_3_, Me_2_Ph, MePh_2_, Ph_3_, *t*-Bu_3_) from our BLYP-D3(BJ)/TZ2P computations are collected in Table 1. The computed trends in Δ*H*, and hence in bond strength, agree very well with available experimental data (see Table S3†).^29^ We find that the Si–Si bond in Si1 is intrinsically weaker and longer than the C–C bond in C1 (Δ*H* = −71.4 kcal mol^−1^ and *r*_Si–Si_ = 2.356 Å for Si1 and Δ*H* = −85.2 kcal mol^−1^ and *r*_C–C_ = 1.538 Å for C1). Furthermore, the R_3_C–CR_3_ bond systematically weakens and elongates as the R groups are varied from hydrogen to methyl to phenyl, for example, from −85.2 kcal mol^−1^ and 1.538 Å for C1 to −4.6 kcal mol^−1^ and 1.738 Å for C5. The opposite trend emerges for R_3_Si–SiR_3_ along the same R series, that is, the Si–Si bond becomes slightly stronger from −71.4 kcal mol^−1^ for Si1 to −78.5 kcal mol^−1^ for Si5. The Si–Si bond length varies little and is *ca.* 2.36 Å in all cases. Only with the addition of the isotropically bulky *tert*-butyl group^30^ in Si6, the Si–Si bond does significantly weaken and stretch (Δ*H* = −41.0 kcal mol^−1^ and *r*_Si–Si_ = 2.726 Å).^31^ The *t*-Bu groups in C6 encumber the dimerization of the *t*-Bu_3_C˙ radicals,^32^ which are known as persistent radicals.^33^

**Table 1:** Bond enthalpies (Δ*H*; in kcal mol^−1^), activation strain model terms (in kcal mol^−1^), and bond lengths (in Å) of the R_3_A–AR_3_ bonds (A = C, Si; R_3_ = H_3_, Me_3_, Me_2_Ph, MePh_2_, Ph_3_, *t*-Bu_3_)^a^

No.	Species^b^	Δ*H*	Δ*E*	Δ*E*_strain_	Δ*E*_int_	*r*_A–A_
C1	H_3_C–CH_3_	−85.2	−92.1	18.4	−110.4	1.538
C2	Me_3_C–CMe_3_	−64.0	−69.6	25.0	−94.6	1.597
C3 c	PhMe_2_C–CMe_2_Ph	−42.1	−46.8	39.7	−86.5	1.627
C4^d^	Ph_2_MeC–CMePh_2_	−25.9	−29.9	56.9	−86.8	1.638
C5	Ph_3_C–CPh_3_	−4.6	−7.0	65.1	−72.2	1.738
Si1	H_3_Si–SiH_3_	−71.4	−74.3	0.6	−74.8	2.356
Si2	Me_3_Si–SiMe_3_	−73.0	−75.1	0.3	−75.4	2.357
Si3^c^	PhMe_2_Si–SiMe_2_Ph	−73.8	−74.7	0.7	−75.4	2.356
Si4^d^	Ph_2_MeSi–SiMePh_2_	−74.4	−75.3	3.5	−78.8	2.353
Si5	Ph_3_Si–SiPh_3_	−78.5	−79.8	1.2	−81.0	2.358
Si6	*t*-Bu_3_Si–Si*t*-Bu_3_	−41.0	−45.8	15.0	−60.8	2.726

a Computed at BLYP-D3(BJ)/TZ2P at 298.15 K and 1 atm. All structures are staggered (for more details, see Table S4).

b C6 does not form a stable C–C electron-pair bond.

c *Gauche* conformation.

d *Anti* conformation.

The electronic bond energy Δ*E* is slightly more stabilizing than the Δ*H*, mainly due to zero-point vibrational energy effects in the latter, and always retains the same overall trends as the latter (Table 1). Therefore, to understand the origin of the aforementioned trends in C–C and Si–Si bond strengths, we analyze the features in the bonding mechanism that determine the trends in Δ*E* using the activation strain model (ASM),^14^ which decomposes Δ*E* into the strain energy Δ*E*_strain_ and the interaction energy Δ*E*_int_ (eqn (3); see Computational methods for a theoretical overview). Inspection of the ASM terms in Table 1 reveals that, in all cases, the trends in bond strength Δ*E* emerge from the trends in the interaction energy Δ*E*_int_. For example, the Si–Si bond in Si1 is weaker than the C–C bond in C1 (Δ*E* = −74.3 kcal mol^−1^ and −92.1 kcal mol^−1^, respectively) because of a less stabilizing interaction between H_3_Si˙ radicals than between H_3_C˙ radicals (Δ*E*_int_ = −74.8 kcal mol^−1^ for Si1 and −110.4 kcal mol^−1^ for C1). The weakening of the C–C bond from C1 to C5 (Δ*E* = −92.1 kcal mol^−1^ and −7.0 kcal mol^−1^, respectively) and strengthening of the Si–Si bond from Si1 to Si5 (Δ*E* = −74.3 kcal mol^−1^ and −79.8 kcal mol^−1^, respectively) also comes from the interaction energy that becomes less and more stabilizing, respectively (Δ*E*_int_ = −110.4 kcal mol^−1^ for C1 and −72.2 kcal mol^−1^ for C5, whereas Δ*E*_int_ = −74.8 kcal mol^−1^ for Si1 and −81.0 kcal mol^−1^ for Si5). Furthermore, the C–C bond strength is also determined by strain energy Δ*E*_strain_. As will be discussed later, part of the less stabilizing Δ*E*_int_ is absorbed into a more destabilizing Δ*E*_strain_, which is associated with the pyramidalization of the R_3_A˙ radicals upon formation of the R_3_A–AR_3_ bond. Below, we systematically elucidate each of these features and provide their underlying physical mechanism.

### Intrinsic bond strength

**Fig. 2:**
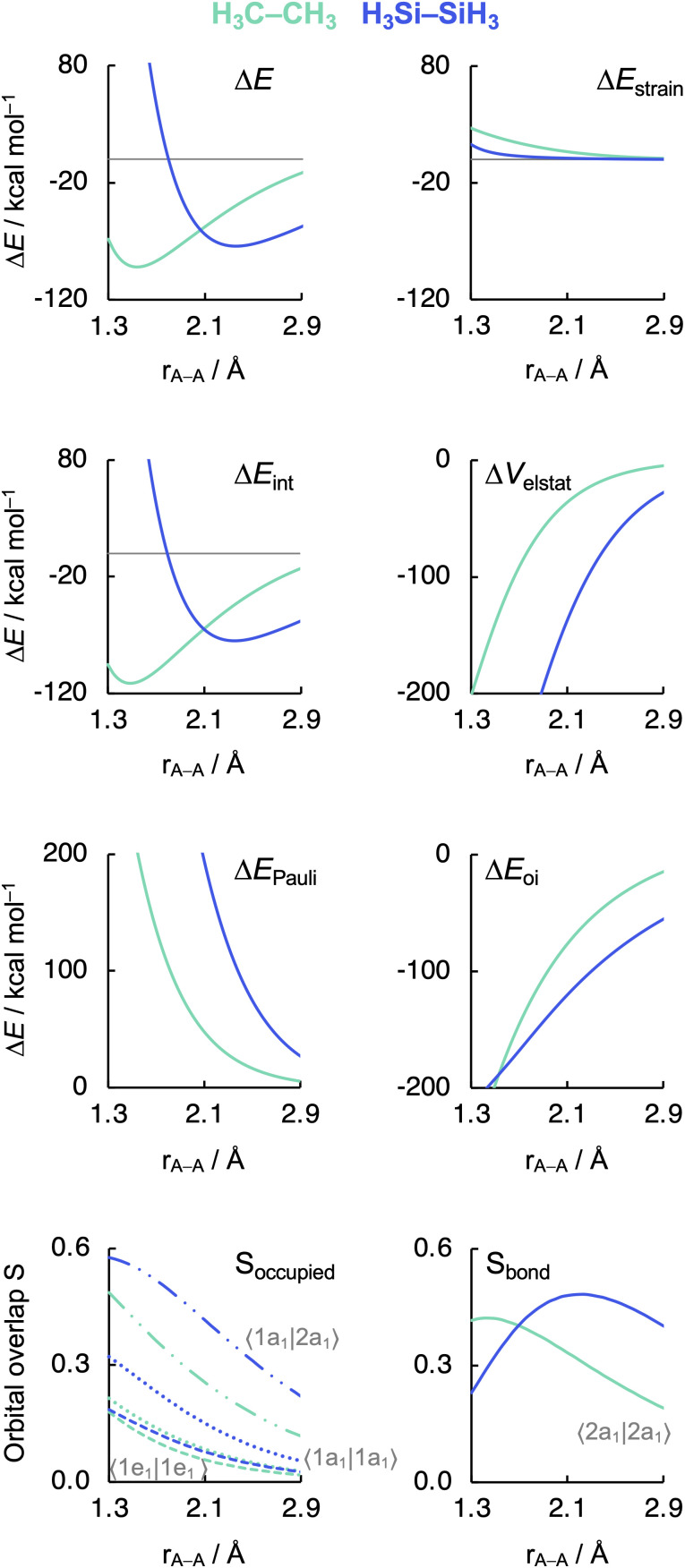
Energy decomposition analysis terms, main occupied–occupied orbital overlaps (*S*_occupied_), and SOMO–SOMO overlap (*S*_bond_) as a function of the A–A distance in H_3_A–AH_3_ (1, A = C, Si). The dispersion energy Δ*E*_disp_ is nearly constant and, therefore, not shown. See Fig. 3 for the schematic AH_3_˙ FMOs. Computed at BLYP-D3(BJ)/TZ2P. MO numbering starts at the lowest-energy valence AO combination.

Before examining the effect of substituents, we wish to understand the difference in the intrinsic strength between the C–C and Si–Si bonds in the unsubstituted, archetypal model systems ethane C1 and disilane Si1. Why is the Si–Si bond in Si1 weaker than the C–C bond in C1, *i.e.*, why is Δ*E* less stabilizing for the former than for the latter? To facilitate an equitable comparison of the studied systems to arrive at the actual causalities in their bonding mechanism, our bonding analyses are carried out as a function of the A–A bond distance from now on (Fig. 2). As will become clear in the following, our analyses reveal that the Si–Si bond in Si1 is weaker than the C–C bond in C1 due to the increase in effective atom size of Si compared to C and, thus, the increase in Pauli repulsion. This pushes the distance between the two Si atoms to a longer value and also weakens the bond compared to two C atoms. The bond weakening from C–C to Si–Si occurs despite an electron-pair bond that, at most bond distances, becomes stronger, not weaker, because of a larger bonding overlap between the spatially more extended (and diffuse) silyl SOMOs compared to the more compact methyl SOMOs. This is reminiscent of the weakening and lengthening of C–X bonds as X is varied from F to I.^34^

**Fig. 3:**
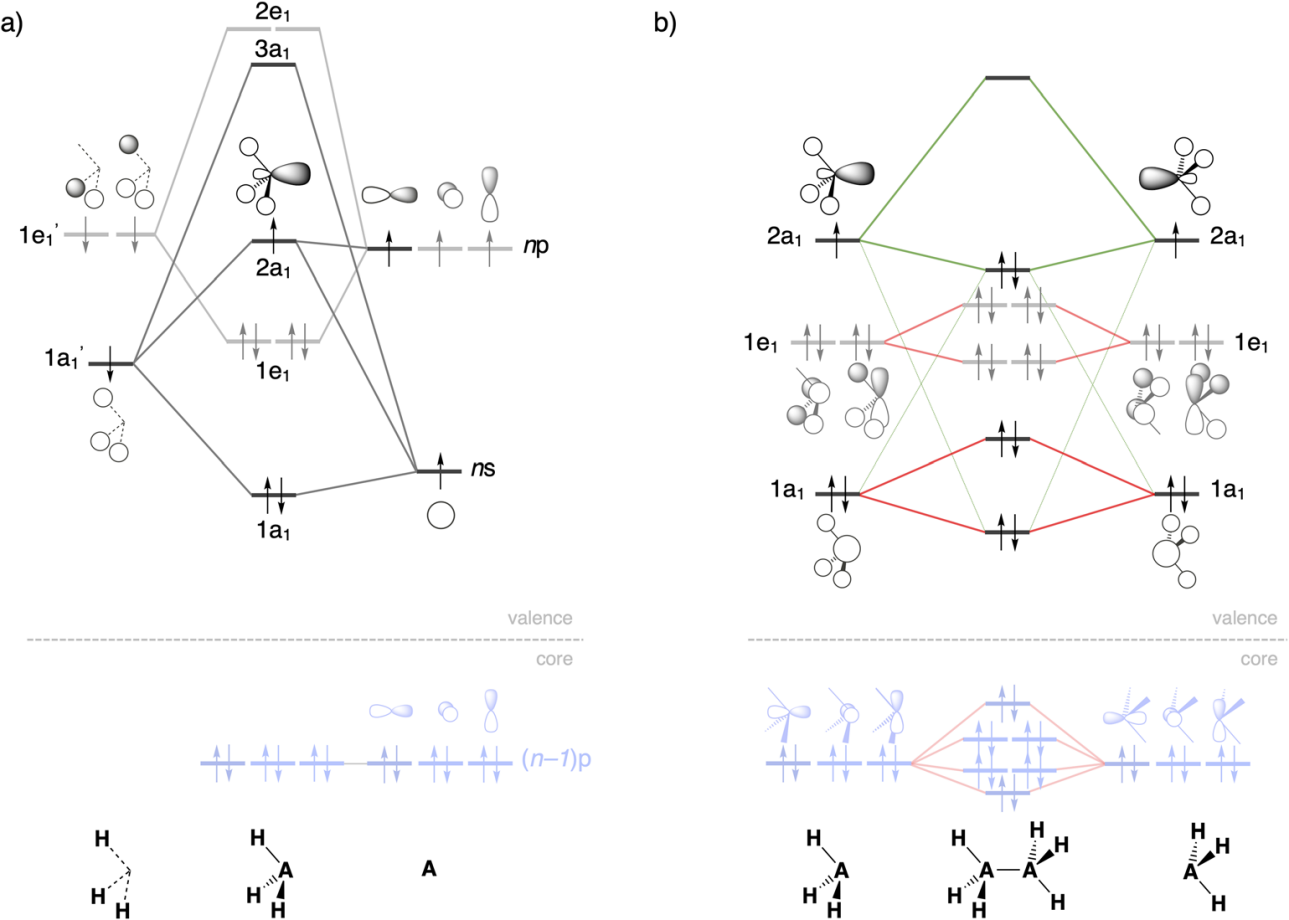
Schematic molecular orbital diagrams of (a) pyramidal H_3_A˙ and (b) H_3_A–AH_3_ (A = C, Si). Core Si 2p orbitals are highlighted in light blue, A–A electron-pair bond and donor–acceptor interactions in green, occupied–occupied orbital repulsion in red. MO numbering starts at the lowest-energy valence AO combination.

As can be seen in Fig. 2, the trends in the interaction energy Δ*E*_int_ determine the trends in bond energy Δ*E* and cause the Si–Si bond to be weaker than the C–C bond. Therefore, we further analyze the bonding mechanism and the interaction energy Δ*E*_int_ using quantitative Kohn–Sham MO theory^15^ and a matching canonical energy decomposition analysis (EDA),^16^ which dissects Δ*E*_int_ into the electrostatic interactions Δ*V*_elstat_, Pauli repulsion Δ*E*_Pauli_, and orbital interactions Δ*E*_oi_, among others (eqn (4); see Computational methods for a theoretical overview). Our quantitative MO and EDA analyses reveal a key role for the Pauli repulsion Δ*E*_Pauli_ behind the weaker Si–Si than C–C bond. In both cases, the main factor preventing the two atoms from coming closer than the equilibrium distance is Pauli repulsion between occupied closed-shell orbitals. The spatially more extended valence AOs of Si lead to the occurrence of a larger occupied–occupied orbital overlap at a longer A–A distance than in the case of the more compact valence AOs of C (*S*_occupied_, see Fig. 2). In addition, Si has a larger number of closed-shell subvalence orbitals (Fig. 3). This situation gives rise to more Δ*E*_Pauli_ for Si–Si than for C–C, which pushes the Si–Si bond to a longer equilibrium distance, where all energy terms are weaker. Note that Δ*E*_Pauli_ is partially absorbed into the destabilizing strain energy Δ*E*_strain_.^35^ The H_3_A˙ radicals pyramidalize to reduce the build-up of steric Pauli repulsion between the substituents as the A–A distance becomes shorter (see Fig. S2 and Table S5†). The Δ*E*_strain_ is always less destabilizing for Si1 than C1 because H_3_Si˙ is already pyramidal in its equilibrium geometry, while H_3_C˙ needs to pyramidalize from its planar equilibrium geometry upon formation of the C–C bond.^36^

Both electrostatic and orbital interactions (Δ*V*_elstat_ and Δ*E*_oi_, respectively) are more stabilizing for Si1 than for C1, thus counteracting, but not overruling, the trends set by Δ*E*_Pauli_. The Si atom has a large nuclear charge and electron cloud, which leads to a stronger electrostatic attraction between the electrons of one H_3_Si˙ fragment with the nuclei of the other H_3_Si˙ fragment than between two H_3_C˙ fragments, at any given bond distance.^37^ Furthermore, the spatially more extended valence AOs of Si lead to an earlier buildup of SOMO–SOMO bond overlap (*S*_bond_) as the two fragments are approaching (see Fig. 2 and 3), although the cancelation of overlap also begins earlier. This situation results in a more stabilizing Δ*E*_oi_ for Si1 than C1 at longer A–A distances. The stabilization of Δ*E*_oi_ upon shortening the A–A distance below the optimum electron-pair bond overlap *S*_bond_ is due to other donor–acceptor orbital interactions (see Fig. S3†). The earlier onset of cancellation effects on the Si–Si bond overlap causes a shallower slope in the Δ*E*_oi_ curve of the Si–Si bond, which therefore comes closer to, and crosses, the Δ*E*_oi_ curve of the C–C bond which remains steeper at these short distances.

**Fig. 4:**
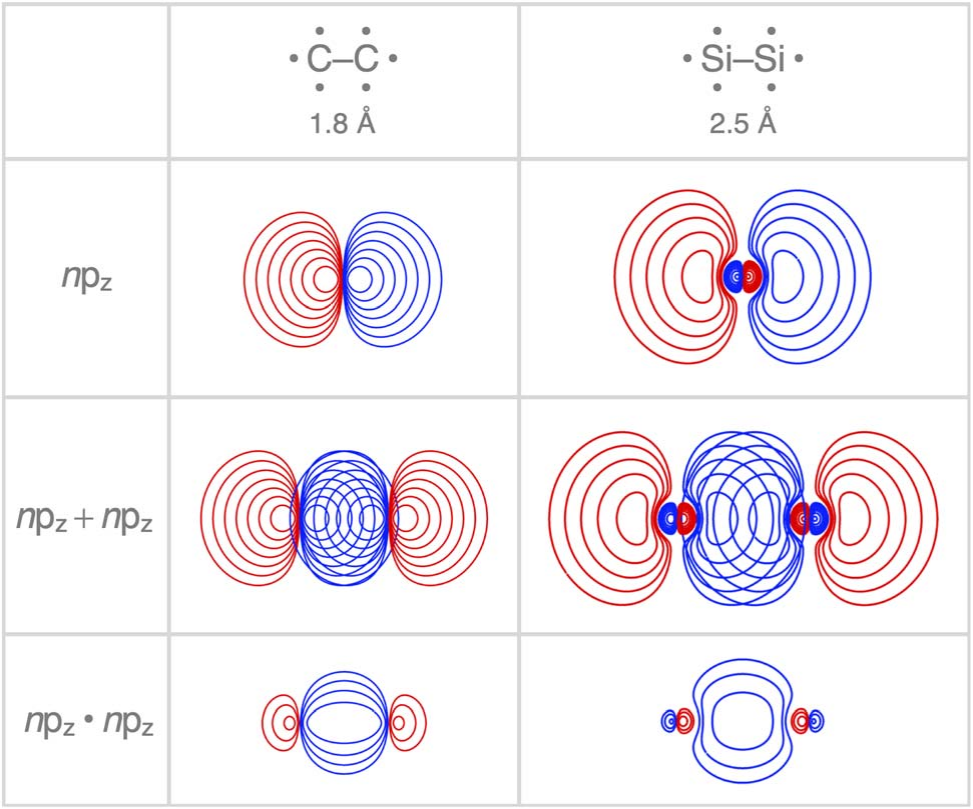
Contour plots (10 contour lines between 0.03, 1.0 for *n*p_z_ and *n*p_z_ + *n*p_z_, and between 0.003, 1.0 for *n*p_z_·*n*p_z_; color represents phase) of the carbon and silicon *n*p_z_ atomic orbitals, their maximum overlap, and respective *n*p_z_·*n*p_z_ overlap density in A–A (A = C, Si). Atoms in their sp^3^ atomic configuration, computed at BLYP-D3(BJ)/TZ2P.

Interestingly, the maximum value of the *S*_bond_ overlap is also larger for Si than C (*S*_bond_ = 0.48 and 0.42, respectively). This effect can be traced back to the 〈*n*p_z_|*n*p_z_〉 overlap between the bare A–A atoms (A = C, Si; Fig. 4). Note that, at the A–A distance with maximum 〈*n*p_z_|*n*p_z_〉 (*i.e.*, 1.8 Å and 2.5 Å for C–C and Si–Si, respectively), the C 2p_z_ orbital crosses the nodal surface and begins to enter into an out-of-phase admixture with the rear lobe of the other C 2p_z_ orbital (see red counter lines of the *n*p_z_·*n*p_z_ overlap densities in Fig. 4). This cancellation of overlap does not occur to the same extent for Si as the radial node of the Si 3p_z_ orbital (nonexistent in C 2p_z_) pushes the region of maximum amplitude of the 3p_z_ lobe further away from the Si nucleus. This circumstance delays the out-of-phase overlap with the backside lobe of the 3p_z_ AO of the other Si atom, resulting in a larger maximum 〈*n*p_z_|*n*p_z_〉 overlap and, therefore, a larger *S*_bond_ for the Si–Si bond than for the C–C bond. The trend from the C–C to the Si–Si bond continues for the heavier group-14 elements with increasingly larger maximum bond overlaps 〈*n*p_z_|*n*p_z_〉 at increasingly longer A–A distances going down group 14 from A = C and Si to Ge, Sn, and Pb (see Fig. S4 and S5†). The largest increase in maximum bond-overlap values, however, occurs in the step from period 2 to period 3 where, for the first time along the series, a p-core shell is introduced. Finally, note that the maximum *S*_bond_ occurs at a H_3_A–AH_3_ bond distance (A = C, Si) slightly shorter than the corresponding equilibrium bond length (Fig. 2). Again, it is the increasing Pauli repulsion at shorter distances that pushes the equilibrium bond lengths to a longer H_3_A–AH_3_ distance. Altogether, our findings once more highlight the well-known role of the Pauli repulsive orbital interactions in determining the length and strength of main-group element bonds.^34,37*b*,38^

### Effect of steric hindrance

Now that we understand the difference in intrinsic R_3_C–CR_3_ and R_3_Si–SiR_3_ (R_3_ = H_3_) bonding mechanism, we evaluate the effect of changing the steric size of the R groups. All trends change systematically on going from R_3_ = H_3_ to *t*-Bu_3_. Hence, we focus our discussion mainly on R_3_ = H_3_ and Ph_3_ to pinpoint the origin of the opposite trends, that is, of decreasing C–C bond strength going from C1 to C5 and increasing Si–Si bond strength going from Si1 to Si5. As will become clear in the following, these opposite trends arise from the net steric interactions that are destabilizing for C (*i.e.*, steric repulsion) and stabilizing for Si (*i.e.*, steric attraction) because of the different A–R bond lengths (A = C, Si) and, consequently, R⋯R distances. Thus, if the R groups are in closer proximity, as in the C systems, due to the shorter C–C and C–R bond lengths, the interaction energy is dominated by the steric Pauli repulsion term, which causes a weakening of the C–C bond as R increases in size. On the other hand, when the R groups are farther apart from each other, as in the Si systems, due to the intrinsically longer Si–Si and Si–R bonds, the steric repulsion is smaller and becomes dominated by steric attraction, resulting in an overall more stabilizing interaction energy and, therefore, Si–Si bond strengthening from R_3_ = H_3_ to Ph_3_. Our findings support and extend earlier reports in the literature that discuss the interplay of repulsive and attractive steric effects in determining the length and strength of C–C bonds and other pheonomena.^8,30^

**Fig. 5:**
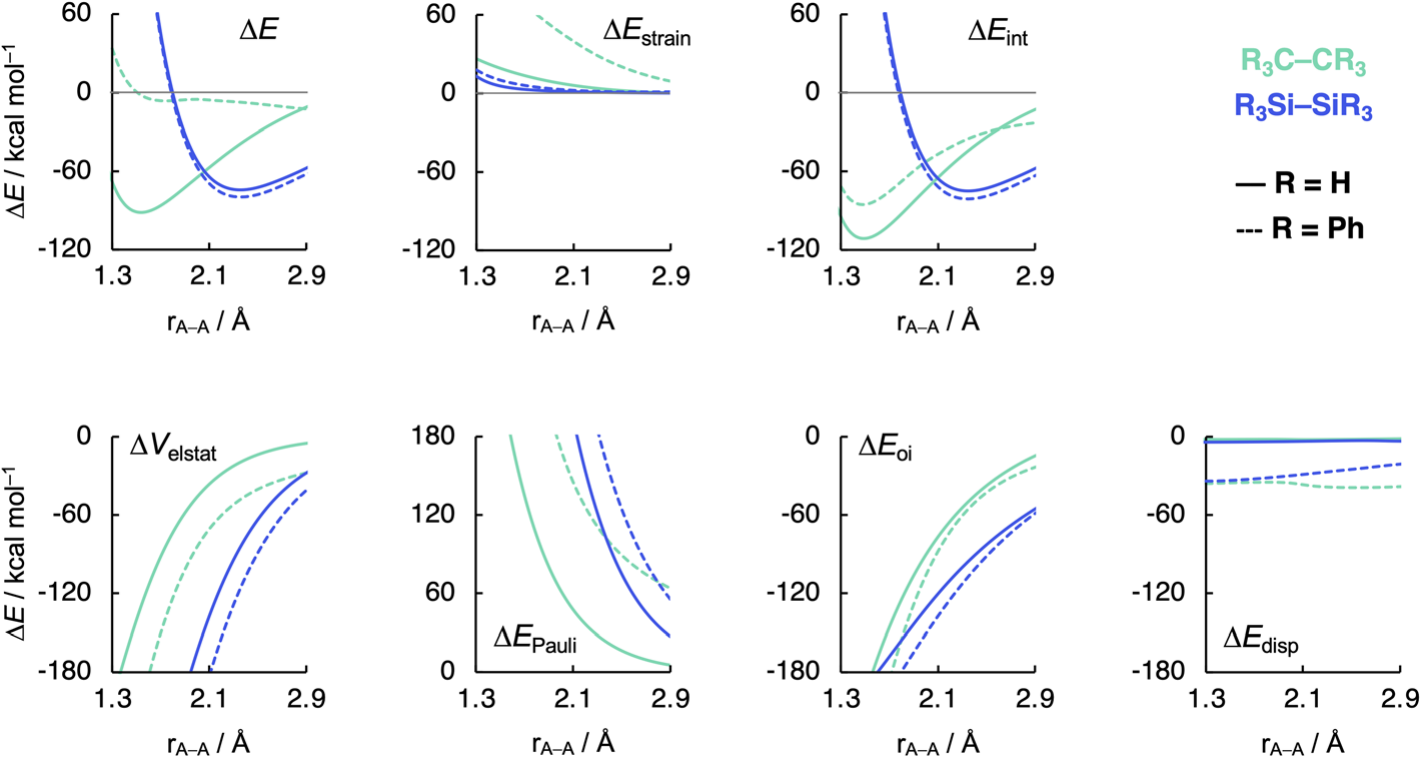
Activation strain model (top row) and energy decomposition analysis (bottom row) as a function of the A–A distance in R_3_A–AR_3_ (A = C, Si; R_3_ = H_3_, Ph_3_) computed at BLYP-D3(BJ)/TZ2P.

**Fig. 6:**
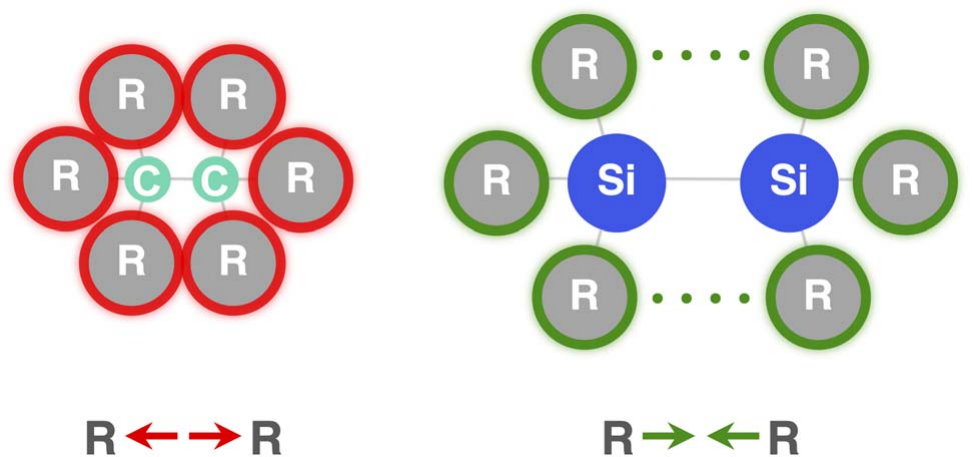
The C–C bond is short and easily weakened by steric Pauli repulsion between bulkier substituents. The Si–Si bond is long and at first benefits from steric dispersion attraction between bulkier substituents.

The ASM and EDA terms as a function of the A–A distance for the R_3_A–AR_3_ systems (A = C, Si; R_3_ = H_3_, Ph_3_) are given in Fig. 5 (see Fig. S6–S8† for the complete data set). As can be seen in Fig. 5, the opposite trends, for R_3_Si–SiR_3_ compared to R_3_C–CR_3_, in bond strength Δ*E* going from R_3_ = H_3_ to Ph_3_ originates mainly from the interaction energy Δ*E*_int_. That is, from R_3_ = H_3_ to Ph_3_, Δ*E*_int_ becomes less stabilizing for C–C (*i.e.*, from full to dashed green lines) and more stabilizing for Si–Si (*i.e.*, from full to dashed blue lines). The substitution of the H atoms by Ph groups in R_3_A–AR_3_ results in an increase in steric Pauli repulsion between the R_3_A˙ fragments. As R increases in size, the number of occupied–occupied orbital overlaps also increases, resulting in a larger Δ*E*_Pauli_. As mentioned before, part of Δ*E*_Pauli_ is absorbed into the strain energy Δ*E*_strain_ as the R_3_A˙ fragments deform in response to the increasing steric repulsion (Fig. 5; see also Fig. S2 and Table S5†). In R_3_C–CR_3_, the R groups are in closer proximity due to the short C–R bonds and, therefore, this increase in steric repulsion is large enough to cause a weakening of the C–C bond going from C1 to C5 (Fig. 6). This effect is much less pronounced in R_3_Si–SiR_3_ simply because the R groups are farther removed from each other compared to R_3_C–CR_3_ (the Si–R bonds are longer than the C–R bonds; Fig. 6). If the C–C bond is artificially placed in the Si–Si geometry and *vice versa*, we observe that the trends in the interaction energy are reversed (Fig. S9†). That is, as R is varied from H to Ph, Δ*E*_int_ becomes more stabilizing for C and less stabilizing for Si.

The much less pronounced increase in Pauli repulsion Δ*E*_Pauli_ going from R_3_ = H_3_ to Ph_3_ in R_3_Si–SiR_3_ allows for the long-range, weakly stabilizing interactions (see Δ*V*_elstat_ and Δ*E*_disp_ in Fig. 5), also referred to as steric attraction,^9^ to take over and strengthen the Si–Si bond from Si1 to Si5. The Δ*V*_elstat_ term is dominated by the nuclear–electron electrostatic attraction,^37^ which becomes more stabilizing as R increases in size. The same occurs for the dispersion energy Δ*E*_disp_ as larger substituent surfaces are in each other's proximity, close enough for dispersion interaction but not yet having large mutual closed-shell overlap. The only exception is the isotropically bulky *tert*-butyl group (*i.e.*, R_3_ = *t*-Bu_3_), whose mutual closed-shell overlap and thus steric Pauli repulsion is large enough to cause a weakening even of the Si–Si bond (Fig. S7†).

### Generalization

**Fig. 7:**
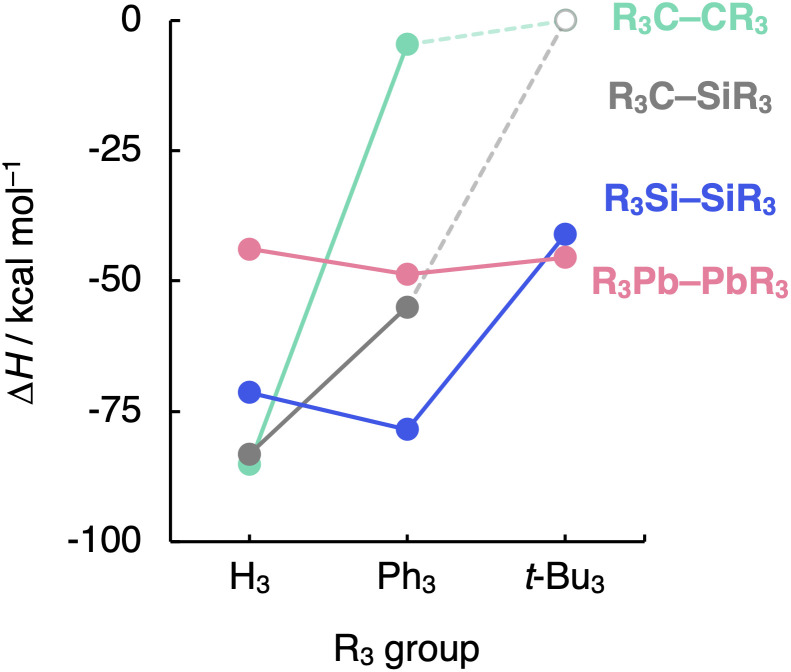
Bond enthalpies (Δ*H*) of the R_3_A–AR_3_ (A = C, Si, Pb; R_3_ = H_3_, Ph_3_, *t*-Bu_3_) systems computed at BLYP-D3(BJ)/TZ2P for A = C, Si and at ZORA-BLYP-D3(BJ)/TZ2P for A = Pb.

Finally, we evaluate the generality of the steric effects observed in the R_3_A–AR_3_ (A = C, Si; R_3_ = H_3_, Me_3_, Me_2_Ph, MePh_2_, Ph_3_, *t*-Bu_3_) model systems by extending our bonding analysis to mixed R_3_C–SiR_3_ (R_3_ = H_3_, Ph_3_, *t*-Bu_3_) and heavier R_3_A–AR_3_ (A = Ge, Sn, Pb; R_3_ = H_3_, Ph_3_, *t*-Bu_3_) systems. As can be seen in Fig. 7 (the complete dataset is provided in Table S6†), the C–Si bond shows behavior in between that of the C–C and the Si–Si bonds while still retaining the C–C bond sensitivity to steric hindrance. The H_3_C–SiH_3_ bond is somewhat weaker and longer (Δ*H* = −83.2 kcal mol^−1^ and *r*_C–Si_ = 1.888 Å) than H_3_C–CH_3_ (Δ*H* = −85.2 kcal mol^−1^ and *r*_C–C_ = 1.538 Å) while stronger and shorter than H_3_Si–SiH_3_ (Δ*H* = −71.4 kcal mol^−1^ and *r*_Si–Si_ = 2.356 Å). This is again due to the increase in mutual steric Pauli repulsion between the radical fragment as one H_3_A˙ is varied from H_3_C˙ to H_3_Si˙ (Fig. S10†).

On the other hand, the sensibility of the R_3_A–AR_3_ bond strength towards the substituents' bulkiness decreases as the central atom increases in size from C–C and Si–Si to Ge–Ge, Sn–Sn, and Pb–Pb (see Table S7† the complete dataset). Note that the R_3_Pb–PbR_3_ bond weakens by *ca.* 3 kcal mol^−1^ as R_3_ is varied from H_3_ to *t*-Bu_3_, while the R_3_Si–SiR_3_ bond weakens by almost 30 kcal mol^−1^ (Fig. 7). This is again due to the increase in the effective atom size of A and, therefore, the longer R⋯R distances. If the R groups are packed together, as in R_3_C–CR_3_, occupied–occupied orbital overlap is significant and the R_3_A–AR_3_ bond strength is dominated by steric repulsion between the substituents. If the R groups are further away from each other, as in R_3_Si–SiR_3_ and heavier analogs, that overlap becomes negligible, and dispersion takes over; thus, steric repulsion turns into steric attraction. But if the A–A and A–R bond becomes too long and, therefore, the R groups are too far removed from each other, both short-range repulsive and long-range attractive steric effects are weakened (see ASM and EDA terms in Fig. S11 and S12†), and the R_3_A–AR_3_ bond is almost insensitive to the size of R. The latter holds in particular for the Pb–Pb bond (see Fig. 7).

## Conclusions

The Si–Si bond is intrinsically longer, weaker, and much less sensitive to substitution of H substituents for bulkier groups than the C–C bond. Thus, whereas the R_3_C–CR_3_ bond significantly weakens as R is varied from H to Me to Ph, the R_3_Si–SiR_3_ bond is somewhat strengthened along the same R series and only weakens when R is the isotropically bulky *t*-Bu group. This follows from our quantum chemical bonding analyses using dispersion-corrected density functional theory.

The H_3_Si–SiH_3_ bond is longer and weaker than the H_3_C–CH_3_ bond because the larger number of occupied shells and the larger spatial extension of silicon's valence AOs cause an earlier onset of, and a stronger, steric Pauli repulsion that destabilizes the Si–Si bond and pushes it to a longer equilibrium distance. This trend and mechanism hold for the entire series of group-14 H_3_A–AH_3_ bonds which become weaker and longer along C–C, Si–Si, Ge–Ge, Sn–Sn, and Pb–Pb. Interestingly, this is so despite the electron-pair bonding overlap and orbital interactions becoming stronger, not weaker, along this series. The reason for the increasing bond overlap is the introduction of a radial node in the valence *n*p_z_ orbital from C to Si, which delays the occurrence of cancellation of bond overlap between the SOMOs, resulting in a larger maximum SOMO–SOMO overlap for Si.

When the hydrogen atoms in H_3_C–CH_3_ are replaced by larger R groups, the C–C bond is weakened due to a steep increase in steric Pauli repulsion between the R groups which is partially converted into strain energy associated with geometrical deformation of the R_3_C moieties in R_3_C–CR_3_. This increase in steric repulsion is less pronounced in R_3_Si–SiR_3_ as the R groups are farther removed from each other due to the longer Si–R bonds. Then, repulsive interactions are compensated by long-range attractive ones in R_3_Si–SiR_3_. Only R = *t*-Bu gives rise to steric repulsion that is large enough to cause a weakening of the R_3_Si–SiR_3_ bond. The sensitivity of the bond strength with respect to the substituents' bulkiness (even for R = *t*-Bu) further decreases going down group-14 due to the increasingly longer A–R bonds. Our findings nicely equip chemists with the rational design principles to tune the strength of A–A and A–A′ bonds at will.

## Data availability

All data of this study are available in the main text and ESI.†

## Author contributions

FMB and JMS conceived the project, which was supervised by TAH and FMB. DRS and EB carried out the quantum chemical computations and bonding analyses. DRS also drafted the manuscript. DRS, TAH, and FMB discussed all results. All authors reviewed the manuscript.

## Conflicts of interest

There are no conflicts of interest to declare.

## Supplementary Material

SC-015-D3SC06215E-s001
