# Supplementary material for: Nature and strength of group-14 A–A′ bonds
Source: Chem Sci. 2024 Jan 16;15(5):1648–56. doi: 10.1039/d3sc06215e (PMC10829027; doi:10.1039/d3sc06215e)
Supplement: SC-015-D3SC06215E-s001 [file SC-015-D3SC06215E-s001.pdf]

Electronic Supplementary Material (ESI) for Chemical Science.
This journal is © The Royal Society of Chemistry 2024

Nature and Strength of Group-14 A–A' Bonds

Daniela Rodrigues Silva,+[a] Eva Blokker,+[a] J. Martijn van der Schuur,[b] Trevor A. Hamlin,[a] and F. Matthias Bickelhaupt*[a,c,d]

[a] Dr. D. Rodrigues Silva, E. Blokker, Dr. T. A. Hamlin, Prof. Dr. F. M. Bickelhaupt
Department of Chemistry and Pharmaceutical Sciences, AIMMS, Vrije Universiteit Amsterdam, De Boelelaan 1108, 1081 HZ Amsterdam, The Netherlands.
E–mail: f.m.bickelhaupt@vu.nl
Web address: https://www.theochem.nl
[b] Dr. Ir. J. M. van der Schuur
Polymer Specialties, Nouryon, Zutphenseweg 10, 7418 AJ Deventer, The Netherlands.
[c] Prof. Dr. F. M. Bickelhaupt
Institute of Molecules and Materials, Radboud University, Heyendaalseweg 135, 6525 AJ Nijmegen, The Netherlands.
[d] Prof. Dr. F. M. Bickelhaupt
Department of Chemical Sciences, University of Johannesburg, Auckland Park, Johannesburg 2006, South Africa.
[+] These authors contributed equally to this work.

Contents

Table S1. Bond enthalpies (ΔH; in kcal mol^{-1}), activation strain analysis (in kcal mol^{-1}), and bond lengths (in Å) of the $R_3A–AR_3$ systems (A = C, Si; R_3 = H_3, Me_3, Me_2Ph, $MePh_2$, Ph_3, *t*-Bu_3).

Figure S1. Activation strain analysis (top row) and energy decomposition analysis (bottom row) as a function of the A–A distance in $R_3A–AR_3$ (A = C, Si; R_3 = H_3, Ph_3) computed at M06-2X/TZ2P.

Table S2. Energy decomposition analysis terms (in kcal mol^{-1}), and bond lengths (in Å) of the $R_3A–AR_3$ bonds (A = C, Si; R_3 = H_3, Me_3, Me_2Ph, $MePh_2$, Ph_3, *t*-Bu_3).

Table S3. Comparison between computed and experimental bond dissociation enthalpies (ΔH_{BDE}; in kcal mol^{-1}) of the $R_3A–AR_3$ bonds (A = C, Si; R_3 = H_3, Me_3, Me_2Ph, $MePh_2$, Ph_3, *t*-Bu_3).

Table S4. ADF total energies (in kcal mol^{-1}), bond lengths (in Å), and imaginary frequencies (in cm^{-1}) for various conformations of the R_3A–AR_3 molecules (A = C, Si; R_3 = H_3, Me_3, Me_2Ph, $MePh_2$, Ph_3, *t*-Bu_3). The most stable conformations are in bold and used in all further analyses.

Figure S2. Strain energy ΔE_{strain}, $\theta_{R\text{–}A\text{–}R}$ angular deformation, and $r_{A\text{–}R}$ bond stretch relative to the isolated radical as a function of the A–A distance in R_3A–AR_3 (A = C, Si; R_3 = H_3, Ph_3), computed at BLYP-D3(BJ)/TZ2P.

Table S5. Strain energy terms (in kcal mol^{-1}) associated with the stepwise deformation of the $R_3A^{\bullet}$ radical into the geometry adopted in the R_3A–AR_3 molecule (where A = C, Si; R_3 = H_3, Ph_3).

Figure S3. Occupancy of the $1a_1$ orbital, $\langle 1a_1|2a_1\rangle$ and $\langle 1a_1|3a_1\rangle$ orbital overlaps and their corresponding energy gaps $\Delta\varepsilon$ (in eV) as a function of the A–A distance in H_3A–AH_3 (**1**, A = C, Si), computed at BLYP-D3(BJ)/TZ2P.

Figure S4. Orbital overlaps as a function of the distance for C_2 (green), Si_2 (blue), Ge_2 (pink), and Sn_2 (grey), in which the atoms are in their sp^3 atomic configuration, computed at BLYP-D3(BJ)/TZ2P for A = C, Si and at ZORA-BLYP-D3(BJ)/TZ2P for A = Ge, Sn, Pb.

Figure S5. Contour plots (10 contour lines between 0.03, 1.0; scan values are evenly spaced; color represents phase) of the C, Si, Ge, and Sn np_z atomic orbitals (top), their maximum $\langle np_z|np_z\rangle$ overlap (middle), and respective np_znp_z overlap density (bottom) in A–A (A = C, Si, Ge, Sn). Atoms in their sp^3 atomic configuration, computed at BLYP-D3(BJ)/TZ2P for A = C, Si and at ZORA-BLYP-D3(BJ)/TZ2P for A = Ge, Sn, Pb.

Figure S6. Activation strain model (top) and energy decomposition analysis (bottom) as a function of the C–C distance in R_3C–CR_3 (R_3 = H_3, Me_3, Me_2Ph, $MePh_2$, Ph_3), computed at BLYP-D3(BJ)/TZ2P.

Figure S7. Activation strain model (top) and energy decomposition analysis (bottom) as a function of the Si–Si distance in R_3Si–SiR_3 (R_3 = H_3, Me_3, Me_2Ph, $MePh_2$, Ph_3, *t*-Bu_3), computed at BLYP-D3(BJ)/TZ2P.

Figure S8. SOMO–SOMO overlap as a function of the A–A distance in R_3A–AR_3 (A = C, Si; R_3 = H_3, Me_3, Me_2Ph, $MePh_2$, Ph_3, *t*-Bu_3), computed at BLYP-D3(BJ)/TZ2P.

Figure S9. a) Numerical experiment in which the Si–Si bond in $R_3A–AR_3$ (A = C, Si; R_3 = H_3, Ph_3) is replaced by the C–C bond, and vice versa, keeping the other geometrical parameters unchanged; and b) energy decomposition analysis as a function of the A–A distance in the constrained $R_3A–AR_3$ geometry (A = C, Si; R_3 = H_3, Ph_3) of the above numerical experiment, computed at BLYP-D3(BJ)/TZ2P.

Table S6. Bond enthalpies (ΔH; in kcal mol^{-1}) of the mixed $R_3A–AR_3$ systems (A = C, Si; R_3 = H_3, Ph_3, *t*-Bu_3).

Figure S10. Activation strain model and energy decomposition analysis terms as a function of the A–A distance in the mixed $H_3A–AH_3$ system (A = C, Si). The dispersion energy ΔE_{disp} is nearly constant and, therefore, not shown, computed at BLYP-D3(BJ)/TZ2P.

Table S7. Bond enthalpies (ΔH; in kcal mol^{-1}) of the $R_3A–AR_3$ systems (A = C, Si, Ge, Sn, Pb; R_3 = H_3, Ph_3, *t*-Bu_3).

Figure S11. Activation strain model (top) and energy decomposition analysis (bottom) as a function of the Pb–Pb distance in $R_3Pb–PbR_3$ (R_3 = H_3, Ph_3, *t*-Bu_3), computed at ZORA-BLYP-D3(BJ)/TZ2P.

Figure S12. Activation strain model (top row) and energy decomposition analysis (bottom row) as a function of the A–A distance in $R_3A–AR_3$ (A = C, Si, Pb; R_3 = H_3, Ph_3), computed at BLYP-D3(BJ)/TZ2P for A = C, Si and at ZORA-BLYP-D3(BJ)/TZ2P for A = Pb.

Table S8. Cartesian coordinates (in Å), energies (electronic *E* and enthalpy *H*, in kcal mol^{-1}), number of imaginary frequencies (N_{imag}), and total spin number (*S*) of the equilibrium geometries of all $R_3A–AR_3$ systems studied herein, computed at BLYP-D3(BJ)/TZ2P for A = C, Si and at ZORA-BLYP-D3(BJ)/TZ2P for A = Ge, Sn, Pb.

Table S9. Cartesian coordinates (in Å), energies (electronic *E* and enthalpy *H*, in kcal mol^{-1}), number of imaginary frequencies (N_{imag}), and total spin number (*S*) of the equilibrium geometries of the $R_3A^{\bullet}$ radical species studied herein, computed at (U)BLYP-D3(BJ)/TZ2P for A = C, Si and at (U)ZORA-BLYP-D3(BJ)/TZ2P for A = Ge, Sn, Pb.

Table S10. Cartesian coordinates (in Å), energies (electronic *E* and enthalpy *H*, in kcal mol^{-1}), number of imaginary frequencies (N_{imag}), and total spin number (*S*) of the equilibrium geometries of the $R_3A–AR_3$ systems studied herein, computed at M06-2X/TZ2P.

Table S11. Cartesian coordinates (in Å), energies (electronic E and enthalpy H, in kcal mol^{-1}), number of imaginary frequencies (N_{imag}), and total spin number (S) of the equilibrium geometries of the $R_3A^{\bullet}$ radical species studied herein, computed at (U)M06-2X/TZ2P.

Table S1. Bond enthalpies (ΔH; in kcal mol^{-1}), activation strain analysis (in kcal mol^{-1}), and bond lengths (in Å) of the $R_3A–AR_3$ systems (A = C, Si; R_3 = H_3, Me_3, Me_2Ph, $MePh_2$, Ph_3, t-Bu_3).[a]

No.	Species	ΔH	ΔE	ΔE_{strain}	ΔE_{int}	$r_{A–A}$
**C1**	$H_3C–CH_3$	–90.0	–97.4	16.4	–113.8	1.525
**C2**	$Me_3C–CMe_3$	–76.8	–81.8	21.6	–103.4	1.571
**C3**[b]	$PhMe_2C–CMe_2Ph$	–55.3	–59.3	37.6	–96.9	1.596
**C4**[c]	$Ph_2MeC–CMePh_2$	–37.7[d]	–41.9	53.9	–95.7	1.610
**C5**	$Ph_3C–CPh_3$	–9.7[d]	–13.0	68.2	–81.1	1.696
**Si1**	$H_3Si–SiH_3$	–71.5	–74.4	0.5	–74.9	2.337
**Si2**	$Me_3Si–SiMe_3$	–74.8	–76.0	0.2	–76.2	2.343
**Si3**[b]	$PhMe_2Si–SiMe_2Ph$	–73.6	–74.7	0.7	–76.1	2.345
**Si4**[c]	$Ph_2MeSi–SiMePh_2$	–74.7[d]	–75.2	2.6	–77.8	2.345
**Si5**	$Ph_3Si–SiPh_3$	–76.9[d]	–77.0	1.0	–77.9	2.355
**Si6**	t-$Bu_3Si–Sit$-Bu_3	[e]	–40.2	20.3	–60.5	2.675

[a] Computed at M06-2X/TZ2P at 298.15 K and 1 atm. All structures are staggered. [b] *Gauche* conformation. [c] *Anti* conformation. [d] Numerical accuracy of the frequency calculations was set to GOOD due to technical reasons. [e] Frequency calculations could not be completed due to technical reasons.

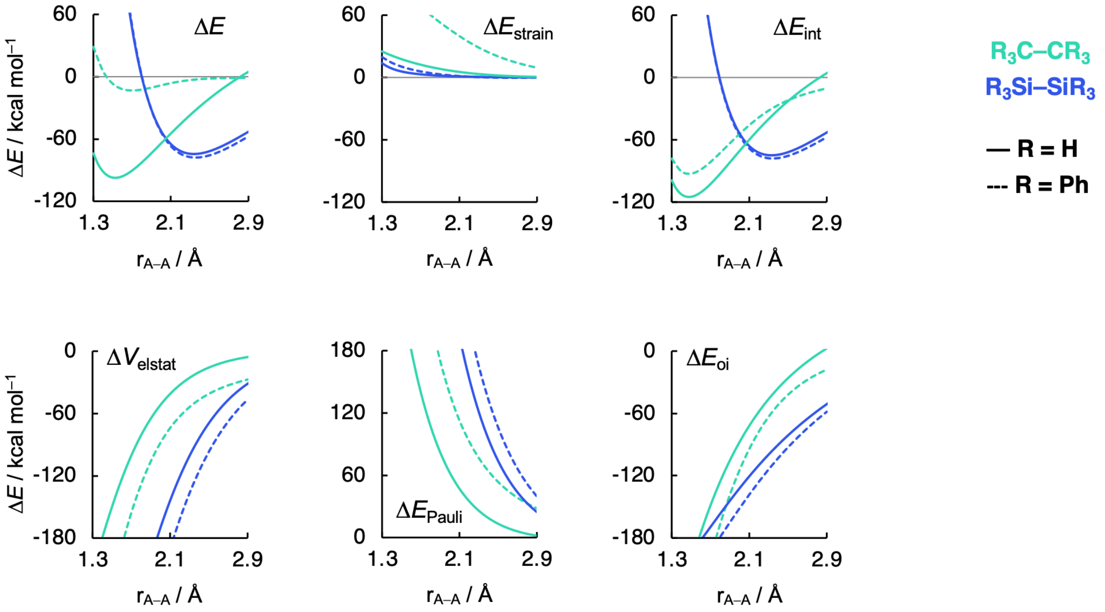

Figure S1. Activation strain analysis (top row) and energy decomposition analysis (bottom row) as a function of the A–A distance in $R_3A–AR_3$ (A = C, Si; R_3 = H_3, Ph_3) computed at M06-2X/TZ2P.

Table S2. Energy decomposition analysis terms (in kcal mol^{-1}), and bond lengths (in Å) of the $R_3A–AR_3$ bonds (A = C, Si; R_3 = H_3, Me_3, Me_2Ph, $MePh_2$, Ph_3, t-Bu_3).[a]

No.	Species[b]	ΔE_{int}	ΔV_{elstat}	ΔE_{Pauli}	ΔE_{oi}	ΔE_{disp}	$\Delta E_{spinpol}$	$r_{A–A}$
**C1**	$H_3C–CH_3$	–110.4	–129.5	204.6	–186.4	–2.0	2.9	1.538
**C2**	$Me_3C–CMe_3$	–94.6	–161.1	259.0	–184.6	–11.3	3.4	1.597
**C3**[c]	$PhMe_2C–CMe_2Ph$	–86.5	–152.6	270.2	–189.7	–18.3	3.9	1.627
**C4**[d]	$Ph_2MeC–CMePh_2$	–86.8	–158.2	285.0	–193.0	–24.5	3.9	1.638
**C5**	$Ph_3C–CPh_3$	–72.2	–139.0	272.0	–173.8	–34.8	3.4	1.738
**Si1**	$H_3Si–SiH_3$	–74.8	–84.5	106.1	–94.9	–3.1	1.6	2.356
**Si2**	$Me_3Si–SiMe_3$	–75.4	–123.5	147.2	–93.1	–7.9	1.9	2.357
**Si3**[c]	$PhMe_2Si–SiMe_2Ph$	–75.4	–118.3	153.2	–99.0	–13.3	2.0	2.356
**Si4**[d]	$Ph_2MeSi–SiMePh_2$	–78.8	–120.8	158.4	–100.8	–17.7	2.1	2.353
**Si5**	$Ph_3Si–SiPh_3$	–81.0	–115.9	165.1	–106.2	–26.2	2.2	2.358
**Si6**	t-$Bu_3Si–Sit$-Bu_3	–60.8	–72.7	112.5	–78.4	–24.1	1.9	2.726

[a] Computed at BLYP-D3(BJ)/TZ2P. All structures are staggered. [b] **C6** does not form a stable C–C electron-pair bond. [c] *Gauche* conformation. [d] *Anti* conformation.

Table S3. Comparison between computed and experimental bond dissociation enthalpies (ΔH_{BDE}; in kcal mol^{-1}) of the $R_3A–AR_3$ bonds (A = C, Si; R_3 = H_3, Me_3, Me_2Ph, $MePh_2$, Ph_3, t-Bu_3).

				Experimental ΔH_{BDE}				
No.	Species[a]	**R3**	ΔH_{BDE}[b]	Ref.3[c]	Ref.12a[d]	Ref.12b[d]	Ref.29a[c]	Ref.29b[c]
**C1**	$H_3C–CH_3$	**1**	85.2	87			85–87	88[b]
**C2**	$Me_3C–CMe_3$	**2**	64.0	67.4				70[b]
**C3**	$PhMe_2C–CMe_2Ph$	**3**	42.1	45.7				
**C4**	$Ph_2MeC–CMePh_2$	**4**	25.9					
**C5**	$Ph_3C–CPh_3$	**5**	4.6					
**Si1**	$H_3Si–SiH_3$	**1**	71.4					74[c]
**Si2**	$Me_3Si–SiMe_3$	**2**	73.0			67 ± 2		80.5[b]
**Si3**	$PhMe_2Si–SiMe_2Ph$	**3**	73.8			65.3 ± 6		
**Si4**	$Ph_2MeSi–SiMePh_2$	**4**	74.4			78.7 ± 20		
**Si5**	$Ph_3Si–SiPh_3$	**5**	78.5		88	90.4 ± 6		
**SI6**	t-$Bu_3Si–Sit$-Bu_3	**6**	41.0					

[a] **C6** does not form a stable C–C electron-pair bond. [b] Computed at BLYP-D3(BJ)/TZ2P at 298.15 K and 1 atm. All structures are staggered. [c] Bond dissociation estimated from the rates of thermal decomposition. [d] Bond dissociation calculated from the heats of formation of the radicals and molecules.

Table S4. ADF total energies (in kcal mol^{-1}), bond lengths (in Å), and imaginary frequencies (in cm^{-1}) for various conformations of the $R_3A–AR_3$ molecules (A = C, Si; R_3 = H_3, Me_3, Me_2Ph, $MePh_2$, Ph_3, *t*-Bu_3). The most stable conformations are in bold and used in all further analyses.[a]

	$E_{complex}$	$r_{A–A}$	Conformation		Imaginary freq.
**C1**	**–900.79**	**1.538**	**staggered**		
	–898.26	1.553	eclipsed		–289 cm^{-1}
**C2**	**–3096.32**	**1.597**	**staggered**		
**C3**	–5393.35	1.626	staggered	*anti*	
	**–5394.08**	**1.627**	**staggered**	***gauche***	
**C4**	**–7685.67**	**1.638**	**staggered**	***anti***	
	–7684.87	1.672	staggered	*gauche*	
**C5**	**–9975.43**	**1.738**	**staggered**		
	–9974.01	1.754	eclipsed		
	**–9984.04**	**3.480**	**staggered**	***vdW***	
	–9982.92	3.403	Eclipsed	*vdW*	
**C6**	–9551.26	5.105	staggered	*singlet*	
	**–9569.14**	**5.285**	**staggered**	***triplet***	

	$E_{complex}$	d(A–A)	Conformation		Imaginary freq.
**Si1**	**–690.70**	**2.356**	**staggered**		
	–689.72	2.369	eclipsed		–131 cm^{-1}
**Si2**	**–2947.16**	**2.357**	**staggered**		
**Si3**	–5246.85	2.353	staggered	*anti*	
	**–5247.77**	**2.356**	**staggered**	***gauche***	
**Si4**	**–7547.79**	**2.353**	**staggered**	***anti***	
	–7547.76	2.362	staggered	*gauche*	
**Si5**	**–9853.43**	**2.358**	**staggered**		
	–9850.14	2.386	eclipsed		
	no minimum		staggered	*vdW*	
	no minimum		eclipsed	*vdW*	
**Si6**	**–9484.26**	**2.726**	**staggered**		

[a] Computed at BLYP-D3(BJ)/TZ2P.

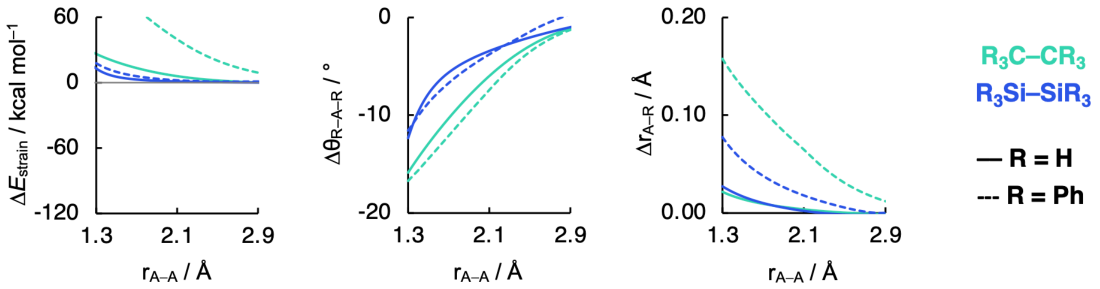

Figure S2. Strain energy ΔE_{strain}, $\theta_{\text{R–A–R}}$ angular deformation, and $r_{\text{A–R}}$ bond stretch relative to the isolated radical as a function of the A–A distance in $R_3A–AR_3$ (A = C, Si; R_3 = H_3, Ph_3), computed at BLYP-D3(BJ)/TZ2P.

Table S5. Strain energy terms (in kcal mol^{-1}) associated with the stepwise deformation of the $R_3A^{•}$ radical[a] into the geometry adopted in the $R_3A–AR_3$ molecule[b] (where A = C, Si; R_3 = H_3, Ph_3).[c]

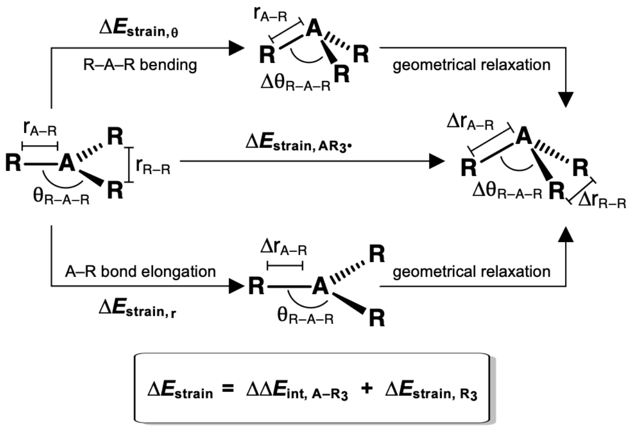

A	R_3	$\Delta E_{\text{strain,AR}_3^{•}}$	$\Delta\Delta E_{\text{int,A–R}_3}$	$\Delta E_{\text{strain,R}_3}$	$\Delta E_{\text{strain},\theta}$	$\Delta\Delta E_{\text{int,A–R}_3}$	$\Delta E_{\text{strain,R}_3}$	$\Delta E_{\text{strain,r}}$	$\Delta\Delta E_{\text{int,A–R}_3}$	$\Delta E_{\text{strain,R}_3}$
**C**	**H_3**	4.1	0.6	3.5	4.1	0.1	4.0	0.0	0.5	–0.5
	**Ph_3**	23.3	19.7	3.6	22.4	9.1	13.3	5.9	15.0	–9.1
**Si**	**H_3**	0.7	–0.1	0.8	0.7	–0.2	0.9	0.0	0.1	–0.1
	**Ph_3**	1.4	1.8	–0.4	1.4	–0.3	1.7	0.3	0.8	–0.5

[a] $R_3C^{•}$ has a planar equilibrium geometry whereas $R_3Si^{•}$ is pyramidal. [b] Analysis at a consistent geometry with an $R_3A–AR_3$ bond distance of 2.0 Å. [c] Computed at BLYP-D3(BJ)/TZ2P.

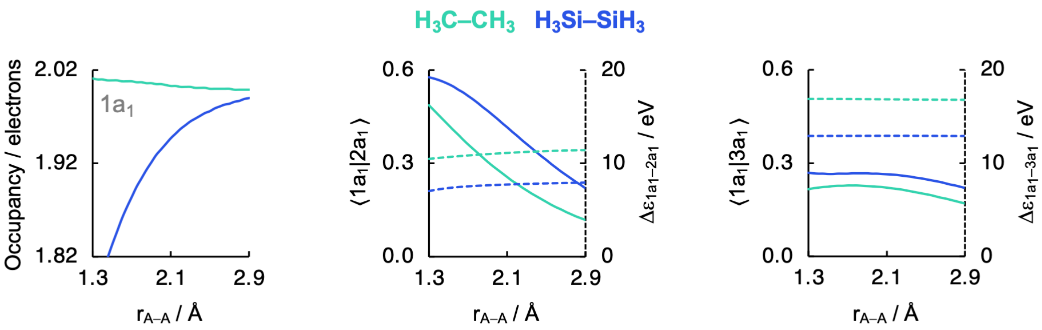

Figure S3. Occupancy of the $1a_1$ orbital, $\langle 1a_1|2a_1\rangle$ and $\langle 1a_1|3a_1\rangle$ orbital overlaps and their corresponding energy gaps $\Delta\varepsilon$ (in eV) as a function of the A–A distance in $H_3A–AH_3$ (**1**, A = C, Si), computed at BLYP-D3(BJ)/TZ2P.

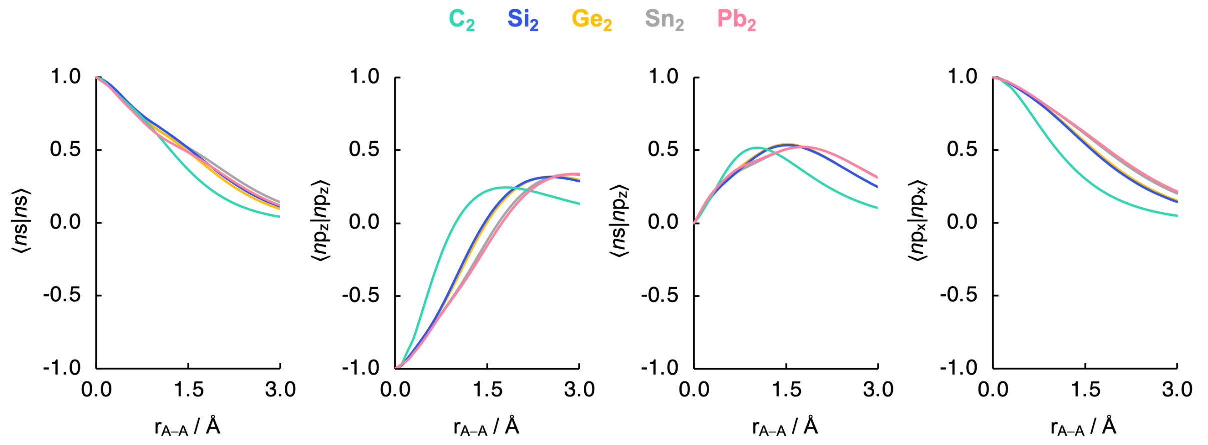

Figure S4. Orbital overlaps as a function of the distance for C_2 (green), Si_2 (blue), Ge_2 (pink), and Sn_2 (grey), in which the atoms are in their sp^3 atomic configuration, computed at BLYP-D3(BJ)/TZ2P for A = C, Si and at ZORA-BLYP-D3(BJ)/TZ2P for A = Ge, Sn, Pb.

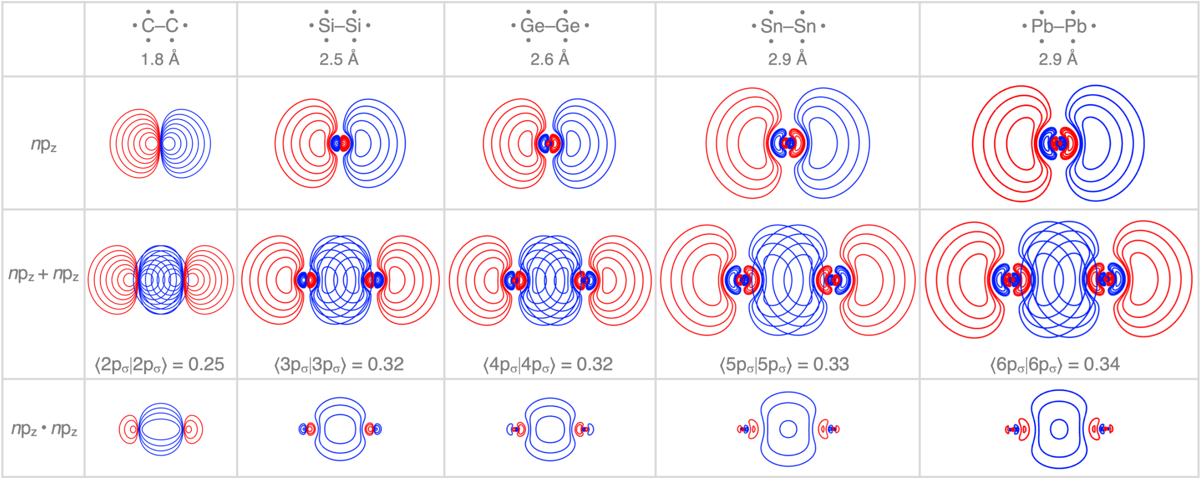

Figure S5. Contour plots (10 contour lines between 0.03, 1.0 for np_z and $np_z + np_z$, and between 0.003, 1.0 for np_z•np_z; color represents phase) of the C, Si, Ge, Sn, and Pb np_z atomic orbitals (top), their maximum overlap (middle), and respective np_z•np_z overlap density (bottom) in A–A (A = C, Si, Ge, Sn). Atoms in their sp^3 atomic configuration, computed at BLYP-D3(BJ)/TZ2P for A = C, Si and at ZORA-BLYP-D3(BJ)/TZ2P for A = Ge, Sn, Pb.

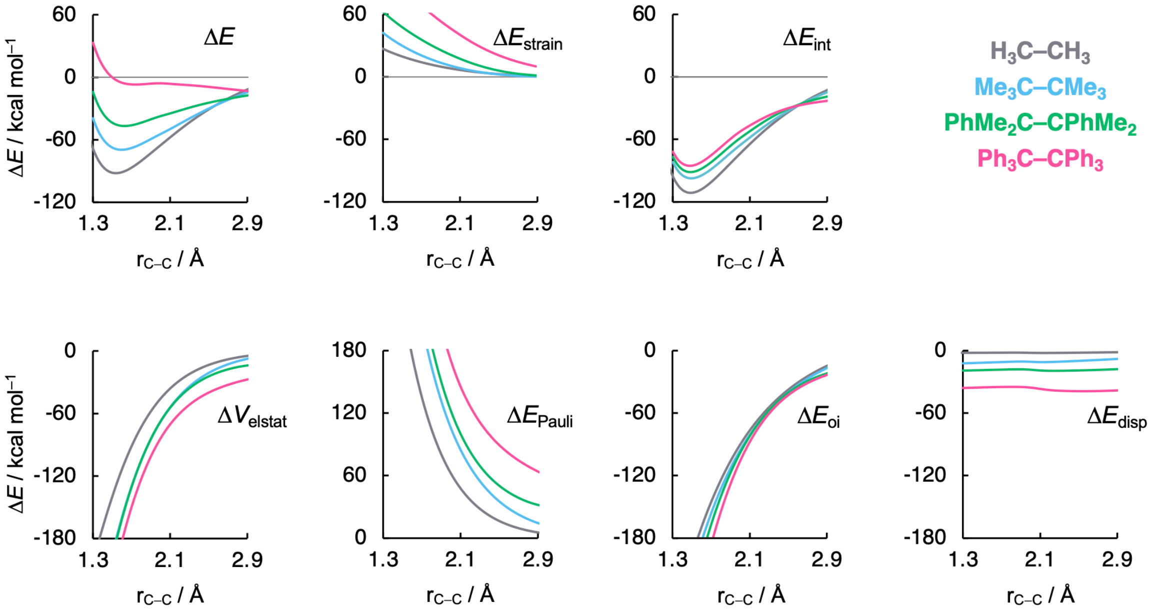

Figure S6. Activation strain model (top) and energy decomposition analysis (bottom) as a function of the C–C distance in $R_3C–CR_3$ (R_3 = H_3, Me_3, Me_2Ph, $MePh_2$, Ph_3), computed at BLYP-D3(BJ)/TZ2P.

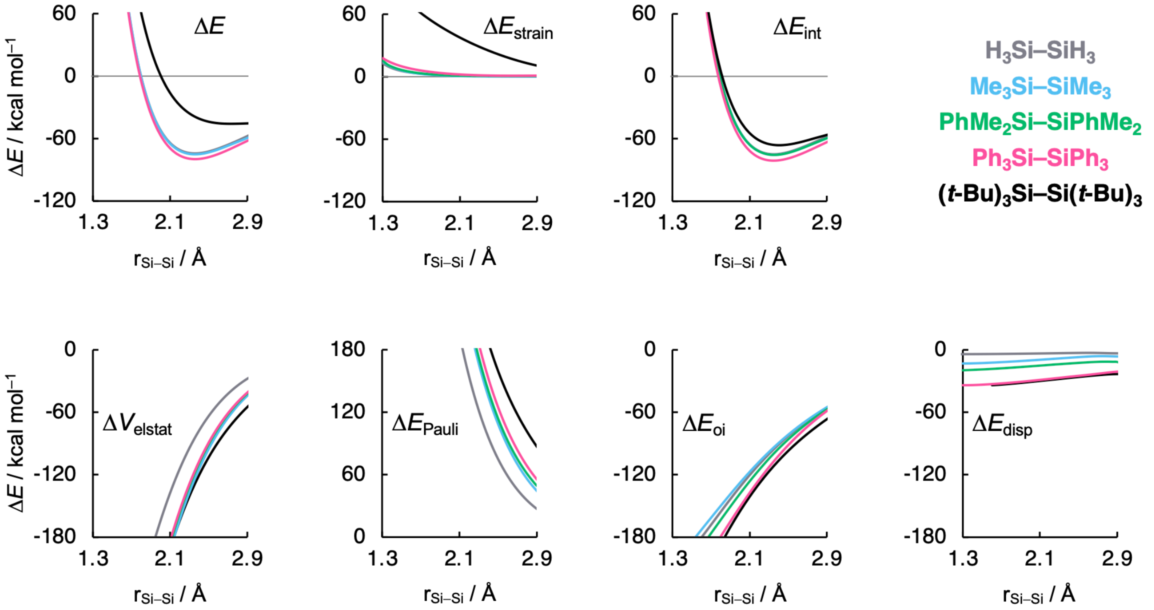

Figure S7. Activation strain model (top) and energy decomposition analysis (bottom) as a function of the Si–Si distance in $R_3Si–SiR_3$ (R_3 = H_3, Me_3, Me_2Ph, $MePh_2$, Ph_3, *t*-Bu_3), computed at BLYP-D3(BJ)/TZ2P.

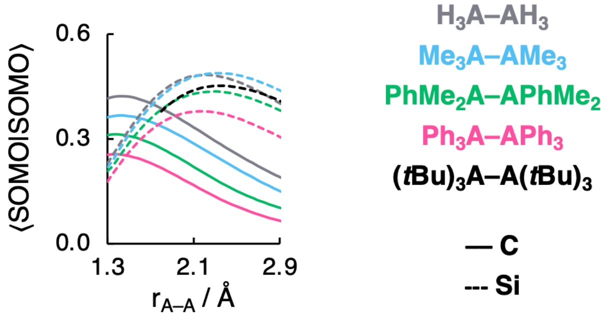

Figure S8. SOMO–SOMO overlap as a function of the A–A distance in $R_3A–AR_3$ (A = C, Si; R_3 = H_3, Me_3, Me_2Ph, $MePh_2$, Ph_3, *t*-Bu_3), computed at BLYP-D3(BJ)/TZ2P.

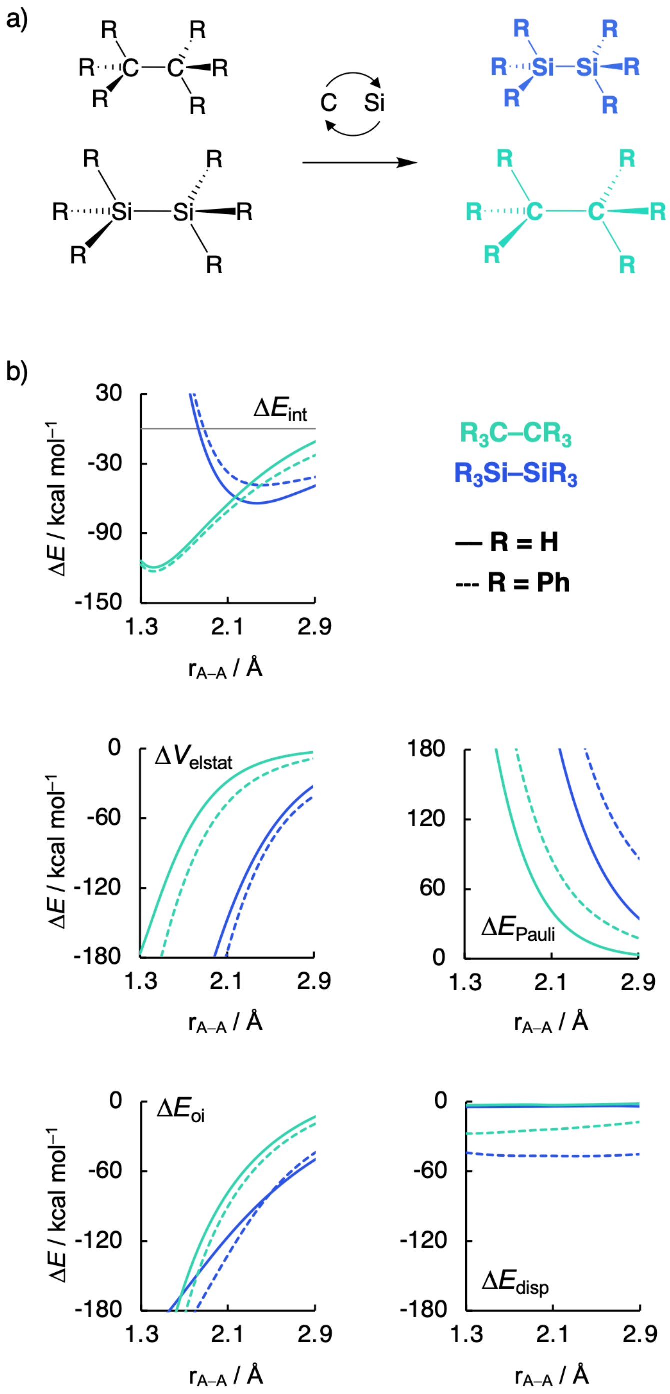

Figure S9. a) Numerical experiment in which the Si–Si bond in $R_3A–AR_3$ (A = C, Si; $R_3 = H_3, Ph_3$) is replaced by the C–C bond, and vice versa, keeping the other geometrical parameters unchanged; and b) energy decomposition analysis as a function of the A–A distance in the constrained $R_3A–AR_3$ geometry (A = C, Si; $R_3 = H_3, Ph_3$) of the above numerical experiment, computed at BLYP-D3(BJ)/TZ2P.

Table S6. Bond enthalpies (Δ*H*; in kcal mol^{-1}) of the mixed R_3A–AR_3 systems (A = C, Si; R_3 = H_3, Ph_3, *t*-Bu_3).[a]

	$^{\bullet}AR_3$					
$R_3A^{\bullet}$	$^{\bullet}CH_3$	$^{\bullet}CPh_3$	$^{\bullet}C$*t*-Bu_3	$^{\bullet}SiH_3$	$^{\bullet}SiPh_3$	$^{\bullet}Si$*t*-Bu_3
$H_3C^{\bullet}$	–85.2	–54.7	–65.2	–83.2	–83.0	–84.3
$Ph_3C^{\bullet}$	–54.7	–4.6	[b]	–50.4	–54.9	–21.3
t-$Bu_3C^{\bullet}$	–65.2	[b]	[b]	–60.0	–37.2	[b]
$H_3Si^{\bullet}$	–83.2	–50.4	–60.0	–71.4	–70.2	–73.4
$Ph_3Si^{\bullet}$	–83.0	–54.9	–37.2	–70.2	–78.5	–69.7
t-$Bu_3Si^{\bullet}$	–84.3	–21.3	[b]	–73.4	–69.7	–41.0

[a] Computed at BLYP-D3(BJ)/TZ2P at 298.15 K and 1 atm. [b] Unbonded systems wherein the A–A electron-pair bond is not formed; see ref. 32 for more details.

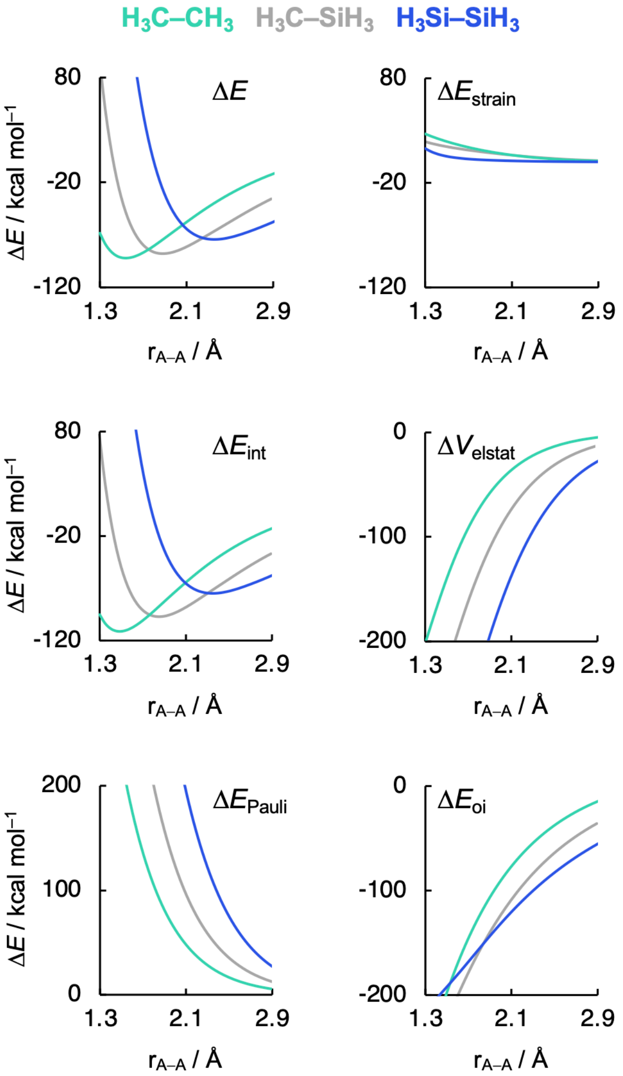

Figure S10. Activation strain model and energy decomposition analysis terms as a function of the A–A distance in the mixed H_3A–AH_3 system (A = C, Si). The dispersion energy ΔE_{disp} is nearly constant and, therefore, not shown, computed at BLYP-D3(BJ)/TZ2P.

Table S7. Bond enthalpies (Δ*H*; in kcal mol^{-1}) of the $R_3A–AR_3$ systems (A = C, Si, Ge, Sn, Pb; R_3 = H_3, Ph_3, *t*-Bu_3).[a]

	A				
**R₃**	C	Si	Ge	Sn	Pb
H_3	–85.2	–71.4	–65.4	–55.6	–43.8
Ph_3	–4.6	–78.5	–73.8	–63.9	–48.7
t-Bu_3	[b]	–41.0	–46.8	–56.6	–45.4

[a] Computed at BLYP-D3(BJ)/TZ2P for A = C, Si and at ZORA-BLYP-D3(BJ)/TZ2P for A = Ge, Sn, Pb at 298.15 K and 1 atm. [b] Unbonded systems wherein the C–C electron-pair bond is not formed; see ref. 32 for more details.

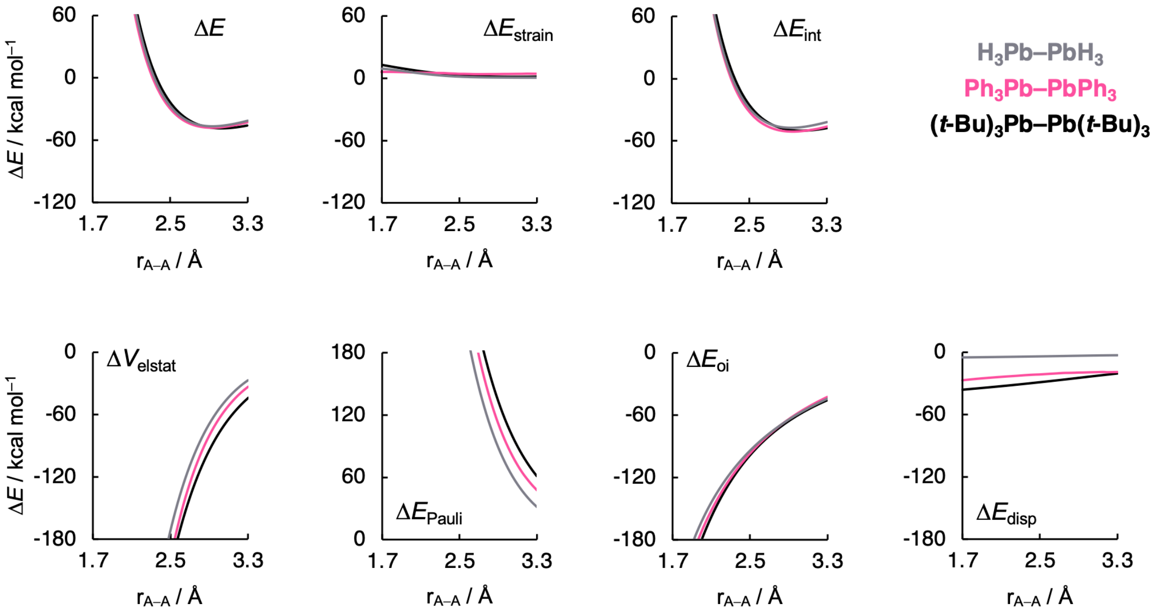

Figure S11. Activation strain model (top) and energy decomposition analysis (bottom) as a function of the Pb–Pb distance in $R_3Pb–PbR_3$ (R_3 = H_3, Ph_3, *t*-Bu_3), computed at ZORA-BLYP-D3(BJ)/TZ2P.

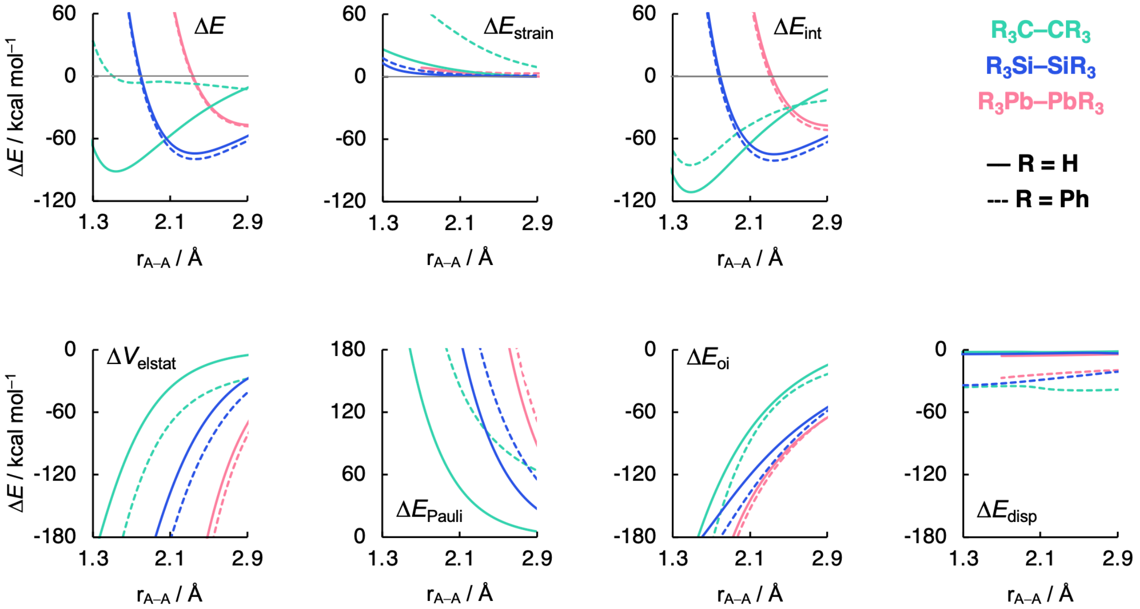

Figure S12. Activation strain model (top row) and energy decomposition analysis (bottom row) as a function of the A–A distance in $R_3A–AR_3$ (A = C, Si, Pb; R_3 = H_3, Ph_3), computed at BLYP-D3(BJ)/TZ2P for A = C, Si and at ZORA-BLYP-D3(BJ)/TZ2P for A = Pb.

Table S8. Cartesian coordinates (in Å), energies (electronic *E* and enthalpy *H*, in kcal mol^{-1}), number of imaginary frequencies (N_{imag}), and total spin number (*S*) of the equilibrium geometries of all R_3A–AR_3 systems studied herein, computed at BLYP-D3(BJ)/TZ2P for A = C, Si and at ZORA-BLYP-D3(BJ)/TZ2P for A = Ge, Sn, Pb.

H_3C–CH_3 (1C)
E = -900.79
H = -852.50
$\boldsymbol{N_{imag}}$ = 0
S = 0

C	0.000000	0.000000	0.769063
H	0.511179	0.885389	1.167098
H	0.511179	-0.885389	1.167098
H	-1.022359	0.000000	1.167098
C	0.000000	0.000000	-0.769063
H	-0.511179	0.885389	-1.167098
H	-0.511179	-0.885389	-1.167098
H	1.022359	0.000000	-1.167098

Me_3C–CMe_3 (2C)
E = -3096.32
H = -2939.86
$\boldsymbol{N_{imag}}$ = 0
S = 0

C	-0.023939	0.030359	0.789947
C	0.665743	1.303784	1.342074
C	0.680171	-1.197270	1.421776
C	-1.492003	0.037618	1.286750
C	0.046562	-0.019958	-0.804264
C	-0.432835	1.314743	-1.429468
C	-0.847845	-1.152250	-1.369950
C	1.494659	-0.275299	-1.294022
H	2.201398	0.457590	-0.889711
H	1.532269	-0.199603	-2.387683
H	1.847273	-1.275335	-1.021773
H	-0.698840	-1.232568	-2.453730
H	-1.911244	-0.956644	-1.198290
H	-0.605031	-2.126598	-0.932052
H	0.248357	2.140628	-1.200476
H	-1.435046	1.593802	-1.086277
H	-0.474836	1.215918	-2.521123
H	-1.512036	0.179514	2.374278
H	-2.001116	-0.906904	1.069404
H	-2.074409	0.850181	0.838839
H	1.698078	1.399363	0.988197
H	0.696489	1.260296	2.437706
H	0.124428	2.214025	1.064632
H	1.760391	-1.184402	1.244242
H	0.282419	-2.142995	1.037754
H	0.525118	-1.192202	2.507684

$PhMe_2C$–CMe_2Ph (3C)
E = -5394.08
H = -5168.64
$\boldsymbol{N_{imag}}$ = 0
S = 0

C	0.690283	-0.430698	-0.005092

C	0.780705	-1.307856	-1.282944
C	0.754691	-1.318863	1.254093
C	1.924789	0.508181	-0.043540
H	1.760488	-1.216154	4.526751
H	-0.133318	-4.432032	2.378756
H	0.837514	-3.536170	4.497203
H	2.839508	-0.087044	0.053535
H	1.919481	1.251990	0.754951
H	1.976097	1.042409	-0.996895
H	-0.084222	-1.959288	-1.425489
H	1.671307	-1.943687	-1.227048
H	0.875341	-0.680806	-2.173960
H	-0.179369	-3.052540	0.354816
C	1.301807	-0.838467	2.458182
C	1.333806	-1.624757	3.612962
C	0.814759	-2.922423	3.599219
C	0.270964	-3.422160	2.412544
C	0.246249	-2.630692	1.259306
H	1.696925	0.169713	2.510362
C	-0.690283	0.430698	-0.005092
C	-0.754691	1.318863	1.254093
C	-1.924789	-0.508181	-0.043540
C	-0.780705	1.307856	-1.282944
H	0.084222	1.959288	-1.425489
H	-1.671307	1.943687	-1.227048
H	-0.875341	0.680806	-2.173960
H	-2.839508	0.087044	0.053535
H	-1.919481	-1.251990	0.754951
H	-1.976097	-1.042409	-0.996895
H	0.179369	3.052540	0.354816
C	-1.301807	0.838467	2.458182
C	-1.333806	1.624757	3.612962
C	-0.814759	2.922423	3.599219
C	-0.270964	3.422160	2.412544
C	-0.246249	2.630692	1.259306
H	-1.696925	-0.169713	2.510362
H	-1.760488	1.216154	4.526751
H	-0.837514	3.536170	4.497203
H	0.133318	4.432032	2.378756

$Ph_2MeC–CMePh_2$ (4C)
E = -7685.67
H = -7391.53
N_{imag} = 0
S = 0

C	-0.591765	2.451963	-1.058716
H	-0.427198	0.354254	-3.712709
H	-1.203828	2.364175	-4.880808
C	0.167603	-0.079190	-1.131317
H	-1.633972	4.474820	-3.608955
H	-1.231079	4.505506	-1.145173
H	-0.409434	2.523917	0.003614
H	0.572217	-2.771654	-1.778095
C	-0.865883	-1.177052	-1.511601
C	-2.241587	-0.885100	-1.555198
C	-3.187083	-1.866552	-1.861596
C	-2.777997	-3.173602	-2.147912
C	-1.415363	-3.478855	-2.123173

C	-0.474649	-2.492880	-1.806697
H	-2.579188	0.127900	-1.360120
H	-4.243331	-1.605961	-1.884483
C	1.524060	-0.409582	-1.829057
H	-3.510887	-3.939424	-2.392792
H	1.974251	-1.317675	-1.429956
H	2.247374	0.395585	-1.708115
H	1.363581	-0.557927	-2.899390
H	-1.076587	-4.489087	-2.343285
C	-0.341942	1.257340	-1.749796
C	-0.578711	1.265270	-3.141050
C	-1.033057	2.403156	-3.806836
C	-1.276284	3.584493	-3.096432
C	-1.052575	3.598868	-1.719802
C	1.586540	0.886923	2.472083
H	1.929987	2.011786	-0.708817
C	2.967436	2.778367	0.986900
H	3.498653	3.514871	0.387380
C	0.343485	-0.092057	0.497344
C	2.072999	1.902477	0.356356
H	3.867927	3.393620	2.853684
C	2.471390	1.757362	3.107532
H	2.617378	1.683156	4.183305
H	1.078205	0.135230	3.068944
C	3.173607	2.713742	2.364832
H	-1.123392	-2.362775	1.221663
C	0.839487	-1.505072	0.916205
C	2.214462	-1.800571	0.959463
C	2.677515	-3.073275	1.301273
C	1.771320	-4.089187	1.624436
C	0.402955	-3.810660	1.600415
C	-0.053949	-2.536089	1.248344
H	2.935791	-1.020815	0.736197
H	3.748038	-3.267186	1.322843
H	2.128275	-5.080091	1.896966
H	-0.318097	-4.586642	1.848827
C	1.359322	0.936053	1.080090
C	-1.025101	0.182307	1.195642
H	-0.932805	0.013417	2.270967
H	-1.809830	-0.473661	0.820826
H	-1.356510	1.208883	1.045872

$Ph_3C–CPh_3$ (5C)
E = -9975.43
H = -9613.99
N_{imag} = 0
S = 0

C	0.999963	0.053702	-0.713773
C	0.778255	1.113184	-1.839317
C	0.459374	2.455638	-1.541134
C	0.244973	3.400482	-2.544991
C	0.372581	3.048568	-3.892930
C	0.749649	1.743445	-4.208662
C	0.956194	0.797505	-3.196979
C	2.247010	0.478355	0.125120
C	3.046384	1.573258	-0.244448
C	4.165876	1.957119	0.504161
C	4.534826	1.244001	1.644507

C	3.786106	0.116945	1.999825
C	2.676172	-0.264967	1.245717
C	1.306764	-1.322444	-1.385616
C	2.575718	-1.919440	-1.294661
C	2.856564	-3.153640	-1.893633
C	1.876373	-3.827998	-2.621421
H	0.379460	2.781063	-0.515826
H	-0.016024	4.419176	-2.265980
H	0.201399	3.783118	-4.676892
H	0.887839	1.447910	-5.246726
H	1.254520	-0.200044	-3.489740
H	2.804418	2.151270	-1.126061
H	4.747013	2.819621	0.184156
H	5.398231	1.545198	2.233807
H	4.068017	-0.478594	2.865622
H	2.148320	-1.158249	1.540590
H	3.369606	-1.430447	-0.746482
H	3.851302	-3.581151	-1.785215
H	2.088111	-4.791657	-3.079668
H	-0.152889	-3.712923	-3.359699
H	-0.620370	-1.554635	-2.347322
C	0.622326	-3.226468	-2.771365
C	0.350736	-1.992828	-2.178838
C	-0.408677	-0.058099	0.298922
C	-0.194370	-1.116021	1.427723
C	0.143019	-2.455452	1.137070
C	0.339555	-3.399392	2.145520
C	0.174900	-3.049801	3.490023
C	-0.219384	-1.747680	3.796931
C	-0.407763	-0.802606	2.780865
C	-0.710086	1.320543	0.967636
C	0.244610	1.978317	1.772979
C	-0.017702	3.215911	2.361168
C	-1.261481	3.834302	2.194473
C	-2.241550	3.172262	1.455358
C	-1.969837	1.933719	0.861046
C	-1.654992	-0.482574	-0.541086
C	-2.085681	0.262533	-1.660041
C	-3.191090	-0.123504	-2.418767
C	-3.933588	-1.256639	-2.070001
C	-3.563547	-1.971826	-0.931243
C	-2.448626	-1.584009	-0.178032
H	0.252174	-2.779353	0.114036
H	0.615645	-4.415747	1.872536
H	0.331472	-3.783749	4.277585
H	-0.386013	-1.453984	4.831339
H	-0.722067	0.192167	3.066446
H	1.207089	1.526423	1.954670
H	0.756255	3.692238	2.959367
H	-1.465936	4.801247	2.649094
H	-3.229093	3.612869	1.334358
H	-2.763569	1.454427	0.304210
H	-1.562854	1.160322	-1.950075
H	-3.474122	0.473529	-3.283152
H	-4.793198	-1.561130	-2.663146
H	-4.139909	-2.839466	-0.616553
H	-2.204423	-2.165210	0.700833

(*t*-Bu)$_3$C–C(*t*-Bu)$_3$ (6C)
E = -9569.14
H = -9088.53
N_{imag} = 0
S = 1

H	-1.20087500	-0.93588500	4.24777600
C	-0.97782000	-3.81061000	3.23331300
C	-1.57174300	-3.13237200	4.51334700
C	-2.86173000	-3.76759800	5.12027300
H	-3.07205400	-3.24303100	6.05941700
H	-3.72869400	-3.62576900	4.47201100
H	-2.77043900	-4.82624300	5.36091800
C	-0.52785800	-3.16644700	5.67902900
H	-0.34825000	-4.19689900	6.00294500
H	-0.91684100	-2.60497100	6.53872300
H	0.43470300	-2.73295800	5.40723600
C	-2.01817100	-1.65185600	4.24900900
H	-2.54428200	-1.57684000	3.29264300
H	-2.71581000	-1.34108400	5.03563700
C	-1.40939700	-5.27026400	2.86687800
C	-2.95574900	-5.39162200	2.63185500
H	-3.16196500	-6.30539100	2.06215900
H	-3.53990400	-5.44832700	3.54630400
H	-3.31975000	-4.54540200	2.04160300
C	-0.82248100	-5.87594900	1.55355900
H	-1.22224300	-5.38407600	0.66461900
H	-1.13642200	-6.92518600	1.51022100
H	0.26576700	-5.86727600	1.50244500
C	-0.96937300	-6.26499600	3.99233800
H	-1.32564500	-5.97944300	4.98219300
H	-1.36060000	-7.26704800	3.77217100
H	0.12254300	-6.33239600	4.03921800
C	0.28763200	-3.18974400	2.55189500
C	1.56572300	-4.00890700	2.93280400
H	2.42982300	-3.62432500	2.37508100
H	1.78208100	-3.90740500	4.00141200
H	1.47567900	-5.07291500	2.71352300
C	0.15026700	-3.12379500	0.99050600
H	0.34336700	-4.06778700	0.48826700
H	0.87105800	-2.39757300	0.59675100
H	-0.85017000	-2.78160000	0.70958600
C	0.64029400	-1.71438900	2.91921700
H	0.76002100	-1.53377600	3.98694800
H	1.60102000	-1.48167700	2.44568200
H	-0.09291000	-1.01001000	2.52134800
H	-4.19151500	0.03328400	2.50135200
C	-4.40575800	-1.52774400	-0.07788900
C	-3.37330900	-0.48157700	-0.61703400
C	-4.11924900	0.72166800	-1.28450300
H	-3.39234600	1.49306100	-1.57115000
H	-4.63927000	0.39515100	-2.19114000
H	-4.85655700	1.18681900	-0.63005100
C	-2.42084500	0.04921000	0.51094200
H	-2.86491400	0.81379700	1.14251600
H	-1.52968000	0.49145400	0.05019200
H	-2.08813700	-0.77508800	1.14882600
C	-2.36513200	-0.97263600	-1.70267600
H	-2.83233700	-1.40916700	-2.58479700

H	-1.79873600	-0.09725400	-2.04073900
C	-4.67363300	-2.83497900	-0.89671800
C	-3.37646400	-3.69826500	-1.07887800
H	-3.66129900	-4.72398500	-1.34113600
H	-2.71338500	-3.34206000	-1.86251200
H	-2.81233800	-3.74156000	-0.14249400
C	-5.67691500	-3.86297000	-0.28618300
H	-5.27567100	-4.33967700	0.61032300
H	-5.82761900	-4.65260100	-1.03125100
H	-6.65887400	-3.45028500	-0.05710700
C	-5.27213200	-2.48313800	-2.29946700
H	-4.66221500	-1.77633100	-2.86222300
H	-5.36519700	-3.39734400	-2.90043200
H	-6.27097100	-2.04748100	-2.19189100
C	-5.40962000	-1.10669700	1.04727300
C	-6.82135100	-0.82017100	0.43521800
H	-7.53949300	-0.62172300	1.24176600
H	-6.78715700	0.06350900	-0.21048700
H	-7.20893200	-1.64824100	-0.15839600
C	-5.53326400	-2.19448800	2.17086500
H	-6.17583300	-3.03075200	1.90934900
H	-5.95623300	-1.73423700	3.07158700
H	-4.54597500	-2.58643600	2.43286300
C	-5.06484000	0.17884600	1.86249400
H	-4.91219700	1.06919900	1.25341300
H	-5.91604900	0.38426100	2.52173100
H	-1.64394800	-1.68414600	-1.29571300

$H_3Si–SiH_3$ (1Si)
E = -690.70
H = -656.96
N_{imag} = 0
S = 0

Si	0.000000	0.000000	1.178149
H	0.698791	1.210341	1.695858
H	0.698791	-1.210341	1.695858
H	-1.397581	0.000000	1.695858
Si	0.000000	0.000000	-1.178222
H	-0.698804	-1.210364	-1.695834
H	1.397608	0.000000	-1.695834
H	-0.698804	1.210364	-1.695834

$Me_3Si–SiMe_3$ (2Si)
E = -2947.16
H = -2800.98
N_{imag} = 0
S = 0

Si	1.178683	0.000193	0.000000
Si	-1.178683	-0.000193	0.000000
C	1.827894	-0.892465	-1.546125
C	1.827974	1.785454	-0.000001
C	1.827894	-0.892463	1.546127
C	-1.827894	0.892463	1.546127
C	-1.827894	0.892465	-1.546125
C	-1.827975	-1.785454	-0.000001
H	1.483586	-0.398499	-2.463409
H	2.926151	-0.901818	-1.562113
H	1.483424	-1.933785	-1.577059

H	1.483680	2.332933	0.886389
H	2.926235	1.803790	-0.000001
H	1.483682	2.332931	-0.886394
H	1.483424	-1.933783	1.577062
H	2.926151	-0.901816	1.562114
H	1.483586	-0.398496	2.463409
H	-1.483424	1.933783	1.577062
H	-2.926151	0.901816	1.562114
H	-1.483586	0.398496	2.463409
H	-1.483586	0.398499	-2.463409
H	-2.926151	0.901818	-1.562113
H	-1.483424	1.933785	-1.577059
H	-1.483680	-2.332933	0.886389
H	-2.926235	-1.803790	-0.000000
H	-1.483682	-2.332931	-0.886394

PhMe₂Si–SiMe₂Ph (3Si)

E = -5247.77

H = -5032.66

$\boldsymbol{N_{imag}}$ = 0

S = 0

C	1.657763	-1.131222	1.633575
H	2.732288	-1.186585	-4.690165
H	1.062555	-2.046767	1.726440
H	2.718674	-1.410929	1.658005
H	1.449200	-0.506247	2.510981
H	2.061618	-3.584913	-4.615515
H	1.083361	-4.528602	-2.525937
C	1.587588	-1.309209	-1.458846
C	2.134039	-0.796071	-2.652336
C	2.305648	-1.605856	-3.780666
C	1.930111	-2.953221	-3.739268
C	1.383545	-3.483193	-2.564707
C	1.213393	-2.669128	-1.441429
H	2.432660	0.249369	-2.706042
H	0.769516	-3.097935	-0.545743
H	2.081742	2.020865	0.800266
H	3.338213	1.165855	-0.116673
H	2.006301	1.974905	-0.967183
Si	1.239617	-0.203685	0.033953
C	2.263966	1.388287	-0.077424
Si	-1.076681	0.216880	-0.067657
H	-1.070135	2.127486	-1.677285
C	-1.697494	1.225364	1.414209
C	-1.486599	1.112502	-1.685954
H	-1.228551	2.217169	1.429555
H	-2.785290	1.363548	1.366060
H	-1.461885	0.732480	2.364819
H	-1.060140	0.587044	-2.548367
H	-2.571142	1.191994	-1.834071
C	-1.866236	-1.500454	-0.059048
C	-2.052356	-2.204692	1.148403
C	-2.523701	-3.521252	1.158011
C	-2.820703	-4.167236	-0.048111
C	-2.645646	-3.486714	-1.257806
C	-2.171828	-2.170941	-1.260066
H	-1.822366	-1.722870	2.097516
H	-2.659002	-4.043295	2.103552

H	-3.185911	-5.192232	-0.043997
H	-2.871486	-3.982949	-2.199796
H	-2.026768	-1.667090	-2.213111

$Ph_2MeSi–SiMePh_2$ (4Si)
E = -7547.79
H = -7262.63
N_{imag} = 0
S = 0

C	-0.692463	2.721208	-1.804871
H	-0.595294	0.767413	-4.591778
H	-1.428374	2.856786	-5.612597
Si	0.179064	-0.046150	-1.843013
H	-1.807829	4.875360	-4.202450
H	-1.337219	4.781587	-1.757933
H	-0.490130	2.702815	-0.736338
H	0.399919	-3.025291	-2.193511
C	-1.057405	-1.421253	-2.229888
C	-2.433898	-1.140359	-2.344662
C	-3.369490	-2.164361	-2.522377
C	-2.945298	-3.496996	-2.588872
C	-1.583101	-3.796725	-2.477195
C	-0.652087	-2.769044	-2.296651
H	-2.776872	-0.108315	-2.299033
H	-4.427496	-1.924138	-2.610645
C	1.871266	-0.482600	-2.572987
H	-3.671453	-4.295528	-2.727397
H	2.262035	-1.404141	-2.126279
H	2.595194	0.316635	-2.377725
H	1.803376	-0.626654	-3.658308
H	-1.246473	-4.830630	-2.524075
C	-0.467157	1.569607	-2.582354
C	-0.744427	1.644365	-3.963266
C	-1.221106	2.822625	-4.544638
C	-1.435302	3.957424	-3.752343
C	-1.170904	3.904355	-2.380229
C	1.815138	1.200576	2.586893
H	2.017536	2.159210	-0.674736
C	3.133108	3.029758	0.939961
H	3.645345	3.739282	0.292928
Si	0.276486	-0.031433	0.508319
C	2.210078	2.129113	0.395334
H	4.113570	3.714725	2.739494
C	2.734484	2.097752	3.137701
H	2.937767	2.080186	4.206819
H	1.318494	0.484647	3.240438
C	3.396320	3.016263	2.313250
H	-1.143140	-2.647472	0.941545
C	0.843334	-1.780271	0.943508
C	2.214658	-2.084298	1.060918
C	2.649821	-3.395947	1.274789
C	1.717529	-4.435611	1.375896
C	0.351521	-4.154478	1.262288
C	-0.077972	-2.841465	1.045437
H	2.949872	-1.284859	0.988681
H	3.713841	-3.607417	1.364330
H	2.053705	-5.457045	1.542654
H	-0.378707	-4.958108	1.335983

C	1.531185	1.199352	1.205329
C	-1.442046	0.285154	1.238575
H	-1.433285	0.158853	2.328188
H	-2.178201	-0.408772	0.816660
H	-1.776373	1.304376	1.014076

Ph_3Si–$SiPh_3$ (5Si)
E = -9853.43
H = -9497.95
N_{imag} = 0
S = 0

Si	1.493094	0.094075	-1.070793
C	1.154835	1.394213	-2.395398
C	0.761424	2.697633	-2.023001
C	0.442562	3.657492	-2.987319
C	0.507815	3.333474	-4.348213
C	0.899410	2.047862	-4.736264
C	1.219707	1.089470	-3.768009
C	2.952933	0.600548	0.011653
C	3.700008	1.765189	-0.246435
C	4.736559	2.161745	0.606198
C	5.046786	1.397651	1.736082
C	4.318351	0.232587	2.006814
C	3.282625	-0.159443	1.154487
C	1.788970	-1.598550	-1.849887
C	2.999339	-2.295275	-1.674512
C	3.176108	-3.579538	-2.201851
C	2.142378	-4.192374	-2.917798
H	0.691040	2.962977	-0.970903
H	0.137973	4.655312	-2.677639
H	0.255108	4.078498	-5.100008
H	0.954280	1.790338	-5.792389
H	1.516455	0.092072	-4.084042
H	3.465904	2.372242	-1.117887
H	5.300325	3.067120	0.388960
H	5.850329	1.706744	2.401582
H	4.553203	-0.366601	2.884397
H	2.718265	-1.058858	1.388780
H	3.809420	-1.833586	-1.114731
H	4.119610	-4.101321	-2.052655
H	2.277354	-5.192247	-3.325396
H	0.123482	-3.983443	-3.662929
H	-0.192618	-1.725183	-2.723087
C	0.932633	-3.512890	-3.107786
C	0.758732	-2.231398	-2.578237
Si	-0.416281	-0.059087	0.304453
C	-0.081394	-1.358521	1.630575
C	0.316199	-2.661200	1.260094
C	0.629762	-3.621221	2.226004
C	0.554984	-3.298037	3.586605
C	0.159374	-2.013079	3.972738
C	-0.155642	-1.054548	3.002880
C	-0.709856	1.634753	1.081735
C	0.319826	2.263949	1.814033
C	0.149339	3.546997	2.340880
C	-1.056329	4.231786	2.144098
C	-2.089514	3.622576	1.424284
C	-1.916201	2.336693	0.899755

C	-1.875646	-0.564930	-0.778981
C	-2.205191	0.195548	-1.921557
C	-3.239792	-0.196971	-2.775025
C	-3.967215	-1.363002	-2.505781
C	-3.657139	-2.127592	-1.376183
C	-2.621745	-1.730557	-0.522381
H	0.393750	-2.925889	0.208313
H	0.937580	-4.618526	1.917854
H	0.803458	-4.043200	4.339671
H	0.097160	-1.756204	5.028612
H	-0.455815	-0.057705	3.317449
H	1.268205	1.753645	1.963968
H	0.958027	4.014661	2.899128
H	-1.188576	5.232926	2.549473
H	-3.029888	4.148467	1.269845
H	-2.725802	1.877899	0.336922
H	-1.641519	1.095616	-2.154873
H	-3.474480	0.402598	-3.652390
H	-4.769832	-1.672484	-3.172217
H	-4.220099	-3.033746	-1.160107
H	-2.387663	-2.338058	0.348765

(*t*-Bu)$_3$Si–Si(*t*-Bu)$_3$ (6Si)
E = -9484.26
H = -9007.76
N_{imag} = 0
S = 0

H	-1.356809	-0.140138	3.265088
Si	-2.250523	-2.963760	2.005424
C	-2.269250	-2.077639	3.813928
C	-3.531946	-2.465762	4.623856
H	-3.515467	-1.933716	5.586945
H	-4.455064	-2.180868	4.116073
H	-3.580461	-3.533680	4.846411
C	-1.040566	-2.462887	4.685349
H	-0.930165	-3.537499	4.832606
H	-1.166770	-2.007169	5.679296
H	-0.105349	-2.075737	4.274790
C	-2.259017	-0.533011	3.736765
H	-3.117093	-0.138517	3.196813
H	-2.297317	-0.125542	4.758551
C	-2.681450	-4.910763	2.288066
C	-4.186470	-5.173960	2.528138
H	-4.343826	-6.256980	2.645843
H	-4.559843	-4.692238	3.433311
H	-4.803781	-4.844133	1.695309
C	-2.279311	-5.765906	1.060060
H	-2.791925	-5.451757	0.149188
H	-2.563136	-6.812694	1.245961
H	-1.204361	-5.752576	0.868604
C	-1.947684	-5.517180	3.517409
H	-2.296807	-5.088717	4.459540
H	-2.169680	-6.594644	3.554004
H	-0.863511	-5.410424	3.474420
C	-0.363661	-2.862881	1.308467
C	0.603819	-3.828482	2.049368
H	1.621952	-3.654966	1.668788
H	0.630303	-3.675916	3.128583

H	0.367487	-4.877500	1.857595
C	-0.247167	-3.223770	-0.190897
H	-0.546141	-4.251857	-0.401771
H	0.803609	-3.118925	-0.501417
H	-0.840447	-2.566959	-0.823415
C	0.223258	-1.436948	1.460966
H	0.303248	-1.121223	2.503204
H	1.239141	-1.422322	1.038286
H	-0.359160	-0.688064	0.921411
H	-4.975750	-0.387331	2.948502
Si	-4.015114	-1.785636	0.293722
C	-3.324666	-0.017491	-0.379423
C	-4.432134	0.846489	-1.046099
H	-3.961396	1.752592	-1.457106
H	-4.941347	0.343671	-1.868632
H	-5.185808	1.177736	-0.328242
C	-2.720345	0.866066	0.736845
H	-3.452436	1.145867	1.496156
H	-2.342843	1.797577	0.288171
H	-1.884292	0.385207	1.240090
C	-2.208562	-0.208586	-1.437123
H	-2.559686	-0.720725	-2.335334
H	-1.840489	0.780129	-1.749941
C	-4.369584	-2.942674	-1.316220
C	-3.084220	-3.547590	-1.927696
H	-3.361999	-4.184622	-2.781268
H	-2.396614	-2.787144	-2.301492
H	-2.541015	-4.170335	-1.220167
C	-5.290380	-4.138340	-0.964804
H	-4.860811	-4.781110	-0.194448
H	-5.428269	-4.758605	-1.863207
H	-6.282781	-3.825392	-0.633822
C	-5.060990	-2.167984	-2.473514
H	-4.410281	-1.404853	-2.906309
H	-5.293112	-2.883811	-3.276785
H	-5.997788	-1.692721	-2.181595
C	-5.788269	-1.437005	1.182877
C	-6.919673	-1.118868	0.164939
H	-7.828665	-0.861315	0.729685
H	-6.693881	-0.275964	-0.488664
H	-7.167178	-1.979896	-0.459929
C	-6.299809	-2.636160	2.014953
H	-6.484790	-3.523237	1.406941
H	-7.254412	-2.359529	2.487965
H	-5.611155	-2.913827	2.810020
C	-5.709235	-0.233145	2.155186
H	-5.472413	0.704966	1.649024
H	-6.687869	-0.102043	2.640968
H	-1.352621	-0.759254	-1.043053

H_3C–CPh_3
E = -5447.20
H = -5243.05
N_{imag} = 0
S = 0

C	1.06375200	0.05708100	-0.76004700
C	0.79493400	1.10473000	-1.86781800
C	0.23064600	2.34912500	-1.53387400

C	0.00998000	3.32771900	-2.50567700
C	0.35509900	3.08610400	-3.84059400
C	0.92521700	1.85839000	-4.18522100
C	1.14300000	0.87936900	-3.20772800
C	2.27071600	0.49694700	0.10396300
C	3.19241000	1.45677900	-0.34000300
C	4.30158600	1.80819200	0.43913700
C	4.51252900	1.20375500	1.68077000
C	3.60504700	0.23893300	2.13309800
C	2.50116500	-0.11005800	1.35148100
C	1.34262100	-1.32791000	-1.39329200
C	2.63773000	-1.85999400	-1.47857500
C	2.87192600	-3.09685200	-2.09203000
C	1.81264200	-3.82770300	-2.63526600
H	-0.02550400	2.56766300	-0.50112000
H	-0.42841300	4.28151700	-2.21888500
H	0.18333900	3.84658800	-4.59928300
H	1.20427400	1.65607100	-5.21737600
H	1.59007500	-0.06631000	-3.49599400
H	3.04835600	1.93596600	-1.30283200
H	4.99999100	2.55663200	0.07007300
H	5.37243500	1.47757300	2.28823200
H	3.75828200	-0.24664700	3.09480800
H	1.82268600	-0.87662300	1.71452200
H	3.47493500	-1.30717500	-1.06532500
H	3.88695800	-3.48574500	-2.14306500
H	1.99279600	-4.78980000	-3.10997300
H	-0.31972300	-3.85877100	-2.98801800
H	-0.72473300	-1.67685900	-1.92803300
C	0.51591100	-3.30564800	-2.56384800
C	0.28726200	-2.07063700	-1.95262900
C	-0.20256500	-0.04858400	0.15185500
H	-0.13607500	-0.91959500	0.80876800
H	-1.10780200	-0.14658000	-0.45268600
H	-0.30551400	0.84240000	0.77644500

H_3C–C(*t*-Bu)$_3$
E = -5256.88
H = -4990.76
N_{imag} = 0
S = 0

H	-1.22772000	-0.88704200	4.11341100
C	-1.25755200	-3.62542500	2.96587500
C	-1.63727400	-3.06935700	4.47645500
C	-2.92413000	-3.70723900	5.08421600
H	-3.13204100	-3.19981200	6.03372000
H	-3.79970200	-3.55698600	4.44682500
H	-2.83066100	-4.76934000	5.30700500
C	-0.52848600	-3.27447400	5.54620100
H	-0.23196900	-4.31809200	5.65219800
H	-0.91654900	-2.94554900	6.51850900
H	0.36865500	-2.68716400	5.34947800
C	-2.00399000	-1.54703800	4.48691100
H	-2.91873800	-1.35007600	3.92064600
H	-2.20289000	-1.25569400	5.52520700
C	-1.48765200	-5.25610000	2.81318100
C	-2.99930000	-5.64536200	2.68907500
H	-3.06175500	-6.73859700	2.63043600

H	-3.62148700	-5.33441900	3.52208000
H	-3.44149300	-5.25187600	1.76938100
C	-0.89815000	-5.86159000	1.50242400
H	-1.30719700	-5.38499600	0.60742700
H	-1.19050300	-6.91757600	1.46133700
H	0.18938600	-5.83219900	1.44946400
C	-0.89423300	-6.11090600	3.96777700
H	-1.38896000	-5.93939400	4.92397500
H	-1.03361800	-7.17167000	3.72381500
H	0.17464800	-5.94849500	4.10707000
C	0.24729100	-3.14786100	2.47331900
C	1.41908500	-4.00529400	3.02793100
H	2.36550200	-3.55319500	2.70532500
H	1.43417400	-4.04542500	4.11717400
H	1.41426800	-5.02883900	2.65266200
C	0.39602800	-3.12714200	0.91464800
H	0.20967700	-4.07735800	0.42471700
H	1.42752400	-2.83942500	0.67857500
H	-0.25569000	-2.37639000	0.45872900
C	0.59949800	-1.67413300	2.84254800
H	0.70085200	-1.49632700	3.91238500
H	1.56871300	-1.43821500	2.38732900
H	-0.12335800	-0.96256200	2.43436200
H	-2.24407900	-3.42407100	1.00679100
C	-2.26416800	-2.95385300	1.99246100
H	-2.04011700	-1.89415500	1.85143300
H	-3.28890000	-3.02250700	2.36428700

Ph_3C–C(*t*-Bu)$_3$

E = -9775.08

H = -9354.37

N_{imag} = 0

S = 1

H	-1.61680900	-1.65124300	5.13915900
C	-0.55474200	-4.08147400	3.65584700
C	-1.46085300	-3.87693500	4.91658000
C	-2.61986200	-4.89945000	5.13448600
H	-3.07071700	-4.67737000	6.10873000
H	-3.40422900	-4.78333200	4.38510000
H	-2.30264400	-5.94130300	5.15980100
C	-0.60203800	-3.96865700	6.22176500
H	-0.22136800	-4.98616800	6.35981800
H	-1.22524500	-3.72042900	7.09126300
H	0.25384300	-3.29379000	6.22318800
C	-2.22928800	-2.51067800	4.88180700
H	-2.66616600	-2.34984100	3.89302300
H	-3.05539000	-2.54473500	5.60159100
C	-0.55897900	-5.46557000	2.92333600
C	-1.97881900	-5.84531700	2.37712700
H	-1.87540600	-6.64135900	1.63057100
H	-2.66357000	-6.20840900	3.13824300
H	-2.43137900	-4.98621700	1.87555200
C	0.33005400	-5.59925700	1.64737000
H	-0.06410600	-5.00823100	0.81928000
H	0.29716700	-6.64998200	1.33658000
H	1.37813500	-5.34427000	1.79991300
C	-0.04293700	-6.58838400	3.88377000
H	-0.58229400	-6.62794500	4.83011000

H	-0.14889500	-7.56717500	3.39716100
H	1.01747400	-6.43939800	4.11315500
C	0.59630800	-3.06086500	3.36318100
C	1.97614900	-3.66238600	3.79191300
H	2.78442900	-2.98439100	3.48690700
H	2.02388000	-3.77520200	4.88015700
H	2.17715000	-4.63847100	3.35043400
C	0.64751600	-2.63822700	1.85410500
H	1.09442900	-3.38211500	1.20072000
H	1.24458400	-1.72400100	1.75721800
H	-0.35827300	-2.40926600	1.49290800
C	0.52615000	-1.68047400	4.08835400
H	0.45151000	-1.74490800	5.17326200
H	1.45626900	-1.14639300	3.86167800
H	-0.29429900	-1.06898500	3.70987800
H	-1.32601900	-3.13215700	-0.42335900
C	-3.57731900	-2.09298200	0.70952200
H	-2.57419100	-0.46188500	2.65036800
H	-4.54462200	-4.20355400	2.13694300
C	-3.47329200	-3.25222100	-0.17412500
C	-4.61679700	-4.01271300	-0.53300800
C	-4.51227000	-5.13368000	-1.35295500
C	-3.26452000	-5.54106400	-1.84441500
C	-2.12265300	-4.80162700	-1.50940500
C	-2.22341200	-3.67703200	-0.69536500
H	-5.59234400	-3.69950900	-0.17328100
H	-5.40918100	-5.68985800	-1.61812900
H	-3.18394500	-6.42038100	-2.47928600
H	-1.14602900	-5.11442000	-1.87278700
C	-4.58404400	-2.07046900	1.76815300
C	-5.17957200	-0.85540400	2.19705100
C	-6.11769200	-0.83300300	3.22622800
C	-6.49701600	-2.01960600	3.86847900
C	-5.92875500	-3.23218300	3.45657200
C	-4.99522800	-3.25992900	2.42452900
H	-4.90832400	0.06981200	1.69751400
H	-6.56316800	0.11363800	3.52542800
H	-7.22489700	-1.99933100	4.67623900
H	-6.20422000	-4.15917300	3.95506300
C	-2.66193000	-0.96610100	0.54653700
C	-2.15607000	-0.60340800	-0.72934600
C	-1.26059300	0.45263600	-0.87949600
C	-0.83356200	1.18670100	0.23550200
C	-1.32575200	0.85238300	1.50391100
C	-2.22520700	-0.19863900	1.65788900
H	-2.49084500	-1.15203900	-1.60458600
H	-0.89845300	0.71176700	-1.87238000
H	-0.12930200	2.00691300	0.11714600
H	-0.99252100	1.40512500	2.37976400

H_3Si–$SiPh_3$
E = -5267.64
H = -5072.68
N_{imag} = 0
S = 0

Si	1.39721800	0.08571000	-1.00315100
C	1.08056200	1.36863000	-2.34896200
C	0.62203000	2.65770100	-2.00784900

C	0.40910000	3.63101600	-2.98810100
C	0.64834300	3.33144500	-4.33489300
C	1.10101800	2.05725300	-4.69288200
C	1.31471000	1.08597800	-3.70805000
C	2.87235900	0.60899600	0.04943700
C	3.70768900	1.67665100	-0.33038400
C	4.81463400	2.03684500	0.44665300
C	5.10631700	1.33399000	1.62034500
C	4.28657100	0.26947800	2.01477300
C	3.18107200	-0.08596500	1.23685400
C	1.72943800	-1.59902100	-1.78346400
C	3.00107600	-2.20032100	-1.72637200
C	3.23940000	-3.44015500	-2.33044600
C	2.20750500	-4.10218200	-3.00386300
H	0.43136600	2.90628400	-0.96483100
H	0.05535000	4.62018700	-2.70389300
H	0.48087800	4.08687600	-5.09991500
H	1.28839300	1.81936200	-5.73828500
H	1.66660200	0.09881400	-3.99937200
H	3.49125200	2.23150800	-1.24059800
H	5.44866100	2.86499200	0.13559700
H	5.96599500	1.61401300	2.22574900
H	4.50779800	-0.27997800	2.92775200
H	2.55323100	-0.91607800	1.55739300
H	3.81176700	-1.69585500	-1.20536700
H	4.22973600	-3.88826300	-2.27557000
H	2.39141000	-5.06668400	-3.47278800
H	0.12868900	-4.03126100	-3.59215000
H	-0.29340600	-1.84342100	-2.52516700
C	0.93570100	-3.52004300	-3.07084700
C	0.70132200	-2.28262900	-2.46464200
Si	-0.51099600	-0.07006900	0.37288900
H	-0.26752400	-0.99402400	1.51924900
H	-1.68097800	-0.58775700	-0.39534400
H	-0.87889400	1.26541400	0.92809900

H_3Si–Si(*t*-Bu)$_3$
E = -5103.64
H = -4849.60
$\boldsymbol{N_{imag}}$ = 0
S = 0

H	-1.42369300	-0.16847800	3.06333100
Si	-2.06826200	-3.06295800	2.14034000
C	-2.22131100	-2.09114400	3.83721400
C	-3.52338100	-2.47516900	4.58492700
H	-3.62355200	-1.84321100	5.48009200
H	-4.41347600	-2.31239300	3.96713300
H	-3.52326400	-3.51580600	4.92019500
C	-1.02344300	-2.36779300	4.78015400
H	-0.91789000	-3.43218000	5.01343100
H	-1.17220000	-1.83395400	5.73112200
H	-0.07591800	-2.02067000	4.35753500
C	-2.30481600	-0.56356500	3.57205500
H	-3.18543300	-0.30430100	2.97265100
H	-2.39894400	-0.03424500	4.53226000
C	-2.62691200	-4.93673300	2.29578100
C	-4.17234600	-5.02487200	2.41687600
H	-4.46950200	-6.08157500	2.49500100

H	-4.56335000	-4.50716400	3.29458500
H	-4.67180300	-4.61350900	1.53182800
C	-2.24300300	-5.74057700	1.02755900
H	-2.65474800	-5.29059800	0.11738300
H	-2.65546000	-6.75750500	1.10800800
H	-1.16104300	-5.83761600	0.90393700
C	-1.99078200	-5.63737800	3.52255500
H	-2.30693100	-5.18482500	4.46710500
H	-2.30128900	-6.69307000	3.54591900
H	-0.89672900	-5.61691500	3.48929900
C	-0.29432800	-2.87415100	1.32496900
C	0.74744500	-3.82753100	1.96243900
H	1.73231100	-3.65861800	1.50098400
H	0.85578100	-3.66148400	3.03908300
H	0.49290600	-4.88036100	1.80858700
C	-0.37252600	-3.16742400	-0.19774300
H	-0.71384900	-4.17960700	-0.42190500
H	0.62773400	-3.04862600	-0.64090700
H	-1.03897300	-2.46474100	-0.71162600
C	0.23130400	-1.42271700	1.46238900
H	0.42986500	-1.14502200	2.50104800
H	1.17966800	-1.33025000	0.91212500
H	-0.46675000	-0.69260000	1.03803200
Si	-3.59702700	-2.03676500	0.64761600
H	-3.05882300	-0.75802200	0.09512000
H	-4.89276500	-1.71402400	1.31620800
H	-3.91214600	-2.91833400	-0.51584100

$Ph_3Si–Si(\textit{t}\text{-}Bu)_3$
E = -9679.20
H = -9262.86
$\boldsymbol{N_{imag}}$ = 0
S = 0

H	-1.74271600	-0.30173400	3.55371300
Si	-1.99445100	-3.09678400	2.21942000
C	-2.33733900	-2.38074400	4.02907600
C	-3.60001400	-3.00486500	4.67584900
H	-3.77117500	-2.52699200	5.65243700
H	-4.49549500	-2.83942200	4.07358100
H	-3.49528100	-4.07758600	4.85573900
C	-1.14798500	-2.63621700	4.99214600
H	-0.91282800	-3.69903100	5.09507300
H	-1.40973700	-2.25838400	5.99228500
H	-0.23819400	-2.11785400	4.67913600
C	-2.58723600	-0.85158700	3.97461900
H	-3.48510800	-0.61161400	3.39765600
H	-2.74846100	-0.47460200	4.99597300
C	-2.27128900	-5.05309600	2.18589100
C	-3.78421700	-5.39183400	2.17820900
H	-3.91034400	-6.48513400	2.18644400
H	-4.30854100	-4.99660800	3.05090300
H	-4.27493700	-5.01821200	1.27476300
C	-1.67279300	-5.70691200	0.91446800
H	-2.09805400	-5.29131100	-0.00118400
H	-1.90803600	-6.78206500	0.92437800
H	-0.58501500	-5.61529700	0.86540600
C	-1.63132600	-5.75381300	3.41337600
H	-2.08977300	-5.44560800	4.35638800

H	-1.77691500	-6.84094000	3.32109100
H	-0.55548800	-5.57272400	3.48569800
C	-0.16128800	-2.64755900	1.63397500
C	0.90108400	-3.57590500	2.27962300
H	1.90235300	-3.25398400	1.95497800
H	0.88315300	-3.53721300	3.37215500
H	0.78344700	-4.61937300	1.97669000
C	-0.03186700	-2.77326500	0.09410200
H	-0.25933900	-3.77858700	-0.26684800
H	1.00311800	-2.54426500	-0.20194300
H	-0.67977000	-2.05955900	-0.42308500
C	0.22364900	-1.18782800	1.98501300
H	0.26268700	-1.00945600	3.06237200
H	1.22764800	-0.97987900	1.58483500
H	-0.45928700	-0.46307800	1.53746000
Si	-3.57761400	-2.06474100	0.66596500
H	-2.58923500	0.59695700	1.70258800
H	-5.74397100	-3.54260200	2.16961100
H	-2.05976500	-3.46709500	-1.53997400
C	-2.87639600	-0.49010500	-0.14856300
C	-2.75957200	-0.37783200	-1.54795200
C	-2.26816000	0.78681200	-2.14895300
C	-1.88991000	1.87920500	-1.36295200
C	-2.01427800	1.79848100	0.02831700
C	-2.49911100	0.62961100	0.62156500
H	-3.05931100	-1.20641600	-2.18266200
H	-2.18646100	0.84045800	-3.23301700
H	-1.50761300	2.78537600	-1.82830300
H	-1.73158100	2.64421600	0.65234500
C	-5.20304200	-1.54907600	1.51837400
C	-5.64754500	-0.21237600	1.50215600
C	-6.85320300	0.16395900	2.10530300
C	-7.65543600	-0.79321700	2.73336300
C	-7.24411600	-2.13047400	2.74648000
C	-6.03551700	-2.49745000	2.14825600
H	-5.05035600	0.54891800	1.00914600
H	-7.16667200	1.20596600	2.07878300
H	-8.59297800	-0.50254900	3.20280700
H	-7.86253700	-2.88789700	3.22445100
C	-4.06621600	-3.23374600	-0.75851400
C	-5.40918600	-3.59525600	-0.98279100
C	-5.77272900	-4.43373700	-2.04289500
C	-4.79946600	-4.92545100	-2.91763600
C	-3.46104100	-4.56532400	-2.72645600
C	-3.10481300	-3.73343400	-1.66141900
H	-6.18688400	-3.21612100	-0.32650700
H	-6.81886500	-4.69764400	-2.18676300
H	-5.08016500	-5.57740500	-3.74225600
H	-2.69362000	-4.93381400	-3.40460000

H_3C–SiH_3
E = -800.12
H = -759.58
$\boldsymbol{N_{imag}}$ = 0
S = 0

Si	0.00000000	0.00000000	0.73861700
H	0.69731000	1.20777600	1.26437700
H	0.69731000	-1.20777600	1.26437700

H	-1.39462000	0.00000000	1.26437700
C	0.00000000	0.00000000	-1.14970600
H	-0.51242800	-0.88755200	-1.53998400
H	1.02485700	0.00000000	-1.53998400
H	-0.51242800	0.88755200	-1.53998400

H_3C–$SiPh_3$
E = -5376.68
H = -5176.39
N_{imag} = 0
S = 0

Si	-0.00193200	0.00184100	0.96984100
C	-1.77536600	0.00626100	1.61306900
C	-2.71244900	-0.92803500	1.12609700
C	-4.02412200	-0.95856400	1.60876300
C	-4.42746400	-0.04988100	2.59488200
C	-3.51312300	0.88424400	3.09260400
C	-2.20105800	0.90963000	2.60538800
C	0.88165000	-1.53993500	1.60320200
C	0.33657200	-2.33724700	2.62760600
C	1.01853000	-3.45973300	3.11174000
C	2.26402800	-3.80693400	2.57818300
C	2.82474300	-3.02661700	1.55969200
C	2.13886700	-1.90647600	1.08078300
C	0.89296100	1.53288800	1.61301700
C	1.84206300	1.45083800	2.64981000
C	2.47486100	2.59716000	3.14443800
C	2.16880100	3.85262800	2.60894600
H	-2.41539000	-1.64693300	0.36349200
H	-4.73076700	-1.68857700	1.21839000
H	-5.44802300	-0.07099100	2.97192700
H	-3.82095600	1.59263900	3.85950600
H	-1.50022800	1.64215300	3.00008800
H	-0.63253800	-2.07965400	3.04959400
H	0.57798700	-4.06246800	3.90379900
H	2.79539600	-4.67969300	2.95245700
H	3.79406400	-3.29118800	1.14127600
H	2.59407100	-1.30814800	0.29257600
H	2.09132500	0.48003800	3.07301300
H	3.20610500	2.51030000	3.94596200
H	2.66033500	4.74499000	2.99134600
H	0.98466500	4.93089400	1.15753200
H	-0.13558500	2.90968800	0.28998800
C	1.22730600	3.95665700	1.57781600
C	0.59828300	2.80791100	1.08840700
C	-0.00710200	0.00696400	-0.91828300
H	1.01468200	-0.03318400	-1.31402100
H	-0.48500500	0.91430900	-1.30632800
H	-0.55618500	-0.85777500	-1.30948300

H_3C–Si(*t*-Bu)$_3$
E = -5211.91
H = -4951.28
N_{imag} = 0
S = 0

Si	-4.13501400	-1.65861000	0.14918600
C	-2.89614600	-2.46863000	1.34001000
C	-3.33736500	0.03385300	-0.42579400

C	-4.36830500	0.99276200	-1.07189100
H	-3.86106200	1.90865400	-1.41180100
H	-4.85927400	0.54658400	-1.94282800
H	-5.14810600	1.29649100	-0.36706700
C	-2.68320100	0.75369400	0.78568100
H	-3.40054400	1.01101100	1.56756700
H	-2.21925600	1.69074900	0.44238500
H	-1.89314700	0.14503200	1.23919500
C	-2.19886300	-0.20346600	-1.45188400
H	-2.56549800	-0.60646500	-2.39980600
H	-1.70564900	0.75513600	-1.67309400
C	-4.37722100	-2.90286000	-1.34234400
C	-3.00654700	-3.49706200	-1.76899300
H	-3.16211400	-4.21136800	-2.59163700
H	-2.30407300	-2.73938900	-2.12198600
H	-2.52956500	-4.04339100	-0.94781900
C	-5.25942900	-4.10930400	-0.92813600
H	-4.85999300	-4.62944400	-0.04983200
H	-5.28853800	-4.83641200	-1.75370900
H	-6.29175300	-3.82028100	-0.71331200
C	-5.03231200	-2.23599300	-2.57765100
H	-4.40798900	-1.44187100	-2.99796300
H	-5.18204000	-2.98647100	-3.36888300
H	-6.01119900	-1.80459500	-2.34456100
C	-5.79014400	-1.38765900	1.15842900
C	-7.01032500	-1.11213300	0.24461300
H	-7.90259400	-0.92809100	0.86241000
H	-6.86218700	-0.23171600	-0.38910400
H	-7.23733500	-1.96022300	-0.40829700
C	-6.09506200	-2.63860700	2.02787500
H	-6.24964900	-3.54274900	1.43560900
H	-7.01458800	-2.46191800	2.60618900
H	-5.29130200	-2.83804500	2.74517400
C	-5.65639700	-0.19907100	2.14568800
H	-5.55162300	0.76154300	1.63418200
H	-6.56277800	-0.14139500	2.76718300
H	-1.43092500	-0.88339500	-1.06526200
H	-4.80365900	-0.32047100	2.82362900
H	-1.87967200	-2.44914500	0.93058900
H	-3.15561900	-3.51590800	1.53261300
H	-2.87444600	-1.94987100	2.30522700

$Ph_3C–SiH_3$
E = -5345.91
H = -5147.91
N_{imag} = 0
S = 0

Si	0.00501200	-0.00455600	0.74585600
H	-1.34430400	-0.35590500	1.26075800
C	4.17117900	0.01364100	-2.51885300
C	3.26647300	-0.89761900	-3.06918800
C	1.93153200	-0.91576800	-2.64702600
C	-0.75689500	-1.26079700	-1.66796700
C	-0.43323700	-2.51894100	-1.12413000
H	0.98522900	-1.00030700	1.25242000
C	-1.96284600	1.63338500	-1.11366500
C	-2.64620700	2.77661200	-1.53253300
C	-2.09836000	3.60135500	-2.52202600

C	-0.86557100	3.26289300	-3.08569300
C	-0.18140000	2.11749300	-2.66102800
H	0.37883100	1.33952000	1.25831800
H	0.35198700	-2.59689100	-0.37622400
H	-0.81519200	-4.63741700	-1.10496500
H	-2.58780400	-4.51470800	-2.85910700
C	-1.08503400	-3.67967700	-1.54511600
C	-2.07724700	-3.61229500	-2.53019900
C	-2.40096000	-2.37270700	-3.08750100
C	-1.74761300	-1.21019900	-2.66081800
C	-0.71395400	1.28578400	-1.66381800
H	2.06050100	1.62220900	-0.39045700
H	4.41397600	1.63690900	-1.11124500
H	5.20906100	0.02472500	-2.84423100
H	3.59516300	-1.59933900	-3.83318900
H	1.24491100	-1.62945400	-3.08991100
H	-3.16394800	-2.30418700	-3.86035600
H	-2.01225800	-0.25822400	-3.10884800
H	-2.41796600	0.99251700	-0.36254100
H	-3.60846700	3.02109600	-1.08745800
H	-2.62769200	4.49344200	-2.84942000
H	-0.43013300	3.88880900	-3.86207400
H	0.77301700	1.87093400	-3.11418400
C	0.00081800	-0.00065400	-1.20330500
C	1.47178900	-0.02399700	-1.66572100
C	2.39261600	0.89682900	-1.12904600
C	3.72499300	0.91549100	-1.54561100

(*t*-Bu)$_3$C–SiH$_3$
E = -5153.53
H = -4894.75
N_{imag} = 0
S = 0

C	-4.11410100	-1.66585000	0.16540900
Si	-2.84745400	-2.49645000	1.38476900
C	-3.41645100	-0.24317100	-0.31244100
C	-4.40469000	0.77504200	-0.95070400
H	-3.83084700	1.64411400	-1.29650100
H	-4.92952000	0.36742900	-1.81457800
H	-5.14891900	1.14694400	-0.24677200
C	-2.69365500	0.50054700	0.85964000
H	-3.32722400	0.74119200	1.70665000
H	-2.29666100	1.44551300	0.46974000
H	-1.83333300	-0.06746500	1.22885600
C	-2.26304800	-0.40746700	-1.34896800
H	-2.58965200	-0.78231900	-2.31796300
H	-1.82854100	0.58422600	-1.52182000
C	-4.30955300	-2.73550100	-1.08222100
C	-2.96570900	-3.40788600	-1.51976100
H	-3.17599900	-4.06650000	-2.37099600
H	-2.18961700	-2.71645100	-1.83016000
H	-2.55683500	-4.04552700	-0.72897200
C	-5.19130900	-3.97426500	-0.73631100
H	-4.79303800	-4.53845300	0.10999400
H	-5.17758100	-4.64412300	-1.60419500
H	-6.23507100	-3.73307200	-0.54010700
C	-4.94342300	-2.12050300	-2.36316100
H	-4.30614400	-1.37608800	-2.84006900

H	-5.09608500	-2.92455300	-3.09414900
H	-5.91492500	-1.66389000	-2.17457700
C	-5.50127700	-1.43865700	1.03902700
C	-6.76933400	-1.17752100	0.17608500
H	-7.60959200	-0.96830600	0.85014400
H	-6.65969800	-0.31685700	-0.48356200
H	-7.05700300	-2.03536500	-0.43140700
C	-5.83185000	-2.64795500	1.97590800
H	-5.94313200	-3.60055800	1.46903100
H	-6.78011200	-2.43555100	2.48412400
H	-5.07931600	-2.76700800	2.76243600
C	-5.43806000	-0.25489300	2.05211900
H	-5.35802700	0.72468800	1.58290900
H	-6.37324800	-0.25773700	2.62443400
H	-1.46133400	-1.04735200	-0.97353800
H	-4.62301600	-0.37149200	2.76991500
H	-1.44897700	-2.39477800	0.89127600
H	-3.12874900	-3.94269000	1.58178100
H	-2.87397000	-1.86700500	2.73112200

Ph_3C–$SiPh_3$
E = -9927.84
H = -9569.39
N_{imag} = 0
S = 0

Si	-0.00074900	0.00052900	0.81623300
C	-1.76484600	-0.11369600	1.49861400
C	-2.65431700	-1.15160600	1.14530000
C	-3.94714500	-1.20804800	1.67311300
C	-4.38356800	-0.23174400	2.57613500
C	-3.51342700	0.79612900	2.95179800
C	-2.22073900	0.85117500	2.41928400
C	0.98017500	-1.47078400	1.49710200
C	0.36998600	-2.35437600	2.40998800
C	1.06324400	-3.44877100	2.93846200
C	2.39019500	-3.68455400	2.56645400
C	3.02014400	-2.81190200	1.67162800
C	2.32551100	-1.71820900	1.14772000
C	0.78289800	1.58554000	1.49768200
C	1.85212400	1.49887300	2.41179300
C	2.45275600	2.64645100	2.94088800
C	1.99410800	3.91354700	2.56822300
H	-2.34265400	-1.92214900	0.44848600
H	-4.61386400	-2.01607100	1.37822300
H	-5.39093600	-0.27480300	2.98542100
H	-3.83945500	1.55695000	3.65854800
H	-1.56176800	1.66063400	2.72053100
H	-0.66192300	-2.19164700	2.70808200
H	0.56539000	-4.11653300	3.63908400
H	2.93054200	-4.53737500	2.97251300
H	4.05466700	-2.98213800	1.37984000
H	2.83885700	-1.05817800	0.45684300
H	2.22661100	0.52384100	2.71052900
H	3.27916000	2.54915400	3.64247400
H	2.46211600	4.80789300	2.97476600
H	0.55495200	5.00385900	1.37994700
H	-0.50240000	2.98894700	0.45585200
C	0.92437500	4.02280300	1.67221900

C	0.32507800	2.87438800	1.14767700
C	-0.00044900	0.00063000	-1.18023200
C	1.47085900	-0.09446400	-1.64346000
C	2.41948400	0.85147600	-1.20259700
C	3.76025500	0.77183400	-1.58115200
C	4.19660500	-0.25425900	-2.42714700
C	3.26455800	-1.17935700	-2.90183400
C	1.92029000	-1.09569000	-2.51924900
C	-0.81809800	-1.22550300	-1.64533000
C	-0.47606500	-2.52033000	-1.20323200
C	-1.21501800	-3.64093300	-1.58471200
C	-2.31846000	-3.50472200	-2.43488100
C	-2.65092500	-2.23461200	-2.91043400
C	-1.90696600	-1.11295500	-2.52467300
C	-0.65308300	1.32167700	-1.64624500
C	-1.94448100	1.67455800	-1.20277500
C	-2.54549000	2.87431800	-1.58583900
C	-1.87684700	3.75948900	-2.43925400
C	-0.61177900	3.41045700	-2.91638200
C	-0.01240900	2.20582800	-2.52901900
H	2.10918800	1.66227700	-0.55564600
H	4.46404000	1.51441500	-1.21098500
H	5.24238600	-0.32322000	-2.71849300
H	3.57765400	-1.97366100	-3.57654700
H	1.22168100	-1.82686300	-2.90858500
H	0.37865400	-2.65786900	-0.55304000
H	-0.92613600	-4.62201400	-1.21351000
H	-2.90064100	-4.37539700	-2.72862000
H	-3.49286700	-2.10775200	-3.58809400
H	-2.18883400	-0.14195700	-2.91452100
H	-2.49023300	1.00478000	-0.55021200
H	-3.53881100	3.11605600	-1.21347400
H	-2.33975300	4.69862200	-2.73429200
H	-0.08186900	4.07421800	-3.59667200
H	0.96846700	1.96279000	-2.92031000

Ph_3C–Si(*t*-Bu)$_3$
E = -9729.02
H = -9309.41
N_{imag} = 0
S = 0

Si	-4.16066300	-1.61514200	0.14789500
C	-2.75768600	-2.62548000	1.45849800
C	-3.61898800	0.29507300	-0.15141300
C	-4.83086600	1.15633000	-0.61650200
H	-4.46389200	2.17192600	-0.82680300
H	-5.28913400	0.78260300	-1.53393300
H	-5.60317500	1.24915200	0.14564400
C	-3.11378600	0.94894900	1.16371600
H	-3.83564400	0.85899400	1.97815400
H	-2.94841300	2.02135700	0.98293400
H	-2.17322000	0.52851200	1.52188500
C	-2.53797800	0.53028200	-1.25476800
H	-2.98172100	0.54373100	-2.25225900
H	-2.08932700	1.52182200	-1.09681600
C	-4.25083800	-2.51895000	-1.64267100
C	-2.83832500	-2.94247800	-2.13031700
H	-2.91934600	-3.31743400	-3.16130700

H	-2.13239600	-2.10933900	-2.13661500
H	-2.39257600	-3.73498000	-1.52784800
C	-5.18649100	-3.76743500	-1.72696800
H	-5.15236000	-4.42954200	-0.86597300
H	-4.90169100	-4.35709000	-2.61047500
H	-6.22916600	-3.47621400	-1.86882300
C	-4.80530300	-1.55367100	-2.73240600
H	-4.14393800	-0.71126400	-2.92969800
H	-4.89408700	-2.11877600	-3.67213700
H	-5.79815100	-1.16897200	-2.49241500
C	-6.00825100	-1.61665500	0.93403400
C	-7.08800000	-1.39638200	-0.16706000
H	-8.06931400	-1.34041800	0.32710900
H	-6.94957800	-0.46308900	-0.71581700
H	-7.13873600	-2.21634400	-0.88197400
C	-6.34079000	-2.98669400	1.58584100
H	-6.19573300	-3.82205900	0.89764500
H	-7.39830600	-2.98517900	1.88871900
H	-5.74589400	-3.19782700	2.47532300
C	-6.30044300	-0.49749600	1.98461600
H	-6.55665300	0.44720300	1.50086000
H	-7.17800100	-0.79580600	2.57644800
H	-1.72995000	-0.19612500	-1.27268900
H	-5.49044300	-0.29292500	2.67953400
H	-1.09062800	-0.70481800	0.45215100
H	-3.18522000	-3.78545400	4.00267600
H	-0.37723100	-1.65889800	2.64083000
C	-2.53558500	-1.79232600	2.74793400
C	-3.62127700	-1.48513400	3.58518800
C	-3.47314000	-0.75512400	4.76420800
C	-2.20642500	-0.32080300	5.16749200
C	-1.10264300	-0.66626200	4.38588600
C	-1.26425500	-1.39879000	3.20371200
H	-4.60112700	-1.85518200	3.32767200
H	-4.34939200	-0.53351300	5.37033900
H	-2.08194000	0.25532800	6.08159300
H	-0.10119400	-0.37235700	4.69405300
C	-1.39398900	-2.80942100	0.74255500
C	-0.75127700	-4.05396500	0.61282200
C	0.49242500	-4.17529600	-0.01870700
C	1.14900200	-3.05141500	-0.52309000
C	0.55555100	-1.79691400	-0.35016800
C	-0.68302300	-1.68873800	0.28155900
H	-1.21304300	-4.95101800	1.00448700
H	0.94749200	-5.15983500	-0.10742000
H	2.11136800	-3.14655700	-1.02088200
H	1.05877300	-0.89747300	-0.69960000
C	-3.30613600	-4.02146800	1.85388200
C	-3.58795400	-4.97511400	0.86129600
C	-4.06517900	-6.25019300	1.16340800
C	-4.25624500	-6.63177600	2.49522000
C	-3.92430000	-5.72490300	3.50319300
C	-3.44695400	-4.44716600	3.18721300
H	-3.39673100	-4.72741400	-0.17093000
H	-4.27977600	-6.94689700	0.35540200
H	-4.63490600	-7.62146800	2.74043000
H	-4.02889200	-6.00752100	4.54887600

(*t*-Bu)$_3$C–SiPh$_3$
E = -9709.69
H = -9288.86
N_{imag} = 0
S = 0

C	-4.17588700	-1.57550500	0.01520400
Si	-2.88486200	-2.50068400	1.39706400
C	-3.32256800	-0.37351200	-0.76057100
C	-4.19928900	0.68452500	-1.50346300
H	-3.51958100	1.36902100	-2.02583800
H	-4.85749700	0.25221000	-2.25409300
H	-4.80134700	1.29446200	-0.83188800
C	-2.41686700	0.44997600	0.19991100
H	-2.96380600	0.95779800	0.98668000
H	-1.90589000	1.22333800	-0.38611400
H	-1.63338100	-0.16511900	0.64371500
C	-2.31141600	-0.86620800	-1.83974500
H	-2.78112200	-1.31889300	-2.71116100
H	-1.76184700	0.01126700	-2.20062900
C	-4.68760600	-2.73418100	-1.06664100
C	-3.54917900	-3.69465500	-1.51653500
H	-3.96591200	-4.41155800	-2.23417300
H	-2.72533200	-3.19260900	-2.01176200
H	-3.16472000	-4.28408500	-0.68346100
C	-5.76713300	-3.71964800	-0.52518000
H	-5.43678600	-4.25625800	0.36015500
H	-5.94322900	-4.47355300	-1.30160300
H	-6.72908700	-3.25272300	-0.32138400
C	-5.30004200	-2.17638500	-2.39094400
H	-4.57680300	-1.65320500	-3.01438900
H	-5.65848900	-3.03249500	-2.97573900
H	-6.15182600	-1.51866700	-2.23124500
C	-5.46376100	-0.94040500	0.85934400
C	-6.71447600	-0.59117400	-0.00886800
H	-7.45386300	-0.12037700	0.65072600
H	-6.50321900	0.11537000	-0.80872800
H	-7.19595700	-1.46546400	-0.44389500
C	-5.99233000	-1.89039600	1.97245900
H	-6.34613100	-2.84509300	1.59907500
H	-6.84233000	-1.40423100	2.46618000
H	-5.24457300	-2.06042700	2.74779800
C	-5.16138400	0.36967000	1.64804900
H	-4.94557800	1.22737400	1.01345900
H	-6.06063300	0.62311600	2.22191900
H	-1.57150500	-1.55262500	-1.43693700
H	-4.35682000	0.24786500	2.36828700
H	-0.39408400	-1.47594900	2.72724500
H	-1.24205800	-5.00477000	1.17239500
H	-2.14207700	0.24928000	2.67424400
C	-3.22465700	-4.37217200	1.77488100
C	-4.40982700	-4.86226800	2.36621700
C	-4.57140300	-6.20616600	2.71351100
C	-3.54007200	-7.12235200	2.48711900
C	-2.34585700	-6.66568900	1.92635900
C	-2.19326400	-5.31650000	1.58614400
H	-5.22869100	-4.18868300	2.58129900
H	-5.50580200	-6.53377200	3.16549100
H	-3.66286400	-8.17030100	2.75269000

H	-1.52177200	-7.35599000	1.75569900
C	-1.00227100	-2.52624200	0.93258300
C	-0.46987700	-3.19252800	-0.19308500
C	0.90421600	-3.26943400	-0.43533000
C	1.81091300	-2.68216900	0.45196500
C	1.31852600	-2.03649600	1.58766100
C	-0.05971500	-1.96942100	1.82287600
H	-1.12967900	-3.68072200	-0.89799200
H	1.26368600	-3.79352900	-1.31896900
H	2.88145300	-2.73638400	0.26539300
H	2.00486200	-1.58598500	2.30232600
C	-2.84268200	-1.73909700	3.17994800
C	-3.12184300	-2.56119600	4.29232700
C	-3.01945400	-2.09535300	5.60832600
C	-2.61554700	-0.78364500	5.86416000
C	-2.30434700	0.04804800	4.78448600
C	-2.41433100	-0.42599600	3.47452700
H	-3.41973000	-3.59186400	4.14338200
H	-3.25142800	-2.76754800	6.43240400
H	-2.53477000	-0.41765000	6.88559000
H	-1.97081500	1.06948300	4.95807800

(*t*-Bu)$_3$C–Si(*t*-Bu)$_3$
E = -9508.26
H = -9032.10
N_{imag} = 0
S = 1

H	-1.83265900	-0.39950700	3.47007900
C	-1.64919400	-3.31910800	2.56222500
C	-2.22150700	-2.58114700	3.81489100
C	-3.51087700	-3.17164000	4.46638600
H	-3.69537000	-2.61007900	5.38956900
H	-4.38654900	-3.03740600	3.82898100
H	-3.43558600	-4.22309000	4.74150200
C	-1.16557900	-2.57955600	4.96972400
H	-1.01971800	-3.59550600	5.35252300
H	-1.52291000	-1.95412400	5.79877700
H	-0.19070200	-2.20099700	4.66270100
C	-2.65449700	-1.11111500	3.47607000
H	-3.14774500	-1.07872900	2.49829900
H	-3.37434800	-0.76317000	4.22622600
C	-2.13832600	-4.76473800	2.22699400
C	-3.69001900	-4.81426200	1.99702200
H	-3.94152500	-5.70613800	1.41109600
H	-4.27062900	-4.86272200	2.91487200
H	-4.01524800	-3.93668100	1.42742200
C	-1.58264300	-5.42496800	0.92656500
H	-1.97545900	-4.94653400	0.02775300
H	-1.92966000	-6.46472700	0.91579600
H	-0.49531500	-5.45024600	0.86338300
C	-1.73086200	-5.75159300	3.37092600
H	-2.04160500	-5.41722100	4.36066200
H	-2.18407800	-6.73571400	3.19121100
H	-0.64322000	-5.88022800	3.39222700
C	-0.39994300	-2.73658400	1.82653200
C	0.87365300	-3.57480500	2.17836300
H	1.71991100	-3.24014900	1.56351400
H	1.14393200	-3.43172800	3.23023900

H	0.74522100	-4.64470800	2.01476700
C	-0.60840500	-2.69583000	0.27153600
H	-0.41969400	-3.64495000	-0.22384200
H	0.07873300	-1.96291600	-0.16741400
H	-1.63057900	-2.37949900	0.03601700
C	0.00078400	-1.26393300	2.15265100
H	0.15183500	-1.06246200	3.21262600
H	0.95427700	-1.06717000	1.64885600
H	-0.72343800	-0.54864200	1.75904800
H	-5.04126700	0.37685200	2.19299700
Si	-4.69510800	-1.29259000	-0.39061600
C	-3.73184100	0.24834300	-1.18311600
C	-4.70570900	1.26023000	-1.84291800
H	-4.12979300	2.09050300	-2.28159800
H	-5.29129200	0.80310000	-2.64747100
H	-5.40441000	1.69373100	-1.12127600
C	-2.91922500	0.97553900	-0.07801500
H	-3.55055000	1.38233100	0.71488800
H	-2.36365400	1.81387800	-0.52521900
H	-2.18998700	0.30239900	0.38444800
C	-2.71168200	-0.21034800	-2.25569100
H	-3.19369700	-0.65985000	-3.12836200
H	-2.14514000	0.66465900	-2.60861800
C	-5.15869100	-2.68221500	-1.72637800
C	-3.87595900	-3.45436300	-2.13719900
H	-4.13960300	-4.24289200	-2.85842800
H	-3.12666100	-2.81339300	-2.60671000
H	-3.41084800	-3.93735400	-1.27165700
C	-6.13896400	-3.72937200	-1.13966500
H	-5.73889700	-4.20301000	-0.23715800
H	-6.30380300	-4.52267400	-1.88438500
H	-7.11585400	-3.30086100	-0.89861300
C	-5.80812000	-2.06901700	-2.99530000
H	-5.13543700	-1.37870400	-3.51285600
H	-6.06054600	-2.87408900	-3.70351800
H	-6.73302700	-1.53007300	-2.76452900
C	-6.24223300	-0.74542700	0.72161700
C	-7.49740300	-0.45004900	-0.14163200
H	-8.32690800	-0.13731200	0.51226500
H	-7.32157000	0.35678100	-0.86086100
H	-7.83448000	-1.33008700	-0.69735900
C	-6.57839600	-1.87407200	1.73333300
H	-6.86567200	-2.80856500	1.24660700
H	-7.41936900	-1.55656100	2.36849700
H	-5.72713400	-2.08402000	2.38912300
C	-5.92129400	0.51957800	1.55730800
H	-5.75557500	1.40309100	0.93448000
H	-6.77416100	0.73907200	2.21726400
H	-1.99044700	-0.92893300	-1.85280100

$H_3Ge–GeH_3$
E = -627.75
H = -595.80
N_{imag} = 0
S = 0

Ge	0.00000000	0.00000000	-1.22024200
H	0.00000000	-1.44267800	-1.75991300
H	1.24939600	0.72133900	-1.75991300

H	-1.24939600	0.72133900	-1.75991300
Ge	0.00000000	0.00000000	1.22024200
H	0.00000000	1.44267800	1.75991300
H	1.24939600	-0.72133900	1.75991300
H	-1.24939600	-0.72133900	1.75991300

Ph_3Ge–$GePh_3$
E = -9767.89
H = -9414.51
N_{imag} = 0
S = 0

Ge	0.00011900	0.00079400	1.21819800
C	1.87187900	0.02881500	1.83300100
C	2.74582500	1.02474800	1.36086900
C	4.09421700	1.02198200	1.73204100
C	4.58715700	0.01984400	2.57661500
C	3.72563400	-0.97516600	3.05107500
C	2.37556700	-0.97005400	2.68036000
C	-0.96067000	1.60775600	1.83243800
C	-0.35121900	2.53942000	2.68682200
C	-1.02365800	3.70924800	3.06034400
C	-2.31418500	3.95981200	2.58166400
C	-2.93170600	3.03602500	1.72970200
C	-2.25812400	1.86875700	1.35588100
C	-0.91271300	-1.63339800	1.83383400
C	-2.02612900	-1.56839700	2.68561200
C	-2.70613900	-2.73389400	3.05888100
C	-2.27919900	-3.97816200	2.58258900
H	2.37943800	1.80031700	0.69185100
H	4.76011200	1.79721000	1.35817400
H	5.63698000	0.01461600	2.86217700
H	4.10432400	-1.75623400	3.70760400
H	1.71504200	-1.75223000	3.04849700
H	0.65482100	2.35679700	3.05851700
H	-0.53959700	4.42464700	3.72245600
H	-2.83599900	4.87007200	2.86948400
H	-3.93442100	3.22676200	1.35240400
H	-2.74376100	1.16661000	0.68174900
H	-2.37016700	-0.60473700	3.05520900
H	-3.56921000	-2.66992700	3.71882200
H	-2.80905000	-4.88385700	2.87011900
H	-0.83337800	-5.01894900	1.35789100
H	0.36079300	-2.96075400	0.68737100
C	-1.16853200	-4.05412700	1.73334400
C	-0.49131800	-2.88887400	1.35972600
Ge	-0.00011900	-0.00079400	-1.21819800
C	-1.87187900	-0.02881500	-1.83300100
C	-2.74582500	-1.02474800	-1.36086900
C	-4.09421700	-1.02198200	-1.73204100
C	-4.58715700	-0.01984400	-2.57661500
C	-3.72563400	0.97516600	-3.05107500
C	-2.37556700	0.97005400	-2.68036000
C	0.91271300	1.63339800	-1.83383400
C	0.49131800	2.88887400	-1.35972600
C	1.16853200	4.05412700	-1.73334400
C	2.27919900	3.97816200	-2.58258900
C	2.70613900	2.73389400	-3.05888100
C	2.02612900	1.56839700	-2.68561200

C	0.96067000	-1.60775600	-1.83243800
C	2.25812400	-1.86875700	-1.35588100
C	2.93170600	-3.03602500	-1.72970200
C	2.31418500	-3.95981200	-2.58166400
C	1.02365800	-3.70924800	-3.06034400
C	0.35121900	-2.53942000	-2.68682200
H	-2.37943800	-1.80031700	-0.69185100
H	-4.76011200	-1.79721000	-1.35817400
H	-5.63698000	-0.01461600	-2.86217700
H	-4.10432400	1.75623400	-3.70760400
H	-1.71504200	1.75223000	-3.04849700
H	-0.36079300	2.96075400	-0.68737100
H	0.83337800	5.01894900	-1.35789100
H	2.80905000	4.88385700	-2.87011900
H	3.56921000	2.66992700	-3.71882200
H	2.37016700	0.60473700	-3.05520900
H	2.74376100	-1.16661000	-0.68174900
H	3.93442100	-3.22676200	-1.35240400
H	2.83599900	-4.87007200	-2.86948400
H	0.53959700	-4.42464700	-3.72245600
H	-0.65482100	-2.35679700	-3.05851700

(*t*-Bu)$_3$Ge–Ge(*t*-Bu)$_3$
E = -9429.75
H = -8955.29
N_{imag} = 0
S = 0

H	-3.24151100	-0.07191000	-1.53279900
Ge	0.00000000	0.00000000	-1.36305200
C	-1.55295200	1.20063200	-2.14745100
C	-1.19901600	2.69259900	-1.99522400
H	-2.03015900	3.30368600	-2.38186000
H	-1.04898500	2.97763500	-0.95154400
H	-0.30430900	2.97260400	-2.55651700
C	-1.78952500	0.90944000	-3.64617000
H	-0.89720700	1.04853000	-4.25884700
H	-2.55702600	1.60500500	-4.02219000
H	-2.16658000	-0.10211800	-3.81572200
C	-2.88344100	0.95323100	-1.41556000
H	-2.81481800	1.16341900	-0.34871200
H	-3.65505200	1.61975800	-1.83343200
C	1.81625400	0.74458000	-2.14745100
C	2.26724200	2.02051700	-1.41556000
H	3.23027800	2.35548900	-1.83343200
H	1.55848000	2.84318600	-1.53279900
H	2.41495900	1.85599500	-0.34871200
C	2.93136700	-0.30792200	-1.99522400
H	3.10320000	-0.58037000	-0.95154400
H	3.87615600	0.10632600	-2.38186000
H	2.72650500	-1.22276300	-2.55651700
C	1.68236100	1.09505400	-3.64617000
H	0.99485300	1.92737300	-3.81572200
H	2.66848800	1.41194700	-4.02219000
H	1.35665700	0.25273900	-4.25884700
C	-0.26330200	-1.94521200	-2.14745100
C	0.10716400	-2.00449400	-3.64617000
H	-0.11146200	-3.01695200	-4.02219000
H	-0.45945000	-1.30126900	-4.25884700

H	1.17172700	-1.82525500	-3.81572200
C	0.61619800	-2.97374800	-1.41556000
H	1.68303200	-2.77127600	-1.53279900
H	0.42477500	-3.97524700	-1.83343200
H	0.39985900	-3.01941300	-0.34871200
C	-1.73235200	-2.38467700	-1.99522400
H	-2.42219600	-1.74984100	-2.55651700
H	-1.84599700	-3.41001300	-2.38186000
H	-2.05421500	-2.39726500	-0.95154400
H	-2.05421500	2.39726500	0.95154400
Ge	0.00000000	0.00000000	1.36305200
C	-1.55295200	-1.20063200	2.14745100
C	-1.78952500	-0.90944000	3.64617000
H	-2.55702600	-1.60500500	4.02219000
H	-0.89720700	-1.04853000	4.25884700
H	-2.16658000	0.10211800	3.81572200
C	-2.88344100	-0.95323100	1.41556000
H	-3.24151100	0.07191000	1.53279900
H	-3.65505200	-1.61975800	1.83343200
H	-2.81481800	-1.16341900	0.34871200
C	-1.19901600	-2.69259900	1.99522400
H	-0.30430900	-2.97260400	2.55651700
H	-2.03015900	-3.30368600	2.38186000
C	1.81625400	-0.74458000	2.14745100
C	2.26724200	-2.02051700	1.41556000
H	3.23027800	-2.35548900	1.83343200
H	1.55848000	-2.84318600	1.53279900
H	2.41495900	-1.85599500	0.34871200
C	2.93136700	0.30792200	1.99522400
H	3.10320000	0.58037000	0.95154400
H	3.87615600	-0.10632600	2.38186000
H	2.72650500	1.22276300	2.55651700
C	1.68236100	-1.09505400	3.64617000
H	0.99485300	-1.92737300	3.81572200
H	2.66848800	-1.41194700	4.02219000
H	1.35665700	-0.25273900	4.25884700
C	-0.26330200	1.94521200	2.14745100
C	0.10716400	2.00449400	3.64617000
H	-0.11146200	3.01695200	4.02219000
H	-0.45945000	1.30126900	4.25884700
H	1.17172700	1.82525500	3.81572200
C	0.61619800	2.97374800	1.41556000
H	1.68303200	2.77127600	1.53279900
H	0.42477500	3.97524700	1.83343200
H	0.39985900	3.01941300	0.34871200
C	-1.73235200	2.38467700	1.99522400
H	-2.42219600	1.74984100	2.55651700
H	-1.84599700	3.41001300	2.38186000
H	-1.04898500	-2.97763500	0.95154400

$H_3Sn–SnH_3$
E = -569.60
H = -541.02
N_{imag} = 0
S = 0

Sn	0.00000000	0.00000000	-1.40862200
H	0.00000000	-1.61512200	-2.02481100
H	1.39873700	0.80756100	-2.02481100

H	-1.39873700	0.80756100	-2.02481100
Sn	0.00000000	0.00000000	1.40862200
H	0.00000000	1.61512200	2.02481100
H	1.39873700	-0.80756100	2.02481100
H	-1.39873700	-0.80756100	2.02481100

$Ph_3Sn–SnPh_3$
E = -9700.06
H = -9350.40
N_{imag} = 0
S = 0

Sn	-0.00099600	0.00166300	1.39306800
C	2.04977100	0.04073100	2.11079000
C	2.96514800	0.95575800	1.56176800
C	4.30030900	0.96558500	1.98204100
C	4.73582700	0.05596100	2.95291500
C	3.83348800	-0.86185000	3.50182600
C	2.49740600	-0.86976900	3.08124200
C	-1.06040300	1.75917800	2.10775000
C	-0.51028200	2.58660900	3.09968100
C	-1.18732300	3.73787700	3.52189600
C	-2.42084200	4.07402600	2.95323000
C	-2.97595100	3.25782100	1.96059200
C	-2.29786700	2.10810500	1.53894900
C	-0.99129600	-1.79350300	2.11315100
C	-1.98851500	-1.72758600	3.09924200
C	-2.64709200	-2.88852100	3.52406800
C	-2.31592400	-4.12723000	2.96382600
H	2.64509500	1.66326400	0.79788400
H	4.99954700	1.67875100	1.54952200
H	5.77414100	0.06104700	3.27791100
H	4.16925100	-1.57260700	4.25477600
H	1.80677700	-1.59412300	3.51015100
H	0.45272600	2.34023200	3.54412900
H	-0.75106400	4.37197900	4.29159100
H	-2.94602700	4.96921300	3.27965700
H	-3.93330800	3.51747400	1.51265700
H	-2.73923000	1.48905800	0.75889100
H	-2.26099600	-0.76857100	3.53705500
H	-3.41875100	-2.82511100	4.28916200
H	-2.82863000	-5.02892900	3.29216700
H	-1.06735900	-5.16398600	1.53603300
H	0.09224600	-3.11746100	0.77750900
C	-1.32566300	-4.20316100	1.97728800
C	-0.66896400	-3.04205400	1.55300000
Sn	0.00099600	-0.00166300	-1.39306800
C	-2.04977100	-0.04073100	-2.11079000
C	-2.96514800	-0.95575800	-1.56176800
C	-4.30030900	-0.96558500	-1.98204100
C	-4.73582700	-0.05596100	-2.95291500
C	-3.83348800	0.86185000	-3.50182600
C	-2.49740600	0.86976900	-3.08124200
C	0.99129600	1.79350300	-2.11315100
C	0.66896400	3.04205400	-1.55300000
C	1.32566300	4.20316100	-1.97728800
C	2.31592400	4.12723000	-2.96382600
C	2.64709200	2.88852100	-3.52406800
C	1.98851500	1.72758600	-3.09924200

C	1.06040300	-1.75917800	-2.10775000
C	2.29786700	-2.10810500	-1.53894900
C	2.97595100	-3.25782100	-1.96059200
C	2.42084200	-4.07402600	-2.95323000
C	1.18732300	-3.73787700	-3.52189600
C	0.51028200	-2.58660900	-3.09968100
H	-2.64509500	-1.66326400	-0.79788400
H	-4.99954700	-1.67875100	-1.54952200
H	-5.77414100	-0.06104700	-3.27791100
H	-4.16925100	1.57260700	-4.25477600
H	-1.80677700	1.59412300	-3.51015100
H	-0.09224600	3.11746100	-0.77750900
H	1.06735900	5.16398600	-1.53603300
H	2.82863000	5.02892900	-3.29216700
H	3.41875100	2.82511100	-4.28916200
H	2.26099600	0.76857100	-3.53705500
H	2.73923000	-1.48905800	-0.75889100
H	3.93330800	-3.51747400	-1.51265700
H	2.94602700	-4.96921300	-3.27965700
H	0.75106400	-4.37197900	-4.29159100
H	-0.45272600	-2.34023200	-3.54412900

(*t*-Bu)$_3$Sn–Sn(*t*-Bu)$_3$
E = -9393.27
H = -8921.99
N_{imag} = 0
S = 0

H	-3.31073800	-0.01395000	-1.61904900
Sn	0.00000000	0.00000000	-1.45993100
C	-1.68259900	1.30525800	-2.28684500
C	-1.30138400	2.78553800	-2.11738400
H	-2.11655600	3.42644200	-2.49460900
H	-1.14012100	3.05186800	-1.06751700
H	-0.39678900	3.04920200	-2.67515500
C	-1.90203200	0.99692600	-3.77914400
H	-1.00320000	1.16563400	-4.38034700
H	-2.69279400	1.65488700	-4.17871800
H	-2.22963900	-0.03495400	-3.94218300
C	-2.98186600	1.02495500	-1.51533300
H	-2.88065200	1.23673900	-0.44708600
H	-3.79156400	1.66693200	-1.90265900
C	1.97168600	0.80454500	-2.28684500
C	2.37857000	2.06989400	-1.51533300
H	3.33938700	2.45012500	-1.90265900
H	1.64328800	2.87415900	-1.61904900
H	2.51137300	1.87634900	-0.44708600
C	3.06303900	-0.26573700	-2.11738400
H	3.21305600	-0.53856100	-1.06751700
H	4.02566400	0.11977000	-2.49460900
H	2.83908100	-1.18097100	-2.67515500
C	1.81437900	1.14874500	-3.77914400
H	1.08454800	1.94840100	-3.94218300
H	2.77957100	1.50458500	-4.17871800
H	1.51106900	0.28597900	-4.38034700
C	-0.28908700	-2.10980200	-2.28684500
C	0.08765300	-2.14567100	-3.77914400
H	-0.08677700	-3.15947100	-4.17871800
H	-0.50786900	-1.45161400	-4.38034700

H	1.14509100	-1.91344700	-3.94218300
C	0.60329600	-3.09484900	-1.51533300
H	1.66745000	-2.86020800	-1.61904900
H	0.45217700	-4.11705700	-1.90265900
H	0.36927900	-3.11308700	-0.44708600
C	-1.76165500	-2.51980000	-2.11738400
H	-2.44229200	-1.86823100	-2.67515500
H	-1.90910800	-3.54621300	-2.49460900
H	-2.07293500	-2.51330800	-1.06751700
H	-2.07293500	2.51330800	1.06751700
Sn	0.00000000	0.00000000	1.45993100
C	-1.68259900	-1.30525800	2.28684500
C	-1.90203200	-0.99692600	3.77914400
H	-2.69279400	-1.65488700	4.17871800
H	-1.00320000	-1.16563400	4.38034700
H	-2.22963900	0.03495400	3.94218300
C	-2.98186600	-1.02495500	1.51533300
H	-3.31073800	0.01395000	1.61904900
H	-3.79156400	-1.66693200	1.90265900
H	-2.88065200	-1.23673900	0.44708600
C	-1.30138400	-2.78553800	2.11738400
H	-0.39678900	-3.04920200	2.67515500
H	-2.11655600	-3.42644200	2.49460900
C	1.97168600	-0.80454500	2.28684500
C	2.37857000	-2.06989400	1.51533300
H	3.33938700	-2.45012500	1.90265900
H	1.64328800	-2.87415900	1.61904900
H	2.51137300	-1.87634900	0.44708600
C	3.06303900	0.26573700	2.11738400
H	3.21305600	0.53856100	1.06751700
H	4.02566400	-0.11977000	2.49460900
H	2.83908100	1.18097100	2.67515500
C	1.81437900	-1.14874500	3.77914400
H	1.08454800	-1.94840100	3.94218300
H	2.77957100	-1.50458500	4.17871800
H	1.51106900	-0.28597900	4.38034700
C	-0.28908700	2.10980200	2.28684500
C	0.08765300	2.14567100	3.77914400
H	-0.08677700	3.15947100	4.17871800
H	-0.50786900	1.45161400	4.38034700
H	1.14509100	1.91344700	3.94218300
C	0.60329600	3.09484900	1.51533300
H	1.66745000	2.86020800	1.61904900
H	0.45217700	4.11705700	1.90265900
H	0.36927900	3.11308700	0.44708600
C	-1.76165500	2.51980000	2.11738400
H	-2.44229200	1.86823100	2.67515500
H	-1.90910800	3.54621300	2.49460900
H	-1.14012100	-3.05186800	1.06751700

H_3Pb–PbH_3
E = -511.76
H = -484.95
N_{imag} = 0
S = 0

Pb	0.00000000	0.00000000	-1.46782100
H	0.00000000	-1.67267000	-2.11022900
H	1.44857400	0.83633500	-2.11022900

H	-1.44857400	0.83633500	-2.11022900
Pb	0.00000000	0.00000000	1.46782100
H	0.00000000	1.67267000	2.11022900
H	1.44857400	-0.83633500	2.11022900
H	-1.44857400	-0.83633500	2.11022900

$Ph_3Pb–PbPh_3$
E = -9639.24
H = -9289.84
N_{imag} = 0
S = 0

Pb	-0.02870700	0.00590000	1.42603000
C	2.06454200	0.45136300	2.18281000
C	2.84669500	1.42740700	1.54928600
C	4.14674600	1.68776100	2.00186100
C	4.66890200	0.97246700	3.08581000
C	3.89168600	-0.00604200	3.71568000
C	2.59133800	-0.26871700	3.26288900
C	-1.44709700	1.56870100	2.26040300
C	-1.06744000	2.35312800	3.35719200
C	-1.94237500	3.32447500	3.86305100
C	-3.19530600	3.51641600	3.27014400
C	-3.57338500	2.73834700	2.16977400
C	-2.70036200	1.76629000	1.66412200
C	-0.64944700	-2.01951400	2.24068400
C	-1.51760400	-2.10196800	3.33703500
C	-1.90633000	-3.35405400	3.83321400
C	-1.43221900	-4.52478700	3.23083800
H	2.45949700	1.98474100	0.69814500
H	4.75037900	2.44524700	1.50523100
H	5.67907800	1.17427000	3.43585500
H	4.29628800	-0.56712600	4.55626900
H	1.99881500	-1.03968000	3.75325500
H	-0.09090600	2.21862200	3.81999000
H	-1.64359100	3.93083100	4.71636200
H	-3.87312800	4.27199600	3.66169400
H	-4.54472000	2.88886300	1.70212700
H	-3.00492300	1.17623400	0.80126800
H	-1.89957100	-1.19708000	3.80739100
H	-2.57984500	-3.41324000	4.68643300
H	-1.73596700	-5.49638300	3.61479400
H	-0.20408300	-5.35239700	1.65590200
H	0.48301200	-3.14766100	0.77257500
C	-0.57030000	-4.44405300	2.13091600
C	-0.17976100	-3.19338900	1.63491500
Pb	-0.03817200	0.01487200	-1.49431200
C	-2.12320100	0.36714500	-2.31699700
C	-3.20205500	-0.38451800	-1.83115100
C	-4.49932200	-0.14065900	-2.30052300
C	-4.72211400	0.85930000	-3.25426300
C	-3.64730200	1.61292200	-3.73880900
C	-2.34916500	1.36839200	-3.27001100
C	1.33830200	1.64747000	-2.26361800
C	1.16193200	2.97113400	-1.83679000
C	2.05014700	3.97087200	-2.25422500
C	3.12102900	3.64886600	-3.09613100
C	3.30062600	2.32813200	-3.52201600
C	2.41120500	1.32816900	-3.10546300

C	0.72777500	-1.98030500	-2.25862700
C	1.92891600	-2.50195400	-1.75789600
C	2.38224600	-3.75856100	-2.17993600
C	1.63299100	-4.49980400	-3.10104000
C	0.43265900	-3.98255600	-3.60074100
C	-0.02026900	-2.72479100	-3.17936900
H	-3.04416200	-1.16254000	-1.08531000
H	-5.33276600	-0.72888300	-1.92079000
H	-5.72962200	1.05114800	-3.61705400
H	-3.81768000	2.39264600	-4.47903600
H	-1.52116000	1.96510200	-3.64944300
H	0.33746300	3.23491800	-1.17566600
H	1.90709100	4.99702700	-1.92048300
H	3.81319300	4.42413400	-3.41768500
H	4.13347700	2.07421600	-4.17533900
H	2.56424400	0.30260200	-3.43776100
H	2.51906800	-1.93913700	-1.03569500
H	3.31631100	-4.15746600	-1.78848200
H	1.98270400	-5.47714100	-3.42665400
H	-0.15329500	-4.55759500	-4.31555000
H	-0.95908400	-2.33507200	-3.56980900

(*t*-Bu)$_3$Pb–Pb(*t*-Bu)$_3$

E = -9351.13

H = -8881.59

N_{imag} = 0

S = 0

H	-3.35222600	0.02012100	-1.67872900
Pb	0.00000000	0.00000000	-1.51695800
C	-1.76895300	1.37163000	-2.36893400
C	-1.35837300	2.83493500	-2.18261900
H	-2.16313200	3.50122600	-2.54241400
H	-1.18197300	3.08146000	-1.12958300
H	-0.45295100	3.08736300	-2.74538100
C	-1.97159800	1.04459300	-3.85243400
H	-1.06974500	1.22609500	-4.44694100
H	-2.77275900	1.68133800	-4.26966800
H	-2.27566300	0.00347100	-4.00650200
C	-3.03507700	1.06250300	-1.56689600
H	-2.90305000	1.25841900	-0.49766900
H	-3.86775700	1.69941500	-1.91628100
C	2.07234300	0.84614300	-2.36893400
C	2.43769300	2.09720200	-1.56689600
H	3.40561500	2.49986800	-1.91628100
H	1.69353800	2.89305300	-1.67872900
H	2.54134800	1.88490500	-0.49766900
C	3.13431200	-0.24108200	-2.18261900
H	3.25960900	-0.51711200	-1.12958300
H	4.11371700	0.12271400	-2.54241400
H	2.90021000	-1.15141500	-2.74538100
C	1.89044300	1.18515700	-3.85243400
H	1.14083800	1.96904600	-4.00650200
H	2.84246000	1.56061100	-4.26966800
H	1.59670200	0.31337900	-4.44694100
C	-0.30339000	-2.21777300	-2.36893400
C	0.08115500	-2.22975000	-3.85243400
H	-0.06970200	-3.24194800	-4.26966800
H	-0.52695700	-1.53947400	-4.44694100

H	1.13482500	-1.97251800	-4.00650200
C	0.59738400	-3.15970500	-1.56689600
H	1.65868800	-2.91317300	-1.67872900
H	0.46214200	-4.19928300	-1.91628100
H	0.36170200	-3.14332400	-0.49766900
C	-1.77593900	-2.59385300	-2.18261900
H	-2.44725900	-1.93594800	-2.74538100
H	-1.95058500	-3.62394000	-2.54241400
H	-2.07763700	-2.56434900	-1.12958300
H	-2.07763700	2.56434900	1.12958300
Pb	0.00000000	0.00000000	1.51695800
C	-1.76895300	-1.37163000	2.36893400
C	-1.97159800	-1.04459300	3.85243400
H	-2.77275900	-1.68133800	4.26966800
H	-1.06974500	-1.22609500	4.44694100
H	-2.27566300	-0.00347100	4.00650200
C	-3.03507700	-1.06250300	1.56689600
H	-3.35222600	-0.02012100	1.67872900
H	-3.86775700	-1.69941500	1.91628100
H	-2.90305000	-1.25841900	0.49766900
C	-1.35837300	-2.83493500	2.18261900
H	-0.45295100	-3.08736300	2.74538100
H	-2.16313200	-3.50122600	2.54241400
C	2.07234300	-0.84614300	2.36893400
C	2.43769300	-2.09720200	1.56689600
H	3.40561500	-2.49986800	1.91628100
H	1.69353800	-2.89305300	1.67872900
H	2.54134800	-1.88490500	0.49766900
C	3.13431200	0.24108200	2.18261900
H	3.25960900	0.51711200	1.12958300
H	4.11371700	-0.12271400	2.54241400
H	2.90021000	1.15141500	2.74538100
C	1.89044300	-1.18515700	3.85243400
H	1.14083800	-1.96904600	4.00650200
H	2.84246000	-1.56061100	4.26966800
H	1.59670200	-0.31337900	4.44694100
C	-0.30339000	2.21777300	2.36893400
C	0.08115500	2.22975000	3.85243400
H	-0.06970200	3.24194800	4.26966800
H	-0.52695700	1.53947400	4.44694100
H	1.13482500	1.97251800	4.00650200
C	0.59738400	3.15970500	1.56689600
H	1.65868800	2.91317300	1.67872900
H	0.46214200	4.19928300	1.91628100
H	0.36170200	3.14332400	0.49766900
C	-1.77593900	2.59385300	2.18261900
H	-2.44725900	1.93594800	2.74538100
H	-1.95058500	3.62394000	2.54241400
H	-1.18197300	-3.08146000	1.12958300

Table S9. Cartesian coordinates (in Å), energies (electronic E and enthalpy H, in kcal mol^{-1}), number of imaginary frequencies (N_{imag}), and total spin number (S) of the $R_3A^{•}$ radical species studied herein, computed at (U)BLYP-D3(BJ)/TZ2P for A = C, Si and at ZORA-(U)BLYP-D3(BJ)/TZ2P for A = Ge, Sn, Pb.

$H_3C^{•}$
E = -404.37
H = -383.63
N_{imag} = 0
S = 1/2

C	0.000000	0.000000	1.066517
H	0.541956	0.938695	1.066517
H	0.541956	-0.938695	1.066517
H	-1.083912	0.000000	1.066517

$Me_3C^{•}$
E = -1513.36
H = -1437.92
N_{imag} = 0
S = 1/2

H	1.744333	-1.247408	0.473511
H	-1.948620	0.895333	0.473592
H	-1.952441	-0.886900	0.473745
H	-1.775451	0.003695	-1.043232
C	-0.000013	0.000005	0.201232
C	-1.487590	0.003187	0.029132
C	0.741023	-1.289859	0.028992
C	0.746559	1.286678	0.029072
H	0.208040	2.134355	0.473439
H	0.891179	1.535552	-1.043292
H	1.749606	1.239942	0.473744
H	0.884420	-1.539363	-1.043393
H	0.198955	-2.135218	0.473457

$PhMe_2C^{•}$
E = -2673.65
H = -2563.27
N_{imag} = 0
S = 1/2

C	1.258332	-0.748489	0.002977
C	1.547066	-0.033392	-1.290502
C	0.695024	-2.059244	-0.000183
C	1.576894	-0.052471	1.299867
H	-0.339149	-4.540243	2.149065
H	-0.388737	-4.508491	-2.161371
H	-0.860860	-5.681087	-0.008966
H	2.299320	-0.622044	1.905794
H	0.681964	0.073663	1.929669
H	2.001400	0.940457	1.124034
H	0.638418	0.099571	-1.898861
H	2.257272	-0.592708	-1.920039
H	1.973409	0.957889	-1.109879
H	0.578734	-2.256116	-2.169951

C	0.411799	-2.760101	1.214965
C	-0.136834	-4.037078	1.205235
C	-0.431657	-4.681999	-0.006545
C	-0.164680	-4.019226	-1.215147
C	0.383813	-2.742170	-1.218699
H	0.628550	-2.288019	2.168535

$Ph_2MeC^•$
E = -3827.89
H = -3682.83
N_{imag} = 0
S = 1/2

C	2.262273	0.461185	0.339592
H	-0.056387	1.942483	2.360697
C	1.554514	2.954775	1.376745
H	1.279902	3.922347	1.792405
C	0.324251	-0.628213	1.510143
C	0.791949	1.831944	1.690910
H	3.261430	3.723725	0.288272
C	3.012931	1.588821	0.017500
H	3.863693	1.491706	-0.654224
H	2.522369	-0.497125	-0.099349
C	2.671442	2.844930	0.537815
H	-0.969479	-3.019346	1.312548
C	0.897053	-1.948687	1.644426
C	2.274082	-2.151593	1.955457
C	2.801890	-3.427549	2.119569
C	1.986965	-4.561878	1.984484
C	0.625453	-4.391829	1.698666
C	0.087377	-3.117412	1.540947
H	2.915179	-1.288728	2.103647
H	3.854917	-3.542516	2.369472
H	2.403516	-5.558372	2.111251
H	-0.020428	-5.261887	1.596607
C	1.127952	0.543580	1.193651
C	-1.169907	-0.473247	1.664174
H	-1.543474	-0.957429	2.576520
H	-1.697843	-0.943904	0.819022
H	-1.473238	0.575347	1.674358

$Ph_3C^•$
E = -4984.21
H = -4804.71
N_{imag} = 0
S = 1/2

C	-1.241183	0.005393	0.879499
C	-0.675301	-1.021109	1.751226
C	-0.104631	-2.207498	1.220764
C	0.438109	-3.183347	2.053648
C	0.423928	-3.017595	3.445475
C	-0.138515	-1.855791	3.992029
C	-0.674211	-0.872894	3.162837
C	-1.144036	1.414585	1.251276
C	-0.019041	1.916440	1.956457

C	0.073494	3.261817	2.305645
C	-0.954990	4.154496	1.973653
C	-2.077806	3.680343	1.281312
C	-2.170779	2.337684	0.921943
C	-1.904218	-0.377908	-0.364478
C	-1.837734	0.443257	-1.520227
C	-2.466917	0.073097	-2.706700
C	-3.192980	-1.123530	-2.782625
C	-3.276068	-1.948229	-1.652238
C	-2.640927	-1.586826	-0.466260
H	-0.072013	-2.340294	0.143253
H	0.883131	-4.075157	1.616754
H	0.844863	-3.782212	4.094373
H	-0.166577	-1.721258	5.071548
H	-1.122514	0.014721	3.599753
H	0.792524	1.238585	2.204624
H	0.955764	3.620366	2.832239
H	-0.882571	5.203843	2.250299
H	-2.888391	4.361112	1.028790
H	-3.053845	1.979653	0.400671
H	-1.264050	1.364654	-1.479574
H	-2.384723	0.715640	-3.581138
H	-3.686483	-1.409136	-3.708794
H	-3.847000	-2.873675	-1.695110
H	-2.727148	-2.226387	0.407391

(*t*-Bu)$_3$C$^•$
E = -4781.48
H = -4541.97
***N*$_{imag}$** = 0
S = 1/2

H	-4.85541300	-0.32233600	2.56773200
C	-4.54236100	-1.40435700	-0.23759900
C	-3.66604900	-0.12780000	-0.46588600
C	-4.54708000	1.04401000	-1.01383000
H	-3.94688600	1.96179700	-1.07083800
H	-4.90688400	0.81384700	-2.02213500
H	-5.41800200	1.25455900	-0.39305700
C	-2.93162900	0.33168200	0.84289400
H	-3.56242000	0.88616000	1.53245900
H	-2.09755900	0.99019100	0.57262800
H	-2.51579700	-0.53318500	1.37180300
C	-2.48936100	-0.24623700	-1.48414400
H	-2.78507900	-0.58746300	-2.47559600
H	-2.06052900	0.75528600	-1.60495100
C	-4.48974400	-2.56960000	-1.28125200
C	-3.04660700	-3.16785300	-1.43301100
H	-3.11756600	-4.16101600	-1.89231600
H	-2.38603000	-2.57527600	-2.06019900
H	-2.57695600	-3.28774300	-0.45028800
C	-5.34555100	-3.83804600	-0.97443100
H	-4.94960300	-4.39785300	-0.12389000
H	-5.28258400	-4.49362100	-1.85069500
H	-6.40241000	-3.63699900	-0.80273700
C	-5.00307800	-2.07386300	-2.67421700
H	-4.48557500	-1.18374900	-3.03207800
H	-4.86358900	-2.86558500	-3.42223200

H	-6.07169800	-1.83939400	-2.62741400
C	-5.70493800	-1.36177800	0.80945000
C	-7.08292100	-1.20443900	0.08410300
H	-7.89693900	-1.28107800	0.81714000
H	-7.15335400	-0.22266300	-0.39579600
H	-7.25450000	-1.95951300	-0.68312500
C	-5.73119400	-2.63897000	1.72134500
H	-6.18351400	-3.50834900	1.25180800
H	-6.31571500	-2.42249900	2.62345800
H	-4.71606100	-2.90404400	2.03740200
C	-5.67663300	-0.20510700	1.85680500
H	-5.62774800	0.79319300	1.42337000
H	-6.61001700	-0.26036100	2.42887900
H	-1.69365700	-0.89460500	-1.10998000

$H_3Si^{\bullet}$

E = -308.22

H = -292.78

N_{imag} = 0

S = 1/2

Si	0.000000	0.000000	0.779159
H	0.709576	1.229022	1.235391
H	0.709576	-1.229022	1.235391
H	-1.419152	0.000000	1.235391

$Me_3Si^{\bullet}$

E = -1436.01

H = -1363.97

N_{imag} = 0

S = 1/2

Si	1.500087	-0.130634	-0.588076
C	3.012777	-1.255190	-0.355614
C	2.033543	1.691674	-0.549090
C	0.226653	-0.463941	0.780918
H	3.761818	-1.085153	-1.138008
H	3.487361	-1.051921	0.617278
H	2.731097	-2.314551	-0.378362
H	1.177095	2.362355	-0.686197
H	2.496508	1.928006	0.421933
H	2.766772	1.910853	-1.334236
H	-0.100983	-1.510239	0.776504
H	0.669298	-0.252000	1.766929
H	-0.659825	0.170940	0.666022

$PhMe_2Si^{\bullet}$

E = -2586.53

H = -2479.44

N_{imag} = 0

S = 1/2

Si	1.352611	0.018632	0.004086
C	1.682091	-0.982786	-1.573639
C	1.733574	-1.004128	1.556233
H	3.647364	3.948063	2.147814
H	1.402748	-0.426746	-2.475439
H	2.749857	-1.237124	-1.647978
H	1.107877	-1.916616	-1.564261
H	4.183012	5.077201	-0.008328
H	3.518815	4.008786	-2.159703

H	2.329841	1.850260	-2.161647
H	1.165990	-1.942019	1.547600
H	2.804702	-1.251653	1.597782
H	1.474563	-0.464517	2.473964
C	2.300552	1.634838	-0.000694
C	2.683745	2.264446	1.206600
C	3.356963	3.488287	1.204837
C	3.660100	4.123190	-0.006172
C	3.285002	3.522233	-1.214445
C	2.611959	2.298416	-1.210487
H	2.459018	1.789179	2.159836

$Ph_2MeSi^{\bullet}$
E = -3736.27
H = -3594.14
N_{imag} = 0
S = 1/2

C	-0.008963	2.804154	-2.440791
H	-2.295355	0.823605	-4.016274
H	-2.799849	2.964446	-5.131418
Si	-0.310732	0.024891	-1.857747
H	-1.521348	5.021072	-4.544474
H	0.276891	4.906568	-2.821379
H	0.773378	2.777887	-1.683154
H	0.369685	-2.750466	-2.773669
C	-1.328026	-1.438264	-2.440384
C	-2.741252	-1.379866	-2.476941
C	-3.502142	-2.480555	-2.875009
C	-2.871641	-3.679106	-3.233980
C	-1.475539	-3.765866	-3.189914
C	-0.714623	-2.661282	-2.795934
H	-3.250444	-0.463939	-2.182205
H	-4.587849	-2.407987	-2.899446
C	1.551844	-0.330135	-1.891476
H	-3.464124	-4.538655	-3.540115
H	1.810522	-1.177294	-1.246491
H	2.115891	0.543098	-1.545544
H	1.880990	-0.558710	-2.915435
H	-0.978720	-4.694322	-3.465497
C	-0.721185	1.622861	-2.756110
C	-1.735718	1.712495	-3.736129
C	-2.019362	2.923576	-4.373679
C	-1.299119	4.078768	-4.048198
C	-0.288974	4.013412	-3.079773

$Ph_3Si^{\bullet}$
E = -4886.84
H = -4709.74
N_{imag} = 0
S = 1/2

Si	1.788262	0.115895	-1.278450
C	1.358545	1.399022	-2.579959
C	1.023720	2.718890	-2.195308
C	0.692720	3.687485	-3.144826
C	0.673489	3.359140	-4.506538
C	0.991934	2.056847	-4.908664
C	1.329545	1.088833	-3.958589
C	3.184896	0.652523	-0.144105
C	3.890322	1.861135	-0.342137
C	4.919304	2.245462	0.522303

C	5.269421	1.433493	1.607301
C	4.580504	0.233249	1.824626
C	3.548465	-0.147549	0.964851
C	2.035072	-1.604096	-1.989841
C	3.211250	-2.351618	-1.754314
C	3.367477	-3.636025	-2.283065
C	2.352886	-4.207413	-3.059716
H	1.021344	2.988025	-1.140191
H	0.443645	4.697263	-2.824138
H	0.411157	4.111926	-5.246910
H	0.983523	1.795955	-5.965396
H	1.585535	0.085351	-4.290151
H	3.639851	2.498511	-1.186812
H	5.453254	3.177277	0.345029
H	6.070856	1.733359	2.279198
H	4.844945	-0.401947	2.667905
H	3.015302	-1.077619	1.155661
H	4.014838	-1.918905	-1.163174
H	4.285518	-4.189447	-2.094015
H	2.475718	-5.207021	-3.471234
H	0.382522	-3.924351	-3.903106
H	0.092880	-1.663259	-2.957338
C	1.177132	-3.485590	-3.302583
C	1.018402	-2.205159	-2.769085

(*t*-Bu)$_3$Si$^•$

E = -4719.23

H = -4483.40

$\boldsymbol{N_{imag}}$ = 0

S = 1/2

H	-4.913571	-0.198844	2.689592
Si	-4.312547	-1.585022	0.002679
C	-3.440804	0.089532	-0.592059
C	-4.454574	1.075144	-1.230513
H	-3.926783	1.984552	-1.559083
H	-4.949143	0.645168	-2.107975
H	-5.230545	1.385903	-0.524435
C	-2.757495	0.779414	0.620063
H	-3.470016	1.086399	1.388760
H	-2.230016	1.682247	0.276008
H	-2.018762	0.118596	1.089354
C	-2.324447	-0.197133	-1.627540
H	-2.715134	-0.603689	-2.564681
H	-1.806789	0.743627	-1.868501
C	-4.576636	-2.886230	-1.465372
C	-3.209803	-3.508340	-1.861497
H	-3.367695	-4.268660	-2.641688
H	-2.508196	-2.770660	-2.256935
H	-2.734043	-4.001276	-1.005169
C	-5.485526	-4.058671	-1.018179
H	-5.089326	-4.565238	-0.130572
H	-5.538954	-4.801830	-1.828062
H	-6.508535	-3.738169	-0.801632
C	-5.214434	-2.226507	-2.716280
H	-4.582184	-1.438483	-3.136402
H	-5.356486	-2.986403	-3.501088

H	-6.195153	-1.791348	-2.496651
C	-5.952074	-1.271395	1.066371
C	-7.184252	-0.997590	0.164410
H	-8.068717	-0.814835	0.795172
H	-7.042285	-0.115385	-0.468829
H	-7.417687	-1.845789	-0.486204
C	-6.240283	-2.511498	1.955898
H	-6.446122	-3.411607	1.372589
H	-7.122564	-2.312943	2.583474
H	-5.396876	-2.727226	2.622804
C	-5.775864	-0.067615	2.025771
H	-5.656997	0.880148	1.493053
H	-6.671385	0.021988	2.659072
H	-1.575630	-0.895333	-1.236133

$H_3Ge^•$
E = -279.74
H = -265.20
N_{imag} = 0
S = 1/2

Ge	0.00000000	0.00000000	0.76020800
H	0.73408900	1.27147900	1.25068000
H	0.73408900	-1.27147900	1.25068000
H	-1.46817800	0.00000000	1.25068000

$Ph_3Ge^•$
E = -4846.83
H = -4670.35
N_{imag} = 0
S = 1/2

Ge	-0.00307300	0.00110600	-1.08746600
C	-1.88032600	0.00336500	-0.46478100
C	-2.74549300	-1.04630300	-0.82976200
C	-4.07488900	-1.05952900	-0.39783600
C	-4.56547200	-0.01457500	0.39474300
C	-3.71886700	1.04055400	0.75384900
C	-2.38732200	1.05230500	0.32509600
C	0.93471800	-1.62630100	-0.46654200
C	0.28414200	-2.58636600	0.33107400
C	0.96176900	-3.73296100	0.75911900
C	2.29603500	-3.94143800	0.39147100
C	2.95145000	-2.99754000	-0.40868000
C	2.27363000	-1.85346100	-0.83964100
C	0.94022800	1.62438600	-0.46395400
C	2.09537600	1.53568900	0.33545000
C	2.75408700	2.69267200	0.76495600
C	2.27364300	3.95470000	0.39702800
H	-2.37956300	-1.86046400	-1.45332800
H	-4.72888500	-1.88165800	-0.68215600
H	-5.60098300	-0.02189800	0.72803000
H	-4.09421800	1.85392100	1.37212500
H	-1.73830200	1.87391700	0.61915400
H	-0.74966800	-2.43300700	0.63175800

H	0.44851800	-4.46197400	1.38350000
H	2.82134400	-4.83404700	0.72423200
H	3.98832900	-3.15497300	-0.69946000
H	2.79246200	-1.13186300	-1.46872800
H	2.47494200	0.56186600	0.63593200
H	3.64080300	2.60845100	1.39056000
H	2.78754900	4.85356500	0.73075800
H	0.75305500	5.03411500	-0.69610200
H	-0.40892500	2.99242700	-1.46817800
C	1.13021100	4.05563800	-0.40491800
C	0.47369600	2.89968200	-0.83730200

(*t*-Bu)$_3$Ge$^•$
E = -4689.22
H = -4454.25
N_{imag} = 0
S = 1/2

H	-1.04872000	-2.87795800	1.35900200
Ge	-0.00017500	-0.00026300	0.73374300
C	-1.93969300	0.52790200	0.12709700
C	-2.16390000	0.16107000	-1.35353800
H	-3.18265500	0.45629300	-1.65681200
H	-1.46191700	0.67608800	-2.01785500
H	-2.07203900	-0.91485600	-1.53175600
C	-2.94769000	-0.22511900	1.01925500
H	-2.87563100	-1.31041500	0.90851100
H	-3.97417100	0.06942100	0.74710800
H	-2.80310600	0.01620700	2.07924900
C	-2.15205300	2.04056500	0.32303900
H	-1.51141700	2.64033200	-0.33047800
H	-3.19647100	2.29783600	0.08356400
C	1.42677000	1.41571800	0.12706000
C	1.27837600	2.66534200	1.01898300
H	2.04672200	3.40698900	0.74681300
H	0.30250400	3.14556800	0.90793200
H	1.41489500	2.41972300	2.07905800
C	2.84309700	0.84369400	0.32314000
H	3.01632600	0.53057600	1.35941600
H	3.58792800	1.61953200	0.08302800
H	3.04225000	-0.01142400	-0.32981500
C	1.22119400	1.79315400	-1.35364300
H	0.24336100	2.25121100	-1.53202300
H	1.98600100	2.52808000	-1.65686800
H	1.31661700	0.92772500	-2.01792300
C	0.51274900	-1.94379300	0.12700300
C	0.94269400	-1.95423400	-1.35358800
H	1.19667100	-2.98400900	-1.65698400
H	0.14566800	-1.60389500	-2.01794700
H	1.82842300	-1.33650400	-1.53160800
C	1.66900400	-2.44005500	1.01912700
H	2.57274300	-1.83481600	0.90840500
H	1.92735700	-3.47619800	0.74685400
H	1.38774400	-2.43566300	2.07912200

C	-0.69076800	-2.88443600	0.32279900
H	-1.53079000	-2.62937300	-0.33029300
H	-0.39113500	-3.91736100	0.08270800
H	-1.96676300	2.34734400	1.35911600

$H_3Sn^{\bullet}$

E = -255.74

H = -242.71

N_{imag} = 0

S = 1/2

Sn	0.00000000	0.00000000	0.76965800
H	0.81808400	1.41696300	1.36995200
H	0.81808400	-1.41696300	1.36995200
H	-1.63616800	0.00000000	1.36995200

$Ph_3Sn^{\bullet}$

E = -4819.20

H = -4643.27

N_{imag} = 0

S = 1/2

Sn	0.00056700	0.00093000	-1.47339100
C	-2.03463300	-0.01095700	-0.62711700
C	-2.92756000	-1.06223900	-0.90092800
C	-4.20284400	-1.08413100	-0.32429300
C	-4.60594700	-0.04659600	0.52433200
C	-3.72971400	1.01038000	0.79482200
C	-2.45326100	1.03101900	0.21930500
C	1.02724700	-1.75601200	-0.62623700
C	0.33552000	-2.63161000	0.22945900
C	0.99031800	-3.72599700	0.80784000
C	2.34123000	-3.96373300	0.53090800
C	3.03669500	-3.10397200	-0.32715600
C	2.38142200	-2.01111600	-0.90653400
C	1.00853600	1.76824700	-0.62556900
C	2.12138200	1.60736700	0.21888500
C	2.74195600	2.72125100	0.79775300
C	2.26373300	4.00935700	0.53264900
H	-2.63240500	-1.87595400	-1.56213600
H	-4.88075000	-1.90843900	-0.53851200
H	-5.59813300	-0.06093000	0.97040900
H	-4.03793800	1.81890700	1.45541000
H	-1.78146900	1.85695200	0.44532400
H	-0.71369300	-2.45716600	0.46029900
H	0.44519700	-4.39083700	1.47560800
H	2.84882900	-4.81521700	0.97917600
H	4.08745500	-3.28471400	-0.54661700
H	2.93747000	-1.35511200	-1.57504700
H	2.50146500	0.61194400	0.44083400
H	3.59712200	2.58182300	1.45680400
H	2.74739000	4.87440700	0.98140600
H	0.78718900	5.18131600	-0.52460500
H	-0.30900400	3.22137100	-1.55434700
C	1.16274900	4.18147600	-0.31432100

C	0.54387600	3.06798500	-0.89441200

(*t*-Bu)$_3$Sn$^•$

E = -4666.60

H = -4432.68

N$_{imag}$ = 0

S = 1/2

H	-0.97376200	-3.11018000	1.37815900
Sn	0.00020200	-0.00001900	0.94786900
C	-2.11131500	0.57532500	0.15692100
C	-2.21863000	0.18998700	-1.32784600
H	-3.21480700	0.47259200	-1.71328100
H	-1.47375400	0.70198200	-1.94710500
H	-2.10733900	-0.88851900	-1.48430100
C	-3.16053300	-0.18579700	0.98332900
H	-3.05245400	-1.27220200	0.89112200
H	-4.17474000	0.07619800	0.63463100
H	-3.10307900	0.06926600	2.04858100
C	-2.30721100	2.08922600	0.33061800
H	-1.59423300	2.67108800	-0.26384700
H	-3.32034600	2.37576900	-0.00163500
C	1.55407000	1.54102200	0.15679400
C	1.41916600	2.83036900	0.98295600
H	2.15321500	3.57769200	0.63432300
H	0.42426900	3.27986500	0.89034400
H	1.61103400	2.65331800	2.04830600
C	2.96312500	0.95389700	0.33091600
H	3.18023900	0.71229900	1.37858400
H	3.71790500	1.68804900	-0.00114000
H	3.11083700	0.04546600	-0.26340600
C	1.27405200	1.82642300	-1.32803900
H	0.28434200	2.26913300	-1.48457200
H	2.01678600	2.54790900	-1.71353600
H	1.34515800	0.92531000	-1.94721700
C	0.55741700	-2.11638300	0.15669200
C	0.94470000	-2.01658300	-1.32810200
H	1.19798700	-3.02056300	-1.71364400
H	0.12884300	-1.62742900	-1.94729000
H	1.82308500	-1.38097000	-1.48455600
C	1.74116400	-2.64467100	0.98297700
H	2.62812500	-2.00810200	0.89053100
H	2.02104700	-3.65411900	0.63434800
H	1.49169400	-2.72226800	2.04827900
C	-0.65582200	-3.04286200	0.33053000
H	-1.51624300	-2.71629000	-0.26386100
H	-0.39759400	-4.06359800	-0.00164000
H	-2.20659700	2.39836900	1.37820700

H$_3$Pb$^•$

E = -232.55

H = -220.58

N$_{imag}$ = 0

S = 1/2

Pb	0.00000000	0.00000000	0.63693400
H	0.85198900	1.47568800	1.29177100
H	0.85198900	-1.47568800	1.29177100
H	-1.70397800	0.00000000	1.29177100

$Ph_3Pb^{•}$
E = -4795.97
H = -4620.58
$\boldsymbol{N_{imag}}$ = 0
S = 1/2

Pb	0.00006900	0.00186300	-1.67508500
C	-2.10944400	-0.01550200	-0.70666100
C	-3.00657300	-1.06665500	-0.94113400
C	-4.24560200	-1.09541200	-0.28689000
C	-4.59596400	-0.06627700	0.59469500
C	-3.70676400	0.99006600	0.82232800
C	-2.46712100	1.01979100	0.16839100
C	1.06786100	-1.81688800	-0.70508300
C	0.35210200	-2.63448000	0.18075100
C	0.99627800	-3.69331900	0.83588200
C	2.35225500	-3.94517800	0.59871300
C	3.06619500	-3.13706100	-0.29364200
C	2.42325300	-2.07804200	-0.94881100
C	1.04096600	1.83515600	-0.70356100
C	2.11462600	1.62341300	0.17274400
C	2.71070800	2.70945000	0.82880900
C	2.24414200	4.00920700	0.60223700
H	-2.74880600	-1.87449800	-1.62481400
H	-4.93437600	-1.91931700	-0.46607200
H	-5.55914800	-0.08635000	1.10016900
H	-3.97530500	1.79179400	1.50829200
H	-1.78298200	1.84501300	0.35845900
H	-0.70204700	-2.44714400	0.37778200
H	0.43780900	-4.31905900	1.53014400
H	2.85014700	-4.76939400	1.10507200
H	4.12141300	-3.32942000	-0.48028800
H	2.99238600	-1.45858400	-1.64079100
H	2.48452300	0.61712300	0.36178100
H	3.53794100	2.53808400	1.51565200
H	2.70985400	4.85156400	1.10935900
H	0.81332900	5.23382200	-0.45911300
H	-0.24408800	3.32291500	-1.62172200
C	1.17954200	4.22418400	-0.28064900
C	0.58282300	3.13913600	-0.93694300

(*t*-Bu)$_3$Pb$^{•}$
E = -4651.31
H = -4418.11
$\boldsymbol{N_{imag}}$ = 0
S = 1/2

H	-0.94315600	-3.21022900	1.37587000
Pb	-0.00000200	0.00014400	1.04822000
C	-2.21237700	0.60382200	0.15723000

C	-2.24707800	0.19625600	-1.31709900
H	-3.22698900	0.46359600	-1.75671100
H	-1.47917000	0.70527000	-1.91039800
H	-2.12148800	-0.88393900	-1.45116600
C	-3.25452900	-0.16249500	0.97293300
H	-3.13031400	-1.24820500	0.88752100
H	-4.27167700	0.08094100	0.61338600
H	-3.21527900	0.09978300	2.03748900
C	-2.38034700	2.11419700	0.32456500
H	-1.63535100	2.68067800	-0.24564000
H	-3.37627500	2.42713500	-0.04087500
C	1.62919500	1.61421400	0.15746300
C	1.48717200	2.89952100	0.97387300
H	2.20682000	3.65861400	0.61466100
H	0.48495200	3.33519100	0.88871400
H	1.69469100	2.73380100	2.03833800
C	3.02106000	1.00386600	0.32405500
H	3.25212400	0.78740500	1.37432400
H	3.79022900	1.70985800	-0.04105700
H	3.13869700	0.07579800	-0.24680300
C	1.29311900	1.84899400	-1.31660900
H	0.29501700	2.28093200	-1.45000100
H	2.01479300	2.56380800	-1.75614700
H	1.34923300	0.92974800	-1.91043400
C	0.58324500	-2.21795000	0.15778100
C	0.95364600	-2.04456900	-1.31656300
H	1.21211100	-3.02696400	-1.75593700
H	0.12893400	-1.63416500	-1.91002700
H	1.82635300	-1.39573100	-1.45068200
C	1.76788700	-2.73731000	0.97358500
H	2.64620600	-2.08716200	0.88785400
H	2.06533100	-3.74011400	0.61435400
H	1.52117600	-2.83403500	2.03819300
C	-0.64091200	-3.11846800	0.32537100
H	-1.50395500	-2.75665800	-0.24496700
H	-0.41405800	-4.13758500	-0.03970300
H	-2.30903700	2.42200700	1.37503900

Table S10. Cartesian coordinates (in Å), energies (electronic *E* and enthalpy *H*, in kcal mol^{-1}), number of imaginary frequencies (N_{imag}), and total spin number (*S*) of the $R_3A–AR_3$ systems studied herein, computed at M06-2X/TZ2P.

$H_3C–CH_3$ (1C)
E = -1239.62
H = -1189.73
$\boldsymbol{N_{imag}}$ = 0
S = 0

C	0.00000000	0.00000000	0.76227400
H	0.00000000	1.01550400	1.15647000
H	0.87945200	-0.50775200	1.15647000
H	-0.87945200	-0.50775200	1.15647000
C	0.00000000	0.00000000	-0.76227400
H	-0.87945200	0.50775200	-1.15647000
H	0.00000000	-1.01550400	-1.15647000
H	0.87945200	0.50775200	-1.15647000

$Me_3C–CMe_3$ (2C)
E = -4283.98
H = -4122.56
$\boldsymbol{N_{imag}}$ = 0
S = 0

C	0.00000000	0.00000000	0.78551300
C	1.13704600	0.86902000	1.33887900
C	0.18407100	-1.41922100	1.33887900
C	-1.32111700	0.55020100	1.33887900
C	0.00000000	0.00000000	-0.78551300
C	0.18407100	1.41922100	-1.33887900
C	-1.32111700	-0.55020100	-1.33887900
C	1.13704600	-0.86902000	-1.33887900
H	2.10332700	-0.60004700	-0.91072700
H	1.20722200	-0.73514400	-2.41944200
H	0.96351200	-1.92793900	-1.14862600
H	-1.24026500	-0.67791300	-2.41944200
H	-2.15140000	0.12954300	-1.14862600
H	-1.57132000	-1.52151100	-0.91072700
H	1.18788800	1.79839500	-1.14862600
H	-0.53200700	2.12155800	-0.91072700
H	0.03304300	1.41305700	-2.41944200
H	-1.24026500	0.67791300	2.41944200
H	-2.15140000	-0.12954300	1.14862600
H	-1.57132000	1.52151100	0.91072700
H	2.10332700	0.60004700	0.91072700
H	1.20722200	0.73514400	2.41944200
H	0.96351200	1.92793900	1.14862600
H	1.18788800	-1.79839500	1.14862600
H	-0.53200700	-2.12155800	0.91072700
H	0.03304300	-1.41305700	2.41944200

$PhMe_2C–CMe_2Ph$ (3C)
E = -7494.05
H = -7261.86
$\boldsymbol{N_{imag}}$ = 0
S = 0

C	0.67297600	-0.42861400	0.00576100
C	0.75022500	-1.30444300	-1.25666800

C	0.74238700	-1.31082200	1.25707200
C	1.90902100	0.48635500	-0.03911200
H	1.78095600	-1.21394400	4.49406200
H	-0.10060100	-4.41330100	2.37103600
H	0.88200700	-3.52421200	4.46516500
H	2.80885900	-0.11414200	0.09613100
H	1.89864500	1.25429500	0.73025100
H	1.98665800	0.98205100	-1.00535200
H	-0.12373700	-1.93452300	-1.40188900
H	1.62280200	-1.95514300	-1.19317700
H	0.86399700	-0.68394800	-2.14382600
H	-0.18188400	-3.03560200	0.36402500
C	1.29707200	-0.83398200	2.44663300
C	1.34756900	-1.61856000	3.58928400
C	0.84259800	-2.91036000	3.57567900
C	0.29258600	-3.40598500	2.40366400
C	0.24835200	-2.61611300	1.26219300
H	1.68299300	0.17423500	2.49677800
C	-0.67297600	0.42861400	0.00576100
C	-0.74238700	1.31082200	1.25707200
C	-1.90902100	-0.48635500	-0.03911200
C	-0.75022500	1.30444300	-1.25666800
H	0.12373700	1.93452300	-1.40188900
H	-1.62280200	1.95514300	-1.19317700
H	-0.86399700	0.68394800	-2.14382600
H	-2.80885900	0.11414200	0.09613100
H	-1.89864500	-1.25429500	0.73025100
H	-1.98665800	-0.98205100	-1.00535200
H	0.18188400	3.03560200	0.36402500
C	-1.29707200	0.83398200	2.44663300
C	-1.34756900	1.61856000	3.58928400
C	-0.84259800	2.91036000	3.57567900
C	-0.29258600	3.40598500	2.40366400
C	-0.24835200	2.61611300	1.26219300
H	-1.68299300	-0.17423500	2.49677800
H	-1.78095600	1.21394400	4.49406200
H	-0.88200700	3.52421200	4.46516500
H	0.10060100	4.41330100	2.37103600

$Ph_2MeC–CMePh_2$ (4C)
E = -10696.37
H = -10392.34
N_{imag} = 0
S = 0

C	-0.57794800	2.44868500	-1.06031200
H	-0.46523800	0.32861800	-3.67055800
H	-1.25101300	2.31312100	-4.84331900
C	0.17386200	-0.06835600	-1.11761500
H	-1.65366600	4.42630300	-3.60029700
H	-1.21101900	4.48874300	-1.16325200
H	-0.37433400	2.53712400	-0.00676800
H	0.61126900	-2.73644500	-1.76875200
C	-0.84085300	-1.17015700	-1.50417100
C	-2.20985300	-0.89780400	-1.55570700
C	-3.13390200	-1.88163100	-1.87259400
C	-2.70956200	-3.17103300	-2.16327100
C	-1.35452700	-3.45720800	-2.13170600
C	-0.43459100	-2.46883800	-1.80525700

H	-2.56098900	0.10558300	-1.35406700
H	-4.18683800	-1.63557900	-1.90105500
C	1.51906100	-0.38452900	-1.81459600
H	-3.42674000	-3.93948900	-2.41765800
H	1.98337700	-1.27929600	-1.41437300
H	2.23172500	0.42620500	-1.71262700
H	1.35130800	-0.54693600	-2.87711600
H	-1.00360800	-4.45540500	-2.35623300
C	-0.34488400	1.25341200	-1.73457000
C	-0.60334200	1.24472800	-3.11164100
C	-1.06322100	2.36547600	-3.77938500
C	-1.29073400	3.54733400	-3.08553200
C	-1.04505900	3.57942000	-1.72512100
C	1.60040400	0.85739200	2.44346300
H	1.90439900	2.03809700	-0.69922900
C	2.95268300	2.76405600	0.99290400
H	3.47377300	3.50852900	0.40610900
C	0.34256600	-0.08012100	0.48316000
C	2.05961700	1.90500100	0.35785900
H	3.86484300	3.34205100	2.84701300
C	2.48267100	1.71041900	3.08167900
H	2.63813600	1.61710800	4.14802300
H	1.10210200	0.09516800	3.02776700
C	3.17083400	2.67424100	2.35541800
H	-1.14474400	-2.31550000	1.20805200
C	0.81967200	-1.48895400	0.90820800
C	2.18053300	-1.79917000	0.96139600
C	2.62288600	-3.06499800	1.31380200
C	1.70972700	-4.05860600	1.63934700
C	0.35603800	-3.76626100	1.60624600
C	-0.08074700	-2.49823900	1.24422800
H	2.91034200	-1.03372700	0.73289400
H	3.68447000	-3.27050600	1.34263700
H	2.05113400	-5.04519600	1.92159200
H	-0.37130200	-4.52640400	1.85746300
C	1.36074200	0.93070100	1.06489200
C	-1.00985700	0.20269900	1.18049700
H	-0.91663300	0.01769900	2.24844500
H	-1.80209700	-0.43530300	0.80414100
H	-1.32909900	1.23059600	1.04967000

$Ph_3C–CPh_3$ (5C)
E = -13891.16
H = -13516.93
N_{imag} = 0
S = 0

C	0.98190500	0.05288500	-0.70240100
C	0.76959700	1.10432700	-1.82949100
C	0.46085800	2.44191200	-1.53877600
C	0.23630000	3.37434400	-2.53717300
C	0.34245600	3.01767100	-3.87532100
C	0.71701700	1.72373000	-4.18457400
C	0.93531100	0.78837900	-3.17726300
C	2.22638900	0.47844000	0.12891000
C	3.00271100	1.58441300	-0.21453700
C	4.09872100	1.97925800	0.54706600
C	4.46824500	1.26660600	1.67213700
C	3.75096600	0.12222000	1.99624900

C	2.66546600	-0.26996900	1.23167400
C	1.29145300	-1.31775100	-1.37061400
C	2.53628800	-1.93426900	-1.25194500
C	2.80199300	-3.17297300	-1.82832300
C	1.83305700	-3.83086800	-2.56214300
H	0.40449600	2.78133500	-0.51900100
H	-0.01487800	4.38958900	-2.26096400
H	0.16181100	3.74355600	-4.65627600
H	0.84639600	1.42452000	-5.21595200
H	1.23818600	-0.20106400	-3.47559400
H	2.76853800	2.16913700	-1.08803600
H	4.66055100	2.85287800	0.24516000
H	5.31281500	1.57732000	2.27180900
H	4.03978300	-0.48074400	2.84662000
H	2.17024900	-1.18600900	1.50360700
H	3.33248900	-1.46137600	-0.70223600
H	3.78035600	-3.61484300	-1.69577100
H	2.03334800	-4.79814700	-3.00224700
H	-0.15843500	-3.67589100	-3.35343400
H	-0.60167700	-1.51415500	-2.39090000
C	0.60686200	-3.20620100	-2.75025900
C	0.34970900	-1.97107100	-2.18014500
C	-0.39278600	-0.05580900	0.28529100
C	-0.18052500	-1.10723500	1.41240900
C	0.12834200	-2.44480800	1.12176900
C	0.35288300	-3.37718500	2.12022200
C	0.24658900	-3.02047100	3.45834700
C	-0.12808800	-1.72654500	3.76752300
C	-0.34636500	-0.79124800	2.76016100
C	-0.70239400	1.31479800	0.95353400
C	0.23936900	1.96822500	1.76295500
C	-0.01792600	3.20324900	2.33323800
C	-1.24428200	3.82769400	2.14541300
C	-2.21321600	3.16968800	1.41169200
C	-1.94736700	1.93109200	0.83514200
C	-1.63719500	-0.48137600	-0.54611700
C	-2.07635800	0.26723200	-1.64871300
C	-3.16169100	-0.12502400	-2.41349300
C	-3.87870800	-1.26967900	-2.08975700
C	-3.50912600	-1.98252800	-0.96483000
C	-2.41328700	-1.58761200	-0.20302200
H	0.18482600	-2.78427000	0.10201200
H	0.60416500	-4.39242000	1.84406900
H	0.42722600	-3.74631400	4.23934300
H	-0.25756400	-1.42730400	4.79888000
H	-0.64930600	0.19819300	3.05843400
H	1.19088500	1.51148000	1.97348500
H	0.74738600	3.67302900	2.93632300
H	-1.44468700	4.79488500	2.58565800
H	-3.19169200	3.61137600	1.27936200
H	-2.74358000	1.45808400	0.28554900
H	-1.58134300	1.18347200	-1.92033700
H	-3.45058400	0.47809400	-3.26372700
H	-4.72314000	-1.58044600	-2.68959600
H	-4.07077300	-2.85636000	-0.66320300
H	-2.17906200	-2.17250400	0.67034700

$H_3Si–SiH_3$ (1Si)
E = -973.81
H = -938.86
N_{imag} = 0
S = 0

Si	0.00000000	0.00000000	1.16830600
H	1.20170000	0.69380200	1.67854300
H	0.00000000	-1.38760400	1.67854300
H	-1.20170000	0.69380200	1.67854300
Si	0.00000000	0.00000000	-1.16830600
H	-1.20170000	-0.69380200	-1.67854300
H	1.20170000	-0.69380200	-1.67854300
H	0.00000000	1.38760400	-1.67854300

$Me_3Si–SiMe_3$ (2Si)
E = -4075.82
H = -3926.24
N_{imag} = 0
S = 0

Si	1.17141000	0.00008300	0.00000000
Si	-1.17141000	-0.00008300	0.00000000
C	1.81527700	-0.88432800	-1.53188900
C	1.81588600	1.76874100	0.00000000
C	1.81527700	-0.88432800	1.53188900
C	-1.81527700	0.88432800	1.53188900
C	-1.81527700	0.88432800	-1.53188900
C	-1.81588600	-1.76874100	0.00000000
H	1.47467700	-0.39152400	-2.44352900
H	2.90671900	-0.89230000	-1.54353100
H	1.47289700	-1.91960500	-1.56224500
H	1.47468600	2.31242600	0.88200300
H	2.90734000	1.78227900	0.00000000
H	1.47468600	2.31242600	-0.88200300
H	1.47289700	-1.91960500	1.56224500
H	2.90671900	-0.89230000	1.54353100
H	1.47467700	-0.39152400	2.44352900
H	-1.47289700	1.91960500	1.56224500
H	-2.90671900	0.89230000	1.54353100
H	-1.47467700	0.39152400	2.44352900
H	-1.47467700	0.39152400	-2.44352900
H	-2.90671900	0.89230000	-1.54353100
H	-1.47289700	1.91960500	-1.56224500
H	-1.47468600	-2.31242600	0.88200300
H	-2.90734000	-1.78227900	0.00000000
H	-1.47468600	-2.31242600	-0.88200300

$PhMe_2Si–SiMe_2Ph$ (3Si)
E = -7287.08
H = -7064.85
N_{imag} = 0
S = 0

C	1.67616600	-0.98385200	1.56967900
H	3.11245900	-1.22616600	-4.53444700
H	1.19069600	-1.95634600	1.66141000
H	2.75506100	-1.14165500	1.60142000
H	1.39343000	-0.38315900	2.43552600
H	2.33941800	-3.57456800	-4.51580300
H	1.08994500	-4.44105600	-2.56281800

C	1.59777300	-1.25160600	-1.48518500
C	2.29795600	-0.78307700	-2.59976800
C	2.56548400	-1.61020300	-3.68356700
C	2.13254300	-2.92840100	-3.67323500
C	1.43277200	-3.41495300	-2.57643400
C	1.16907600	-2.58419300	-1.49692900
H	2.64670900	0.24268300	-2.62522100
H	0.61232000	-2.98170400	-0.65479000
H	1.89707800	2.13933400	0.64740600
H	3.17465400	1.33331100	-0.26425100
H	1.78840600	2.03283500	-1.10761600
Si	1.17786300	-0.13454900	-0.03146300
C	2.09679100	1.49541600	-0.21007000
Si	-1.14693200	0.16392300	-0.09384800
H	-1.29259800	2.01692500	-1.73207900
C	-1.77038000	1.17184200	1.36584200
C	-1.63723800	0.98220300	-1.71111800
H	-1.33767000	2.17315100	1.35393000
H	-2.85619900	1.26987300	1.33135700
H	-1.50478900	0.70705400	2.31582100
H	-1.19026900	0.46696200	-2.56249600
H	-2.72065000	0.98445300	-1.84066100
C	-1.87543400	-1.56873400	-0.02266000
C	-1.88899900	-2.28613700	1.17823400
C	-2.32546400	-3.60267100	1.22810300
C	-2.76010700	-4.23286800	0.06886500
C	-2.75953100	-3.53869100	-1.13279900
C	-2.32043300	-2.22160400	-1.17476900
H	-1.54927000	-1.81274000	2.09323400
H	-2.32734300	-4.13733400	2.16868200
H	-3.09986900	-5.25922400	0.10372700
H	-3.09844600	-4.02377700	-2.03854600
H	-2.31710200	-1.70023600	-2.12452000

$Ph_2MeSi–SiMePh_2$ (4Si)
E = -10498.73
H = -10204.65
N_{imag} = 0
S = 0

C	-0.80337900	2.68885700	-1.77113600
H	-0.46302000	0.81234000	-4.56386200
H	-1.30103100	2.87984700	-5.58662900
Si	0.15843400	-0.03206900	-1.81090300
H	-1.83492700	4.83898600	-4.17408100
H	-1.51712900	4.71160700	-1.72547700
H	-0.66712800	2.65542000	-0.69568200
H	0.37234700	-2.99479800	-1.85360800
C	-1.04618800	-1.41450900	-2.23083600
C	-2.37616900	-1.15141900	-2.57129600
C	-3.27383000	-2.18371600	-2.81084600
C	-2.85651100	-3.50390000	-2.71144000
C	-1.54049100	-3.78709900	-2.37068800
C	-0.64731200	-2.75194700	-2.13222900
H	-2.71451800	-0.12516300	-2.65633500
H	-4.29792200	-1.95766600	-3.07664900
C	1.84515800	-0.44161100	-2.52779400
H	-3.55405900	-4.30927700	-2.89882100
H	2.21494000	-1.38693000	-2.12826500

H	2.57045900	0.33385700	-2.27982400
H	1.79231500	-0.52838900	-3.61384600
H	-1.20987100	-4.81389100	-2.28752300
C	-0.49155400	1.57180900	-2.54849200
C	-0.68281900	1.66586400	-3.93172900
C	-1.16094200	2.82966900	-4.51510900
C	-1.46152900	3.93013600	-3.72170700
C	-1.28312200	3.85873400	-2.34835100
C	1.96124100	1.09634300	2.52761700
H	1.82139500	2.28932900	-0.63763200
C	3.04693400	3.07765300	0.92310400
H	3.47070900	3.84699900	0.29168000
Si	0.26529700	-0.01879100	0.53202300
C	2.11248500	2.19008400	0.40260500
H	4.16428500	3.66243000	2.65755000
C	2.89243100	1.97924700	3.05368300
H	3.19713100	1.89155100	4.08799900
H	1.55507100	0.31703200	3.16318600
C	3.43639800	2.97376000	2.25005500
H	-1.17335000	-2.53383900	1.27306900
C	0.81432100	-1.76265300	0.97181300
C	2.16742400	-2.11462100	0.93048300
C	2.57654100	-3.42287800	1.14849200
C	1.63468200	-4.40867300	1.41529700
C	0.28731600	-4.07938700	1.46551100
C	-0.11595200	-2.76910300	1.24355200
H	2.91289500	-1.35258700	0.72992100
H	3.62838900	-3.67363400	1.11366000
H	1.95105400	-5.42883300	1.58681000
H	-0.45017400	-4.84303400	1.67386400
C	1.54867500	1.18656900	1.19352600
C	-1.42026600	0.30134000	1.29494200
H	-1.40119300	0.09871300	2.36691900
H	-2.17862100	-0.33547200	0.83699500
H	-1.72418000	1.33778100	1.15027700

$Ph_3Si–SiPh_3$ (5Si)
E = -13713.00
H = -13346.98
N_{imag} = 0
S = 0

Si	1.49141500	0.09476800	-1.07062900
C	1.15964900	1.38067200	-2.39927500
C	0.78125500	2.67898000	-2.03286500
C	0.47027100	3.63157300	-2.99228600
C	0.52841900	3.30322900	-4.34136900
C	0.90460100	2.02390600	-4.72358700
C	1.21748300	1.07268200	-3.75987100
C	2.94785900	0.60838000	-0.00120500
C	3.67528700	1.77389100	-0.24994200
C	4.70661800	2.16847300	0.59393000
C	5.02942300	1.40093400	1.70341600
C	4.32095800	0.23450500	1.96560900
C	3.29026300	-0.15498400	1.12265400
C	1.79639100	-1.59062500	-1.84218500
C	2.98753400	-2.29225500	-1.64640300
C	3.16193100	-3.56771600	-2.17011000
C	2.14541700	-4.16395900	-2.90190600

H	0.72101200	2.95014300	-0.98385300
H	0.17870600	4.62778700	-2.68760700
H	0.28241100	4.04353400	-5.09081300
H	0.95487300	1.76459500	-5.77268000
H	1.50746900	0.07720200	-4.07323100
H	3.43334600	2.38434100	-1.11138700
H	5.25744700	3.07556200	0.38381900
H	5.83074300	1.70835700	2.36190700
H	4.56818800	-0.36900800	2.82869600
H	2.74078700	-1.06305800	1.34865900
H	3.78950000	-1.84025800	-1.07560600
H	4.09232700	-4.09481200	-2.00606800
H	2.27961400	-5.15757500	-3.30805600
H	0.15808200	-3.93865400	-3.68393200
H	-0.15796200	-1.69276100	-2.75252600
C	0.95438500	-3.47984500	-3.11326000
C	0.78261500	-2.20793600	-2.58666400
Si	-0.41500000	-0.05862000	0.30298100
C	-0.08332600	-1.34455400	1.63162100
C	0.29512200	-2.64284800	1.26521900
C	0.60595700	-3.59547900	2.22465200
C	0.54760200	-3.26718800	3.57373900
C	0.17137000	-1.98787800	3.95594900
C	-0.14136000	-1.03661600	2.99222100
C	-0.71988100	1.62678500	1.07455400
C	0.29388800	2.24398300	1.81913500
C	0.12220000	3.51590500	2.34572600
C	-1.06873800	4.20014800	2.13426000
C	-2.08524500	3.60401800	1.40236300
C	-1.91093200	2.32854300	0.87866400
C	-1.87147400	-0.57214800	-0.76644700
C	-2.21383500	0.19120800	-1.89032400
C	-3.24457000	-0.19823000	-2.73325500
C	-3.95311900	-1.36459700	-2.47102100
C	-3.63035400	-2.13213000	-1.36151800
C	-2.59898300	-1.73760200	-0.51767100
H	0.35551200	-2.91397600	0.21620700
H	0.89756400	-4.59168200	1.91997900
H	0.79349000	-4.00752400	4.32319300
H	0.12093800	-1.72860800	5.00504500
H	-0.43139500	-0.04114900	3.30557600
H	1.23439200	1.72870500	1.98508800
H	0.91849500	3.97462200	2.91648000
H	-1.20287000	5.19377500	2.54040400
H	-3.01556800	4.13121400	1.23823700
H	-2.71289300	1.87663300	0.30779100
H	-1.66430100	1.09923600	-2.11636400
H	-3.49176600	0.40527600	-3.59635600
H	-4.75447100	-1.67197900	-3.12949100
H	-4.18124900	-3.03917200	-1.15137400
H	-2.35707600	-2.34804600	0.34378700

***t*-Bu$_3$Si–Si*t*-Bu$_3$ (6Si)**
E = -13143.59
N$_{imag}$ = 0
S = 0

H	-1.33157400	-0.14708400	3.23014600
Si	-2.26687200	-2.95284600	1.98922200

C	-2.25760600	-2.06964500	3.76165800
C	-3.50852600	-2.43658500	4.57610700
H	-3.48328900	-1.88945800	5.52358800
H	-4.43056100	-2.15698200	4.07213800
H	-3.55834700	-3.49475300	4.81953700
C	-1.04393200	-2.45711900	4.63010100
H	-0.92687800	-3.52737500	4.77052600
H	-1.18322500	-2.01242200	5.62076200
H	-0.11111200	-2.06036000	4.23593100
C	-2.23164600	-0.53671200	3.69892600
H	-3.08536700	-0.12712400	3.17168500
H	-2.25842500	-0.14640200	4.72136700
C	-2.69987300	-4.86078600	2.29578900
C	-4.18827800	-5.13341900	2.55044600
H	-4.32747600	-6.21183800	2.67726900
H	-4.55847700	-4.65370000	3.45294200
H	-4.81629500	-4.81902100	1.72501300
C	-2.31525900	-5.72373600	1.08331200
H	-2.82196300	-5.41248800	0.17299200
H	-2.61573500	-6.75770600	1.27908300
H	-1.24497100	-5.73254400	0.89392600
C	-1.96675000	-5.46536500	3.51000400
H	-2.32189200	-5.05517900	4.45263300
H	-2.18028000	-6.53891000	3.53179200
H	-0.88753900	-5.35182100	3.47383000
C	-0.41221700	-2.88395600	1.29836100
C	0.55000900	-3.83507900	2.03785100
H	1.56194100	-3.64703900	1.66476800
H	0.57092100	-3.69082500	3.11379100
H	0.33074300	-4.88141000	1.83775500
C	-0.28084600	-3.25519000	-0.18470200
H	-0.57813700	-4.27987400	-0.39287300
H	0.77004100	-3.15810800	-0.47564200
H	-0.85869700	-2.60360100	-0.82976500
C	0.18336500	-1.47337500	1.43372000
H	0.28525700	-1.15573000	2.46821500
H	1.18676400	-1.47273700	0.99680900
H	-0.39599700	-0.72358500	0.90036400
H	-4.96111200	-0.43863000	2.93318700
Si	-3.99869400	-1.79669200	0.30995100
C	-3.34092600	-0.04349200	-0.33464300
C	-4.43625000	0.81103200	-1.00346500
H	-3.95868400	1.70279600	-1.42210300
H	-4.95091600	0.30879100	-1.81695900
H	-5.18095800	1.15857000	-0.29102600
C	-2.75282100	0.84956600	0.76586500
H	-3.48363200	1.12869000	1.52064700
H	-2.39271300	1.77574500	0.30655900
H	-1.91212900	0.38855000	1.27111700
C	-2.22601400	-0.21407900	-1.37893600
H	-2.56833600	-0.70692100	-2.28524000
H	-1.86258100	0.77657100	-1.66916400
C	-4.33121900	-2.91437600	-1.29064100
C	-3.06231500	-3.50873900	-1.91604300
H	-3.35335300	-4.12994800	-2.76918300
H	-2.37884600	-2.75040200	-2.28954900
H	-2.51430600	-4.13962400	-1.22592400
C	-5.23720400	-4.11097300	-0.95881100

H	-4.81439700	-4.75288000	-0.18972500
H	-5.35320800	-4.72336200	-1.85838500
H	-6.23402000	-3.81159100	-0.64547300
C	-5.02376400	-2.14903700	-2.43604400
H	-4.37502100	-1.39885900	-2.88249100
H	-5.26423900	-2.86910100	-3.22487200
H	-5.95225100	-1.66718400	-2.14513300
C	-5.75490000	-1.47651200	1.16728900
C	-6.87577400	-1.15252200	0.15932000
H	-7.77211600	-0.88382400	0.72760400
H	-6.64879200	-0.31889300	-0.49827200
H	-7.13877700	-2.01047600	-0.45524400
C	-6.28147800	-2.66459000	1.98325800
H	-6.46655600	-3.54697900	1.37595000
H	-7.23574000	-2.37879200	2.43728400
H	-5.61096400	-2.94799600	2.78618000
C	-5.69293000	-0.29023000	2.14279600
H	-5.47592200	0.65263400	1.64742900
H	-6.66871300	-0.18232400	2.62643500
H	-1.37245600	-0.76463500	-0.99083400

Table S11. Cartesian coordinates (in Å), energies (electronic E and enthalpy H, in kcal mol^{-1}), number of imaginary frequencies (N_{imag}), and total spin number (S) of equilibrium geometries of the $R_3A^{\bullet}$ radical species studied herein, computed at (U)M06-2X/TZ2P.

$H_3C^{\bullet}$
E = -571.10
H = -549.88
$\boldsymbol{N_{imag}}$ = 0
S = 1/2

C	0.00000000	0.00000000	0.00000000
H	0.53811000	0.93203400	0.00000000
H	0.53811000	-0.93203400	0.00000000
H	-1.07622000	0.00000000	0.00000000

$Me_3C^{\bullet}$
E = -2101.08
H = -2022.90
$\boldsymbol{N_{imag}}$ = 0
S = 1/2

H	1.73745200	-1.23915600	0.46626500
H	-1.94186600	0.88510000	0.46626500
H	-1.94186600	-0.88510000	0.46626500
H	-1.73563600	0.00000000	-1.03932200
C	0.00000000	0.00000000	0.23081300
C	-1.47707800	0.00000000	0.02985300
C	0.73853900	-1.27918700	0.02985300
C	0.73853900	1.27918700	0.02985300
H	0.20441400	2.12425600	0.46626500
H	0.86781800	1.50310500	-1.03932200
H	1.73745200	1.23915600	0.46626500
H	0.86781800	-1.50310500	-1.03932200
H	0.20441400	-2.12425600	0.46626500

$PhMe_2C^{\bullet}$
E = -3717.38
H = -3603.29
$\boldsymbol{N_{imag}}$ = 0
S = 1/2

C	1.25693500	-0.75207100	0.00296300
C	1.53974200	-0.05075700	-1.28719200
C	0.69459600	-2.06133700	-0.00015600
C	1.56882800	-0.06938500	1.29639600
H	-0.32889800	-4.51882600	2.13333900
H	-0.38002200	-4.48642600	-2.14553100
H	-0.84756900	-5.65016100	-0.00887200
H	2.28681800	-0.64195400	1.89129400
H	0.67493000	0.04655500	1.91660300
H	1.98871200	0.91967600	1.12913400
H	0.63152900	0.07691300	-1.88390600
H	2.24149600	-0.61613500	-1.90773100
H	1.96639800	0.93458700	-1.11520500
H	0.58299100	-2.24543600	-2.15270200
C	0.41698000	-2.75017000	1.20379100

C	-0.12830600	-4.01877800	1.19507900
C	-0.42081700	-4.65699400	-0.00646700
C	-0.15694800	-4.00062700	-1.20486500
C	0.38824500	-2.73196500	-1.20740200
H	0.63443800	-2.27800800	2.15142600

$Ph_2MeC^{\bullet}$

E = -5327.25

H = -5177.33

N_{imag} = 0

S = 1/2

C	2.25295700	0.46588400	0.33819200
H	-0.03733500	1.89739200	2.38095500
C	1.56125600	2.92301700	1.41366000
H	1.29100700	3.87793600	1.84396100
C	0.32758800	-0.63380100	1.48623200
C	0.80534200	1.80209400	1.70994800
H	3.24864100	3.70359900	0.33674000
C	2.99895700	1.59061800	0.03345900
H	3.84300200	1.50473100	-0.63763800
H	2.51056600	-0.48422100	-0.10899200
C	2.66264500	2.82623700	0.57295500
H	-0.95581400	-3.00262800	1.32585700
C	0.90223700	-1.94649600	1.63901300
C	2.26573800	-2.14039300	1.94907100
C	2.78799500	-3.40614200	2.12958800
C	1.97764100	-4.53093200	2.01098100
C	0.62823900	-4.36559000	1.72472800
C	0.09663800	-3.10093000	1.55019900
H	2.90552600	-1.27989500	2.08116500
H	3.83494500	-3.51872900	2.37757800
H	2.39053400	-5.52023100	2.15074500
H	-0.01557400	-5.23052000	1.63700600
C	1.13659100	0.53991200	1.18947000
C	-1.16043200	-0.48691900	1.58835200
H	-1.55552700	-0.96802700	2.48515400
H	-1.65068100	-0.96038900	0.73148000
H	-1.46430100	0.55555200	1.58544600

$Ph_3C^{\bullet}$

E = -6939.10

E = -6753.61

N_{imag} = 0

S = 1/2

C	-1.24081400	0.00433800	0.87921300
C	-0.67709900	-1.01962000	1.75002500
C	-0.12357300	-2.19932300	1.22388300
C	0.41421600	-3.16844800	2.05213800
C	0.40927400	-2.99648900	3.43114100
C	-0.13697800	-1.83874300	3.97196100
C	-0.66770900	-0.86328700	3.14658200
C	-1.14427700	1.41083100	1.25032500
C	-0.03221400	1.90368700	1.95389700

C	0.05970600	3.23995400	2.30097700
C	-0.95806200	4.12493900	1.96605600
C	-2.06868200	3.65673900	1.27429500
C	-2.15996000	2.32306100	0.91731100
C	-1.90137900	-0.37746300	-0.36286500
C	-1.84176200	0.44410300	-1.50140400
C	-2.46546900	0.07805900	-2.68099800
C	-3.17597100	-1.11364400	-2.76022700
C	-3.25104500	-1.93828100	-1.64416800
C	-2.62104900	-1.58010200	-0.46548600
H	-0.10242100	-2.33759700	0.15154000
H	0.84624300	-4.06070600	1.61946500
H	0.82668900	-3.75638200	4.07725200
H	-0.15552600	-1.69868100	5.04435400
H	-1.10229300	0.02761900	3.57890600
H	0.77156500	1.22633000	2.20816200
H	0.93358400	3.59519300	2.83024500
H	-0.88649100	5.16816900	2.24116400
H	-2.87174200	4.33431400	1.01746000
H	-3.03458700	1.96692000	0.39031500
H	-1.28064000	1.36740100	-1.45483900
H	-2.39155200	0.72280100	-3.54634400
H	-3.66576900	-1.39666700	-3.68170900
H	-3.80997400	-2.86315100	-1.69105600
H	-2.69707900	-2.22303800	0.40074300

$H_3Si^{\bullet}$

E = -449.71

H = -433.68

N_{imag} = 0

S = 1/2

Si	0.00000000	0.00000000	0.78023500
H	0.70227400	1.21637400	1.23503200
H	0.70227400	-1.21637400	1.23503200
H	-1.40454700	0.00000000	1.23503200

$Me_3Si^{\bullet}$

E = -1999.93

H = -1925.72

N_{imag} = 0

S = 1/2

Si	0.07613000	0.27581600	0.00000000
C	0.71602300	-0.59275000	-1.53828200
C	0.63186500	2.07056600	0.00000000
C	0.71602300	-0.59275000	1.53828200
H	0.35466200	-0.10942200	-2.44550800
H	1.80888600	-0.56897800	-1.55643500
H	0.40254300	-1.63599000	-1.56354300
H	0.26883200	2.59765300	0.88183800
H	1.72370200	2.12631200	0.00000000
H	0.26883200	2.59765300	-0.88183800
H	0.40254300	-1.63599000	1.56354300
H	1.80888600	-0.56897800	1.55643500

H	0.35466200	-0.10942200	2.44550800

PhMe₂Si˙

E = -3606.18

H = -3495.63

N_{imag} = 0

S = 1/2

Si	1.32013100	0.04818900	0.00513200
C	1.63988900	-0.93189600	-1.56340400
C	1.70476000	-0.96637000	1.53679900
H	3.65975000	3.91175000	2.13798900
H	1.30890400	-0.39400600	-2.45086900
H	2.70620200	-1.14274300	-1.67191100
H	1.10561500	-1.88111600	-1.53327800
H	4.25538700	4.99837800	-0.00267800
H	3.60572300	3.93720200	-2.14037200
H	2.37153300	1.81981700	-2.14364800
H	1.16500500	-1.91261000	1.51092400
H	2.77383600	-1.18360400	1.59260800
H	1.41684200	-0.44604800	2.44938200
C	2.29891000	1.63548300	0.00201600
C	2.67614600	2.25561500	1.19917000
C	3.37505100	3.45363800	1.20009200
C	3.71072300	4.06392700	-0.00137000
C	3.34468600	3.46796600	-1.20109000
C	2.64584500	2.26983400	-1.19679600
H	2.42544600	1.79453600	2.14726500

$Ph_2MeSi^{\cdot}$

E = -5211.79

H = -5064.98

N_{imag} = 0

S = 1/2

C	-0.03165700	2.78972500	-2.42652300
H	-2.11854700	0.74152000	-4.13138400
H	-2.62983600	2.85601200	-5.26812200
Si	-0.24296400	0.02126600	-1.88001400
H	-1.47767300	4.93991000	-4.60286900
H	0.19507900	4.89071100	-2.78026800
H	0.69544500	2.78830500	-1.62171600
H	0.29343100	-2.76726900	-2.77837800
C	-1.32852400	-1.40281100	-2.39495900
C	-2.72440500	-1.28582700	-2.37274000
C	-3.53970200	-2.35343300	-2.71427800
C	-2.97561300	-3.57106500	-3.07562300
C	-1.59536400	-3.71227300	-3.09334600
C	-0.78215700	-2.63889200	-2.75544500
H	-3.17925200	-0.34574600	-2.08098000
H	-4.61532400	-2.23978500	-2.69356400
C	1.57637000	-0.39827600	-2.05345900
H	-3.61037800	-4.40635800	-3.33877900
H	1.85697100	-1.22881600	-1.40680100
H	2.18970200	0.46085600	-1.78389600

H	1.80986500	-0.66634800	-3.08614800
H	-1.15130500	-4.65848700	-3.37247700
C	-0.67069600	1.59716400	-2.78761600
C	-1.61246200	1.64716300	-3.82048300
C	-1.90100800	2.84061800	-4.46866700
C	-1.25263100	4.01026700	-4.09779500
C	-0.31312800	3.98225300	-3.07483400

$Ph_3Si^{\cdot}$

E = -6818.03

H = -6635.02

N_{imag} = 0

S = 1/2

Si	1.77248100	0.11357200	-1.26356400
C	1.36633000	1.39024500	-2.55959700
C	1.00305300	2.68525000	-2.16811300
C	0.68257900	3.65439900	-3.10608200
C	0.70696900	3.34434300	-4.46029900
C	1.05729100	2.06499300	-4.86834100
C	1.38429400	1.09809500	-3.92721500
C	3.17732000	0.63936100	-0.15719400
C	3.89719200	1.81855700	-0.37485900
C	4.92708400	2.19406100	0.47709200
C	5.25872200	1.39662900	1.56340600
C	4.55447200	0.22200000	1.79775800
C	3.52191700	-0.14708500	0.94964900
C	2.02845600	-1.58454300	-1.98805500
C	3.20286400	-2.31568200	-1.78230800
C	3.35490600	-3.58796400	-2.31680000
C	2.33581300	-4.15405600	-3.06973800
H	0.97007800	2.93803400	-1.11404400
H	0.40775800	4.64934800	-2.78243300
H	0.45254600	4.09753400	-5.19375300
H	1.08074600	1.82009400	-5.92182900
H	1.66477700	0.10663200	-4.26116700
H	3.65618400	2.44612000	-1.22415500
H	5.47456300	3.10804100	0.28929700
H	6.06176500	1.68893600	2.22647200
H	4.80685100	-0.40181700	2.64472700
H	2.97379900	-1.06105000	1.15031100
H	4.01106600	-1.88310600	-1.20537400
H	4.27258300	-4.13581100	-2.14916800
H	2.45488700	-5.14500900	-3.48664100
H	0.36244100	-3.88140200	-3.86938100
H	0.08261300	-1.63818000	-2.91176700
C	1.16101700	-3.44406200	-3.28525100
C	1.00881500	-2.17683700	-2.74435800

***t*-$Bu_3Si^{\cdot}$**

E = -6551.71

H = -6308.37

N_{imag} = 0

S = 1/2

H	-4.87377300	-0.22715700	2.63304300
Si	-4.33521600	-1.56988200	-0.01928600
C	-3.47638000	0.06270400	-0.58844700
C	-4.46069000	1.05006100	-1.23191500
H	-3.91970400	1.94171100	-1.56647800
H	-4.95995600	0.62191900	-2.10249900
H	-5.22935100	1.38004100	-0.53377100
C	-2.79640900	0.74034900	0.61290700
H	-3.50254400	1.06951900	1.37062300
H	-2.24904200	1.62288300	0.26690300
H	-2.07921300	0.06953100	1.09018300
C	-2.36145200	-0.23047100	-1.60179300
H	-2.74224900	-0.62392100	-2.54279600
H	-1.83329900	0.70096800	-1.82765700
C	-4.58101400	-2.84548600	-1.44737900
C	-3.22686100	-3.46350100	-1.83371900
H	-3.38687500	-4.23851800	-2.58999100
H	-2.53229600	-2.73860500	-2.24992300
H	-2.74736000	-3.93270500	-0.97228000
C	-5.46909000	-4.01167200	-0.99213100
H	-5.07034300	-4.49909700	-0.10053300
H	-5.50718800	-4.76080800	-1.78892700
H	-6.49306500	-3.70423200	-0.78656800
C	-5.21982100	-2.21513400	-2.69333700
H	-4.59207500	-1.43955000	-3.13098600
H	-5.36838900	-2.98461000	-3.45850100
H	-6.19410500	-1.77477000	-2.47576200
C	-5.92995100	-1.27339000	1.02750600
C	-7.16093200	-0.99147700	0.15396500
H	-8.02828000	-0.79750100	0.79405700
H	-7.01959300	-0.11685800	-0.48288400
H	-7.41346400	-1.83528700	-0.48739500
C	-6.20965700	-2.50069900	1.91091400
H	-6.43841800	-3.39441000	1.33637600
H	-7.07050500	-2.29315800	2.55438800
H	-5.35778800	-2.72453200	2.55617800
C	-5.74250400	-0.08976200	1.98655300
H	-5.63585700	0.86009100	1.46529200
H	-6.62405700	-0.01139500	2.63017500
H	-1.62956300	-0.93619000	-1.20451600
